# Exosomes and Homeostatic Synaptic Plasticity Are Linked to Each other and to Huntington's, Parkinson's, and Other Neurodegenerative Diseases by Database-Enabled Analyses of Comprehensively Curated Datasets

**DOI:** 10.3389/fnins.2017.00149

**Published:** 2017-03-31

**Authors:** James K. T. Wang, Peter Langfelder, Steve Horvath, Michael J. Palazzolo

**Affiliations:** ^1^Consultant to CHDI Foundation Princeton, NJ, USA; ^2^Department of Human Genetics, David Geffen School of Medicine, University of California Los Angeles, CA, USA; ^3^Pulmonary and Critical Care Medicine, David Geffen School of Medicine, University of California Los Angeles, CA, USA

**Keywords:** extracellular vesicles, synaptic scaling, Alzheimer's disease (AD), polyglutamine, systems biology, spinal muscular atrophy (SMA), amyotrophic lateral sclerosis (ALS), synaptic plasticity

## Abstract

Huntington's disease (HD) is a progressive and autosomal dominant neurodegeneration caused by CAG expansion in the huntingtin gene (*HTT*), but the pathophysiological mechanism of mutant HTT (mHTT) remains unclear. To study HD using systems biological methodologies on all published data, we undertook the first comprehensive curation of two key PubMed HD datasets: perturbation genes that impact mHTT-driven endpoints and therefore are putatively linked causally to pathogenic mechanisms, and the protein interactome of HTT that reflects its biology. We perused PubMed articles containing co-citation of gene IDs and MeSH terms of interest to generate mechanistic gene sets for iterative enrichment analyses and rank ordering. The HD Perturbation database of 1,218 genes highly overlaps the HTT Interactome of 1,619 genes, suggesting links between normal HTT biology and mHTT pathology. These two HD datasets are enriched for protein networks of key genes underlying two mechanisms not previously implicated in HD nor in each other: exosome synaptic functions and homeostatic synaptic plasticity. Moreover, proteins, possibly including HTT, and miRNA detected in exosomes from a wide variety of sources also highly overlap the HD datasets, suggesting both mechanistic and biomarker links. Finally, the HTT Interactome highly intersects protein networks of pathogenic genes underlying Parkinson's, Alzheimer's and eight non-HD polyglutamine diseases, ALS, and spinal muscular atrophy. These protein networks in turn highly overlap the exosome and homeostatic synaptic plasticity gene sets. Thus, we hypothesize that HTT and other neurodegeneration pathogenic genes form a large interlocking protein network involved in exosome and homeostatic synaptic functions, particularly where the two mechanisms intersect. Mutant pathogenic proteins cause dysfunctions at distinct points in this network, each altering the two mechanisms in specific fashion that contributes to distinct disease pathologies, depending on the gene mutation and the cellular and biological context. This protein network is rich with drug targets, and exosomes may provide disease biomarkers, thus enabling drug discovery. All the curated datasets are made available for other investigators. Elucidating the roles of pathogenic neurodegeneration genes in exosome and homeostatic synaptic functions may provide a unifying framework for the age-dependent, progressive and tissue selective nature of multiple neurodegenerative diseases.

## Introduction

Huntington's disease (HD) is a monogenic, dominantly inherited neurological disorder caused by a CAG expansion in the huntingtin gene (*HTT*), and is one of nine neurodegenerative diseases caused by polyglutamine (polyQ) expansion. HTT is a large protein of 3,144 amino acids, and when the glutamine encoded in exon 1 exceeds 35, it causes a slow-onset, progressive neurodegeneration, with an age of onset that is inversely Q length-dependent (Andrew et al., 1993; Duyao et al., 1993; The Huntington's Disease Collaborative Research Group, 1993; Langbehn et al., 2010). Although HTT is ubiquitously expressed, HD largely manifests in motor, cognitive, and other neurological symptoms (Reiner et al., 2011). The N terminal fragments of mutant HTT (mHTT) is prone to misfolding and aggregation (Scherzinger et al., 1997; Poirier et al., 2002; Thakur et al., 2009), a feature shared with pathogenic proteins implicated in other neurodegenerative diseases. How mHTT ultimately causes HD still isn't clear, but wild type (WT) HTT has been implicated in a wide variety of biological functions on the cellular level, many of which are impacted by the mutation (reviewed in Imarisio et al., 2008; Ross and Tabrizi, 2011; Labbadia and Morimoto, 2013). Aside from tetrabenazine for symptomatic relief, there is currently no effective disease modifying treatment available, although several are under development (Wild and Tabrizi, 2014; Dayalu and Albin, 2015).

Numerous transgenic and knock-in rodent HD models and other experimental platforms utilize mHTT to drive a variety of endpoints presumed to be relevant for disease pathology, such as behavioral phenotypes in whole organisms, cell toxicity, and protein aggregation. However, these are often driven by overexpression of high Q length HTT fragments. In principle, genetic perturbations or small molecules that impact these endpoints are causal for mHTT pathophysiology. However, uncertainties regarding the disease relevance of the HD platforms and mHTT-driven endpoints, and the sheer volume of these perturbation data that make systematic analyses difficult, have stymied progress in elucidating disease mechanisms. Small molecule screening in HD platforms has also not progressed to drug development, leading to a search for alternate and novel assays (reviewed in Bard et al., 2014).

Given the challenges, a systematic analysis of all existing HD perturbation data from comprehensive curation of the HD literature would be desirable if only to settle whether they hold value. We undertook such an effort over several years, creating a comprehensive HD perturbation database (PerturbDB) from a systematic survey of the HD literature of ~12,500 PubMed articles (current to May 2014) to identify the 456 containing perturbation data that meet predefined criteria, from which a total of 1,218 genes were curated into PerturbDB. The database had been made freely available online during its creation, and an analysis of an early version containing 694 genes had been published (Kalathur et al., 2012). The authors highlighted several biological areas from enrichment analysis with GO terms and KEGG Pathways, but did not analyze in detail the heterogeneous data types or uncover novel pathophysiological mechanisms. We analyzed an updated and much larger PerturbDB, complemented by a curated set of proteins associated with HTT (HTT Interactome, 1,619 in total), with a database-enabled methodology of efficient surveying of PubMed for mechanisms linked to the HD datasets, then building specific mechanistic gene sets for enrichment analyses followed by their rank-ordering, and finally formulating testable hypotheses. We hypothesize that exosome biology and homeostatic synaptic plasticity (HmSP, used here instead of HSP to avoid confusion with heat shock protein), not previously linked to each other or to HD, are functionally linked together and are altered in HD. Moreover, the HTT interactome highly intersects protein networks of pathogenic genes underlying Parkinson's Disease (PD), other polyglutamine (PolyQ) diseases, Alzheimer's Disease (AD), Amyotrophic Lateral Sclerosis (ALS), and spinal muscular atrophy (SMA). All the protein networks of these disease genes also link to exosome biology and HmSP, suggesting that an overlapping protein network connecting these pathogenic proteins gives rise to distinct disease pathologies depending on disease and biological context. All of the curated databases utilized are provided in this publication to enable the scientific community to undertake further analysis and experimental verification.

## Methods

### PerturbDB

We systematically and comprehensively curated the PubMed HD literature (query terms: Huntington's, Huntington, huntingtin) through May 2014 (~12,500 PMIDs) to select articles that met the predefined “perturbation” criterion for PerturbDB: that a gene is perturbed, by either genetic or pharmacological means, in an experimental platform that produces one or more mHTT-dependent endpoint. Putatively, genes whose perturbation impacted mHTT-driven endpoints are causally linked to mHTT pathophysiology. Human genes in which SNPs are reported to modify age of onset of HD are also included. As discussed previously (Kalathur et al., 2012), these criteria were originally part of a Target Validation scoring system developed by the CHDI Foundation. Four thousand and fifty-six PubMed articles met this criterion and a total of 1,266 unique genes were curated into PerturbDB (genes found to have no impact were included but filtered out for this analysis). The specific curation attributes, such as experimental platform, mHTT size, nature of perturbation, and endpoints, are shown in Table S1 in Supplementary Datasheet 4. Genes tested in different species were all mapped to orthologous human Entrez gene IDs, and where there was no match (certain mouse immune function genes, and a small number of yeast, fly, and worm genes) they were excluded from the database, as they are not likely to be relevant for human biology. Certain fly and worm genes each mapped to multiple human orthologs that are closely related, and these were all included to represent the original single gene. For small molecule perturbations the gene ID of the most likely drug target was used. The entire PerturbDB table, with 1,839 unique entries and 212 data columns, is provided in Supplementary Datasheet 1. Excluding genes that had no effect, a total of 1,218 genes impacted mHtt-dependent outcomes in at least one HD experimental platform. These genes, and subsets representing the six major experimental platforms within PerturbDB, were analyzed as described in Results and Discussion. We did not analyze other potentially informative avenues, for example with an endpoint of HTT protein level, but leave it to other interested investigators to utilize the dataset. In addition, the PerturbDB genes, tagged with their experimental summaries, are included in a Master Data Table containing all other gene sets used for this publication (Supplementary Datasheet 2).

### HTT interactome

We curated five major proteomics studies for proteins associated with WT or mHTT using yeast two-hybrid (Y2H) or immunoprecipitation-pull-down from cells and brain tissues. The first study used Y2H (Goehler et al., 2004), followed by one that also used Y2H but also Tagged Affinity Purification-Mass Spectrometry on cells and brain extracts (Kaltenbach et al., 2007). A follow-on study extending the ~100 Y2H interactors to 1,207 secondary interactors (Tourette et al., 2014) was published after our primary analysis was completed and is not included. However, 28% of this set is already in our curated HTT Interactome, having come from other sources. Interactomes from different brain regions of BACHD mouse at different ages (Shirasaki et al., 2012), from cytoplasm and membrane-bound brain fractions of the CAG140 mouse (Culver et al., 2012), and differential interactors between normal HTT and mHTT in striatal cell lines (Ratovitski et al., 2012) were curated. Combining these interactome data with miscellaneous published HTT protein-protein interactors (PPI) curated in Ingenuity IPA (QIAGEN, Redwood City, www.qiagen.com/ingenuity) and the HIPPIE database (http://cbdm.mdc-berlin.de/tools/hippie/information.php; HIPPIE includes data from Goehler et al., 2004) result in 1,619 unique genes whose protein products are part of a combined HTT Interactome. This dataset as a whole (and the five constituent subsets) is included in Supplementary Datasheet 2.

### Gene sets

Genes implicated in different biological functions were curated from various sources and are integrated in the master data table in Supplementary Datasheet 2. A set of 3,549 genes involved in various synaptic functions was curated from several publications and databases (Ashburner et al., 2000; Collins et al., 2006; Zhang et al., 2007; Abul-Husn et al., 2009) and is named SynapseDB. A subset of 1,097 genes implicated in postsynaptic neuronal functions (Collins et al., 2006) is also analyzed on its own for comparison with the larger SynapseDB.

Genes implicated in exosome functions, primarily its release from nerve terminals in the *Drosophila* neuromuscular junction (NMJ; Ataman et al., 2008; Korkut et al., 2009, 2013; Fainzilber et al., 2011; Koles et al., 2012; Kerr et al., 2014), but also the multi-vesicular bodies and 14-3-3 proteins (Fraser et al., 2013) in mammalian cells, were curated from PubMed. PPI of each of these exosome functional genes were obtained from HIPPIE and from Ingenuity IPA. These two databases highly overlap each other as both are curated from PubMed, and it should be noted we did not impose any other selection criteria on the curation, as all results from these two sources that can be mapped to human Entrez gene IDs are included.

Proteins identified in high throughput studies of exosomes or extracellular vesicles from cells, fluids and tissues were curated from online databases (Vesiclepedia: http://microvesicles.org/; data downloaded January 2014), as is a set of 755 miRNA (the latter shown in Supplementary Datasheet 3). A list of the top 200 proteins most often found in high throughput studies of exosome from EVpedia (http://student4.postech.ac.kr/evpedia2_xe/xe/), and a set of proteins detected in exosomes from human CSF (Chiasserini et al., 2014) are also included. The Exosome Protein DB therefore contains a total of 4,019 proteins. Genes required for presynaptic HmSP in the *Drosophila* NMJ (reviewed in Frank, 2014), and postsynaptic and other forms of HmSP in mammalian neuronal cultures were curated from PubMed. PPI of these genes were then constructed from HIPPIE and Ingenuity IPA.

It should also be noted that Supplementary Datasheet 2 includes other gene sets of interest to the field, including PPIs of those involved in proteolysis or degradation of HTT and mHTT (e.g., the NUB1 and CUL genes from a screen based on mHTT protein levels; Lu et al., 2013), and for autophagy, lysosome and ubiquitome functions, different aspects of mitochondrial function and biology, and several transcription factors relevant to HD (e.g., p53, HDACs, CBP, NRF2, and PGC-1α).

Protein interactors of genes implicated in neurodegenerative diseases, including PD, polyglutamine diseases (but excluding HD), ALS, SMA, and AD were also constructed in HIPPIE and IPA. The PD set contains five genes (LRRK2, PARK2, PARK7, PINK1, SNCA) for a PPI total of 763. The AD set consists of APP, PSEN1, PSEN2, and MAPT (tau), of which APP contributes the bulk of the PPI of 2,354 (~90% of the set). The PolyQ set contains genes for seven polyglutamine diseases (SCA1-ATXN1; SCA2-ATXN2; SCA3-ATXN3; SCA6-CACNA1A; SCA7-ATXN7; SCA17-TBP; DRPLA-ATN1; SBMA-AR; all as disease-gene symbol pairs) for a total of 1,139 PPI. The ALS set consists of SOD1, TARDBP, and FUS, with 328 total. C9orf72 was not included because only a handful of PPIs are known. The SMA set contains 241 PPI for SMN1/2. All of these gene sets are found in Supplementary Datasheet 2.

### Other data sources

Gene IDs linked to PubMed articles were downloaded using the gene2pubmed tool ftp://ftp.ncbi.nih.gov/gene/DATA/gene2pubmed.gz; and other sources include publically available GO, KEGG, and REACTOME.

### Building and analyzing integrated databases

**Figure 1:**
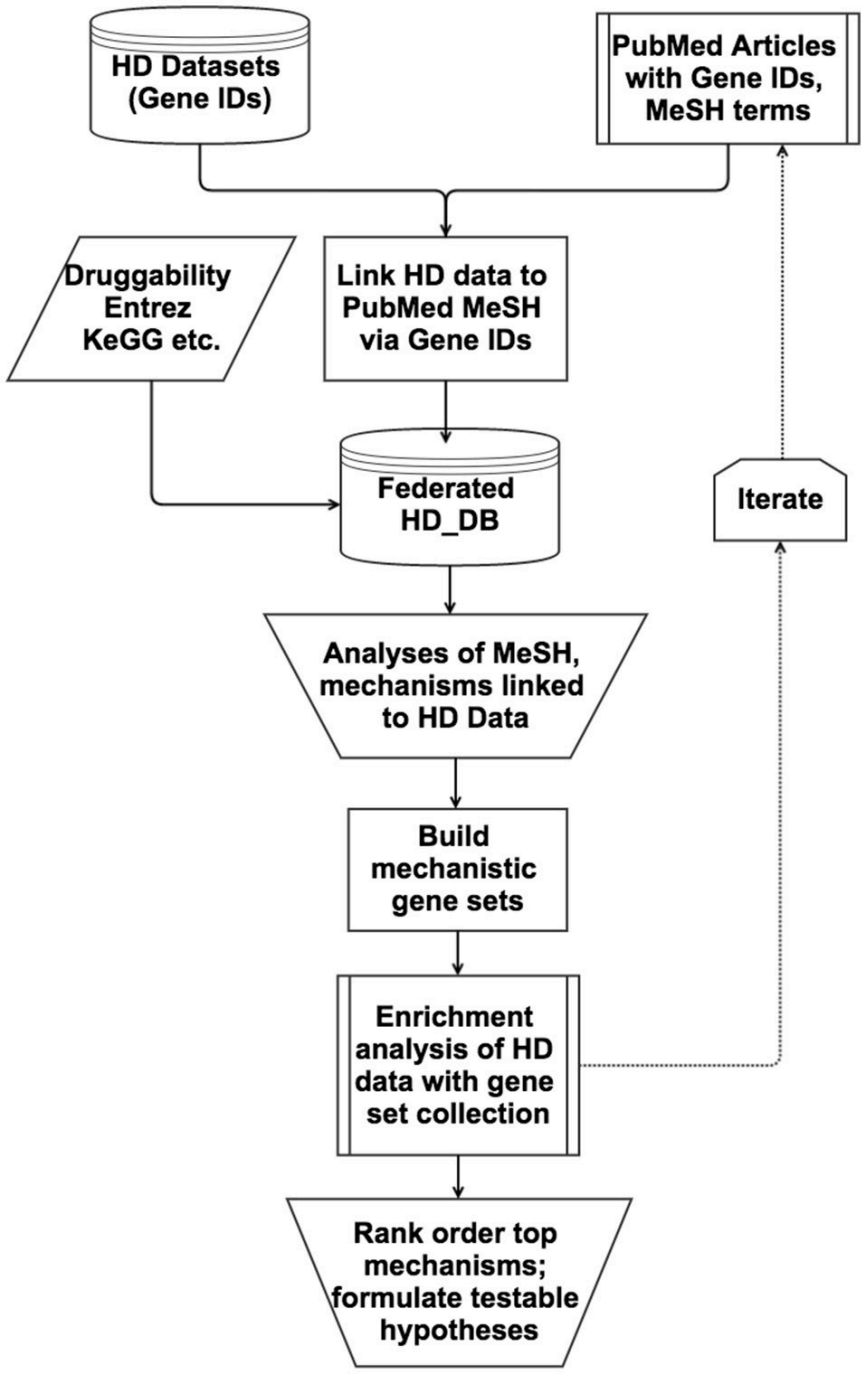
**Flow chart of the database-enabled methodology to link and query data of interest (HD) with literature and other data, followed by enrichment analyses and hypotheses formulation**. The flow chart depicts the multiple streams of structured data that are integrated into the federated HD database (HD_DB) to enable efficient iterative querying of PubMed and enrichment analyses rank order the top mechanisms and formulation of testable hypotheses.

Multiple Access databases were customized for the type of analyses at hand during the workflow depicted in Figure 1. For example, PerturbDB has an Access Form displaying for each gene the experimental platform and attributes such as perturbation and outcome measures (Figure S1), making it easy to query for different combinations of attributes such as those found in two or more platforms (see Supplementary Material Section 1.2 in Supplementary Datasheet 4). After analyses of the PerturbDB data, the HTT Interactome was incorporated to enable analyses of the two datasets, with additional fields added for the HTT Interactome in the Access Form (not shown). To query PubMed articles that co-cite gene IDs and MeSH terms of interest, additional databases incorporated the HD gene IDs, the selected MeSH terms and the number of co-citation articles, with link-outs to the abstracts for the MeSH headings (Figure S2). This allows for efficient surveying of multiple corners of PubMed containing articles co-citing the HD genes (and other gene sets) with particular MeSH headings. The iterative selection of new MeSH terms using this approach then leads to potentially interesting HD mechanisms for which mechanistic gene sets can be built for enrichment analyses and rank-ordering.

### Statistical analysis

Statistical significance of gene set overlaps was tested using Fisher's exact test. For each round of testing we used as reference set a large collection of ~2,000 gene sets that include all genes from public sources (GO, KEGG etc.) and all custom-curated gene sets, representing a wide diversity of biological functions. We used Benjamini-Hochberg FDR estimates, derived independently for each query set from the *p-*values of its overlaps with all reference sets, to correct for the large number of statistical tests. Since all of our query gene sets were compiled from the literature, we cannot *a priori* exclude the possibility that some of the query and reference gene sets are not independent; hence, reported *p-*values should be treated as descriptive rather than inferential. We rank-order the results and generally only examine the most highly ranked top gene sets for mechanisms and biology of interest. To make our results more reader-friendly, we report enrichment significance as −log_10_ FDR, denoted −log FDR. We use an arbitrary minimum cutoff of −log FDR value of 10, because in almost all cases the top ranking gene sets show values much higher than 10, often into the 100s. Thus, we rely more on the relative rank order and degree of overlap between datasets than the absolute values.

Area proportional Venn diagrams were plotted using the eulerAPE program using ellipses (Micallef and Rodgers, 2014).

## Results and discussion

### Overview of the database-enabled methodology

Our overall strategy on analyzing large datasets is to first systematically curate them into databases for initial analysis to determine their usefulness for further work, then to integrate them into databases with iteratively selected PubMed literature to produce mechanistic gene sets for enrichment analyses and rank ordering, as outlined in Figure 1 and detailed in Methods Section Building and Analyzing Integrated Databases. Applying this workflow, we analyzed PerturbDB (genes putatively linked to mHTT pathophysiology) and the HTT Interactome (proteins in complex with HTT and presumed to be involved in HTT biology), and concluded that they qualified as foundational HD datasets (summarized in Section Overview of the HD Datasets and detailed in Supplementary Material Section 1 in Supplementary Datasheet 4). We then curated PubMed articles co-citing the HD gene IDs and MeSH terms of interest and manually surveyed them for potential HD mechanisms. Done in the far more efficient database format described in Methods Section Building and Analyzing Integrated Databases, we iteratively surveyed the literature with progressively focused MeSH terms to custom-build specific mechanistic gene sets that were incorporated into an ever-growing gene set collection for enrichment analysis. Rank ordering of the top mechanisms then lead to in-depth study and hypotheses formulation, as discussed below. This methodology is particularly useful for large datasets as it facilitates surveying large and often-unfamiliar swaths of PubMed, combining the efficiency of automated database federation with insights from manual perusal of the integrated literature. Future implementation of this methodology could include natural language processing tools to improve the efficiency and accuracy of literature curation associated with identifiers for genes or chemicals. Deploying this methodology, we settled on a final database (master data table in Supplementary Datasheet 2) containing gene sets of exosome biology and HmSP as the top ranking mechanisms from enrichment analyses of datasets from HD and other neurodegenerative diseases.

### Overview of the HD datasets

#### The two HD datasets highly intersect each other and are worthy of further analysis

The detailed analysis of the two comprehensively curated HD datasets, PerturbDB (1,218 genes) and the HTT Interactome (1,619 genes), is presented in Supplementary Material (Section 1 in Supplementary Datasheet 4), and we only summarize the main findings here. The validity of the causal relationship to mHTT pathology of the genes in PerturbDB, with their disparate cellular context and mHTT-driven endpoints, has long been debated. However, the major PerturbDB experimental platforms, in particular those of *Drosophila in vivo* phenotypes, mammalian mHTT aggregation, and cell culture toxicity, highly overlap each other despite their very different biological context (Supplementary Section 1.2; Table S2A in Supplementary Datasheet 4). Thus, there are likely common mechanisms mediating mHTT-induced dysfunctions despite the disparate cellular context and endpoints. Moreover, PerturbDB highly overlaps the HTT Interactome, at ~30% between the two (−log FDR ~131), and again more so for the three major experimental platforms (Tables S2A,B; Figures S3A,B in Supplementary Datasheet 4). As the HTT Interactome by and large represents normal functions of HTT (only a relatively small number of HTT interactors are specific for either mHTT or HTT), the high number of HTT interactors that can perturb mHTT endpoints suggests that mHTT can both drive gain-of-function pathologies and disrupt normal biology of HTT. The comprehensive nature of the HTT Interactome and its origin from multiple sources also make it more likely to fill biological gaps not studied in the more hypotheses-driven PerturbDB datasets. Thus, we conclude that these two HD datasets are suitable as foundational data with which to apply our database-enabled methodology and workflow to formulate hypotheses on mHTT pathogenic mechanisms, as shown in Figure 1.

### Exosome biology and HD

#### Exosomes provide novel intercellular communication system for synaptic functions

As described in Supplementary Material Section 2 in Supplementary Datasheet 4, we first curated a large set of genes possessing neuronal and synaptic functions (SynapseDB) and found highly significant enrichment of the HD datasets (Table S3, Figure S4 in Supplementary Datasheet 4), not surprising for a neurodegenerative disease. We then found that a small set of genes critical for synaptic vesicle functions are found in the HD datasets (Table S4 in Supplementary Datasheet 4). While examining these genes, we encountered an interesting set of studies on exosomes (or more broadly, extracellular vesicle or EV) in neuronal functions in which many of these synaptic genes are implicated (Ataman et al., 2008; Korkut et al., 2009, 2013; Fainzilber et al., 2011; Koles et al., 2012; Kerr et al., 2014). Exosomes are 50–100 nm extracellular vesicles released and taken up by all cells both for intercellular communication and for disposal of cellular material (reviewed in Pant et al., 2012; Kalani et al., 2014). Exosome biology is only beginning to be studied intensively in the nervous system, both as a novel means of intercellular communication and as a pathogenic mechanism in neurodegeneration (Chivet et al., 2013; Costanzo and Zurzolo, 2013; Schneider and Simons, 2013; Rajendran et al., 2014; Tsilioni et al., 2014; Coleman and Hill, 2015). The “transmissibility” of misfolded and aggregated forms of pathogenic proteins for multiple neurodegenerative disorders, as has been shown for prions, together with possible involvement of exosome-like secreted vesicles, have generated a great deal of interest. Thus, the detection of α-synuclein, LRRK2, Aβ and tau, prions, SOD1, and TDP-43 in exosomes suggests one means by which these pathogenic proteins could be propagated in Parkinson's (PD), Alzheimer's (AD), Creutzfeldt–Jakob disease, and ALS. On the other hand, exosomes may also ameliorate the disruption of synaptic plasticity by Aβ via sequestration on surface proteins (An et al., 2013). Thus far no direct link between exosome and mHTT has been reported, although transfer of polyglutamine aggregates between neurons via “tunneling nanotubes” (Costanzo et al., 2013), and transneuronal propagation of mHTT that depends on the vesicular fusion machinery (Pecho-Vrieseling et al., 2014), and transfer between neurons and glia (Pearce et al., 2015) have been reported. Moreover, forward genetics studies in *Drosophila* larval NMJ showed that wnt signaling, in a bidirectional manner, mediates synaptic structural changes and a particular form of plasticity in response to spaced intensive interval stimulation (Ataman et al., 2008; Korkut et al., 2009, 2013; Fainzilber et al., 2011; Koles et al., 2012; Kerr et al., 2014). This NMJ synaptic plasticity requires coordination between presynaptic motor neuron and postsynaptic muscle cell in both anterograde and retrograde directions, whereby alteration in presynaptic neurotransmitter release is tightly coupled to the number of postsynaptic neurotransmitter receptors. This coordination is in turn mediated by release of exosomes bearing wnt binding partners from the motor neuron terminal and their uptake by the postsynaptic muscle fiber. Thus, in addition to the well-characterized inter-neuronal and synaptic communication systems, exosomes provide yet another level of control on a form of *Drosophila* NMJ synaptic plasticity. The importance of exosomes in well-characterized forms of synaptic plasticity and whether they contribute to synaptic deficits in neurodegeneration, however, is unclear.

#### Genes required for exosome functions and their protein-protein networks are enriched in the HD datasets

The *Drosophila* studies identified eight genes critical for the release and two genes for the uptake of exosomes at the NMJ. We mapped these 10 genes to 13 human orthologs, adding family members for ADP-ribosylation factor, vacuolar proton pump ATPases, and dynamin due to the presence of these related mammalian orthologs in HD datasets. These genes are shown in Table 1, together with 14-3-3 proteins and the ESCRT complexes involved in the life cycle of exosomes (Colombo et al., 2013; Fraser et al., 2013), and their relationships to the HD datasets. Strikingly, all the mammalian orthologs of the *Drosophila* genes are also found in PerturbDB and/or the HTT Interactome (Table 1, rows 1–13). These are synaptic vesicle fusion and endocytosis genes (syntaxin 1A, RAB11, dynamin, and clathrin); vacuolar proton pump ATPases for organelle acidification; ADP-ribosylation factor; Myosin V; 14-3-3 proteins required for the release of exosomes containing LRRK2, a pathogenic PD gene (Fraser et al., 2013); and the ESCRT complexes required for endosomal trafficking through the multi-vesicular bodies (MVBs). Indeed, *ALL* 27 genes for exosome function in the neuronal context thus far have also been independently implicated as modifiers of mHTT-driven endpoints and/or as part of the HTT Interactome, with 21 out of the 27 in the former, 19 in the latter, and 13 found in both (Table 1). This strong concordance indicates a close relationship between exosome and HTT biology and mHTT pathology. Interestingly, the overlap with PerturbDB is almost exclusively in *Drosophila in vivo* phenotypes and the protein aggregation platforms (Table 1), two of three that stand out as most highly intersecting with other PerturbDB platforms and with the HTT Interactome. The 11 *Drosophila* genes are in synaptic vesicle biology or are members of the 14-3-3 and ESCRT families, and their concordance in larval NMJ exosome function and pan-neuronal mHTT phenotypes in adult brain implicates exosome biology in the complex behavioral phenotypes of mHTT. The overlap with the aggregation platforms, on the other hand, implicates protein metabolism and processing in exosome biology in the context of mHTT, perhaps due to the dual roles of exosome in protein cargo delivery/disposal and in intercellular communication.

**Table 1:** **Overlap of Exosome Functional Gene Set with PerturbDB and HTT Interactome**.

	**Exosome function**	***Dme* gene**	**Gene symbol**	**Gene name**	**PerturbDB**	**HTT Interactome**
1	Release	*Arf79f*	ARF1|3	ADP-ribosylation factor class I		Yes
2			ARF4|5	ADP-ribosylation factor class II	Aggreg	
3	Release	*Vha68-2*	ATP6V1A	ATPase, H+ transporting	Aggreg	Yes
4	Release	*Vha55*	ATP6V1B2	ATPase, H+ transporting		Yes
5			ATP6V1B1	ATPase, H+ transporting	Aggreg	
6	Release	*Vha26*	ATP6V1E1	ATPase, H+ transporting	Aggreg	Yes
7	Release	*VhaSFD*	ATP6V1H	ATPase, H+ transporting	Aggreg	Yes
8	Release	*Myo5*	MYO5A|B	Myosin V		Yes
9	Release	*Rab11*	RAB11A|B	Rab 11	HD Fly; Aggreg	Yes
10	Release	*Syx1a*	STX1A	Syntaxin 1A	HD Fly	Yes
11	Uptake	*Chc*	CLTC	Clathrin, heavy chain	HD Fly	Yes
12	Uptake	*Shi*	DNM1	Dynamin 1	HD Fly	Yes
13			DNM2	Dynamin 2	Aggreg; Cell Tox	Yes
14	Release		YWHAB	14-3-3 family	HD Fly	Yes
15			YWHAE	14-3-3 family	HD Fly; Aggreg	Yes
16			YWHAZ	14-3-3 family	HD Fly; Aggreg	Yes
17			YWHAG	14-3-3 family	HD Fly	Yes
18			YWHAH	14-3-3 family		Yes
19			YWHAQ	14-3-3 family		Yes
20	MVBs		VPS28	ESCRT-I Complex	HD Fly	
21			VPS25	ESCRT-II Complex	HD Fly	
22			CHMP3	ESCRT-III Complex	Aggreg	
23			CHMP4A	ESCRT-III Complex	Aggreg	
24			CHMP4B	ESCRT-III Complex	HD Fly; Aggreg	Yes
25			CHMP5	ESCRT-IV Complex		Yes
26			VPS4A	ESCRT-IV Complex	Aggreg; Cell Tox	
27			VPS4B	ESCRT-IV Complex	Aggreg	

*Aggreg, mammalian mHTT or C. elegans in vivo pure polyQ aggregation; HD Fly, Drosophila in vivo phenotypes platform; Cell Tox, Mammalian cell culture toxicity platform; MVBs, multi-vesicular bodies*.

To perform robust enrichment analyses and to widen the gene sets to test for specific mechanisms involving exosomes, we curated and combined PPIs for each of the mammalian exosome functional genes to produce a protein network important for exosome release and uptake. This Exosome Synaptic PPI set of 892 genes would be analogous to the HTT Interactome and therefore suitable for enrichment analysis (see Supplementary Datasheet 2 for the gene sets). The 14-3-3 genes and ESCRT complex members were not included as they form very large protein interaction networks on their own and are probably best analyzed separately (PPIs of these two families are nevertheless included in Supplementary Datasheet 2). We summarize here the main findings for the two HD datasets, while details are found in Supplementary Material (Section 3 in Supplementary Datasheet S4).

The Exosome Synaptic PPI is highly enriched in the HTT Interactome and PerturbDB, overlapping ~18% of each of them, and even more so the HD Common set of genes at 33% with high –log FDR values (Table 2). Thus, genes found in both HD datasets shows a higher relative percentage of overlap compared to either HD dataset alone. That the protein network of genes critical for exosome synaptic functions highly intersects the HTT Interactome further supports a link between exosome and HTT biology. The overlap with PerturbDB (the *Drosophila in vivo* phenotypes and mammalian mHTT aggregation are again the two highest overlapping, Table S5 in Supplementary Datasheet 4) in turn suggests that certain mHTT dysfunctions may involve exosome biology. Thus, these results strengthen and extend the hypothesis that synaptic and neuronal functions of exosomes are linked to HTT and mHTT. This hypothesis can be experimentally verified in the *Drosophila* NMJ and then extended to mammalian experimental platforms, and followed by elucidating therapeutic opportunities in exosome function linked to mHTT pathophysiology.

**Table 2:** **Overlap of HD with exosome datasets**.

	**Exosome Synaptic Rel_892**		**Exosome ProteinDB_4019**	
	***n* common**	**FDR**	***n* common**	**FDR**
HTT Interactome_1619	295	2.1E-112	832	2.4E-228
PerturbDB_1218	203	1.5E-62	512	2.9E-92
HD Common_357	117	5.9E-70	222	2.7E-79

#### Proteins and miRNA detected in exosomes are highly enriched in HD datasets

The contents of exosomes or extracellular vesicles have been profiled in a variety of cells and tissues, and these data are aggregated in public databases (see Section Methods for sources). We curated a set of proteins (Exosome ProteinDB, with 4,019 genes) and miRNA (755 in total) detected in exosomes from a variety of tissues and cells for enrichment analysis. Almost all of the tissues and cells reported are non-neuronal, with the exception of a set of 739 proteins detected in human CSF exosomes (Chiasserini et al., 2014). A detailed analysis of the intersection of this dataset with the HD data subsets is presented in Supplementary Material Section 3 in Supplementary Datasheet S4.

**Figure 2:**
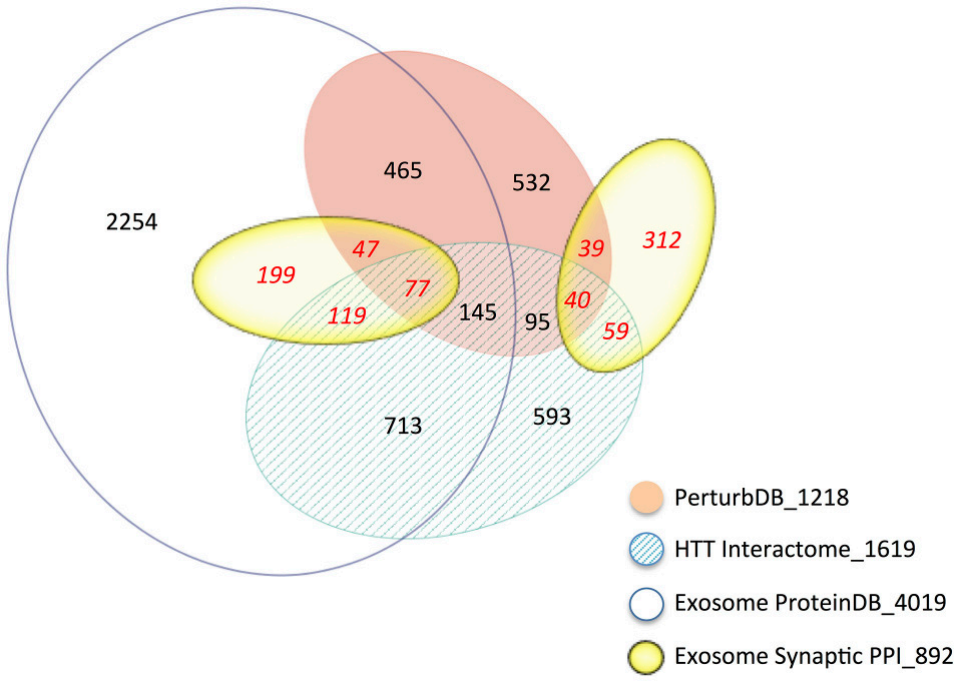
**Venn diagram of the intersections of the Exosome and HD datasets**. The area of the sets is approximately proportional to the set size, with the number of genes indicated. The number following the underscore indicates the total size of each dataset.

Despite the relative paucity of data from brain and neuronal tissues, the Exosome ProteinDB still highly intersects the HTT Interactome (51% of the latter, −log FDR ~228; Table 2), PerturbDB (42%, −log FDR ~92) and in particular the HD Common set (62%, −log FDR ~79). Half of the Exosome Synaptic PPI set (442 genes) is found in the Exosome ProteinDB, and both highly overlap the HD datasets (see Venn diagram in Figure 2). Indeed, only 35% of the Exosome Synaptic PPI set is not intersected. Thus, many HTT associated proteins may be either exosomal components or signaling molecules, and contribute to mHTT pathophysiology by altering exosome functions. Also noteworthy is that HTT itself is detected as protein in exosomes or extracellular vesicles from human B cells (Meckes et al., 2013), thymic tissue (Skogberg et al., 2013) and urine (Wang et al., 2012; Fraser et al., 2013), and as mRNA in exosomes from cancer cells (Skog et al., 2008; Hong et al., 2009), raising the interesting question as to whether mHTT might also be detected in exosomes in HD patients. If true, then HD joins the other neurodegenerative diseases where transmission of misfolded pathogenic proteins between cells may play an important role in disease pathology, and that the exosome content in HD patients should be studied as potential biomarkers for disease state and treatment status.

miRNA and their target genes have been implicated in neuronal functions and in neurodegenerative diseases (Higa et al., 2014). We curated two studies of differentially expressed miRNAs in HD: in striatum of two mouse HD models (Lee et al., 2011); and in human HD patient brain frontal cortex and caudate (Martí et al., 2010; Supplementary Datasheet 3) and found that 50–60% of differentially expressed miRNAs in HD are also found in exosomes (Table 3). Other miRNAs linked to HD, such as miR-22, a “perturbation” molecule due to its neuroprotective properties in an HD cell culture platform (Jovicic et al., 2013), and miR-214, miR-150, miR-146a, and miR-125b that target the Htt gene (Sinha et al., 2011), are all found in exosomes. Moreover, 7 of 11 miRNAs dysregulated in the frontal cortex of HD monkeys are found in exosomes (Supplementary Datasheet 3), including the miRNA-128a that targets HTT and HIP1 and is also downregulated in the brains of pre- and post-symptomatic HD patients (Kocerha et al., 2014). Finally, miR-26 in astrocyte-derived exosomes is involved in CNS diseases and synaptic plasticity (Lafourcade et al., 2016), and is also differentially expressed in HD. Conversely, neuronally released exosomes can regulate expression of the glutamate transporter GLT1 in astrocytes (Morel et al., 2013), and GLT1 is downregulated in HD, leading to excess glutamate and deficient ascorbate in the synapses that may contribute to HD (Miller et al., 2008, 2012; Huang et al., 2010). The intriguing roles of exosomes in glial-neuronal interaction and their potential involvement in HD are further discussed in Supplementary Material Section 4 in Supplementary Datasheet 4. Thus, both the proteins and miRNAs in exosomes are linked to HD and further support the importance of exosome biology for HD.

**Table 3:** **Overlap of miRNA in exosome (755) with miRNA changed in HD brains**.

	**miRNA changed**	***n* common**	**% miRNA changed**
Mouse R6/2_293	80	49	61
Mouse YAC128_169	79	52	66
Human HD Cortex	158	79	50
Human HD Caudate	131	66	50

#### Testing the hypothesis that exosome dysfunction caused by mHTT plays a role in HD

In conclusion, the putative roles of HTT as a scaffolding protein, its high intersection with synaptic proteins, its role in regulating vesicular organelle movement, trafficking, and transport (Zala et al., 2008, 2013; Roux et al., 2012; Liot et al., 2013; Fu and Holzbaur, 2014), and it being part of the presynaptic cytomatrix (Yao et al., 2014), are all possibly linked to various aspects of exosome biology. The impact of mHTT could occur anywhere along the life cycle of an exosome, from its biogenesis during endosome formation and maturation, processing and transport through the MVB, release from nerve terminals and other regions of neurons and likely other cell types, and ultimately uptake by postsynaptic neurons or by glia cells. The impact of mHTT could also be cell and tissue specific, depending on the roles of exosomes in those contexts. Many questions of exosome synaptic biology are addressable in the *Drosophila* NMJ platform, and these will drive the critical experiments to translate and validate in mammalian neuronal culture and other platforms, where exosome biology is less well-studied. The abundance of potential drug targets involved in exosome biology and the ability of exosomes to carry disease biomarkers also provide a promising path forward for HD drug discovery. A key question, however, is which functions mediated by exosomes are impacted by mHTT and contributes to disease.

### Homeostatic synaptic plasticity in HD

#### Homeostatic synaptic plasticity is implicated in multiple CNS disorders

The critical role of exosome in a form of synaptic plasticity at the *Drosophila* NMJ suggests a broader link to neuronal plasticity, but this has been little studied. Whether and how the impact of mHTT on synaptic activity drive disease pathology is also unclear, as many studies HD models have reported variable alterations in neuronal and synaptic activity in different experimental platforms, although there is a focus on the corticostriatal connections that are most vulnerable in HD (reviewed in Raymond et al., 2011; Sepers and Raymond, 2014). The effects of mHTT, however, are likely countered by compensatory mechanisms until clinical manifestation due to the slow and progressive nature of HD. In this context, a key synaptic regulatory mechanism, homeostatic synaptic plasticity (HmSP), is particularly intriguing but has been little studied in HD. Homeostasis is a fundamental feature of biological systems, and HmSP is a form of “rheostat” control allowing the nervous system to regulate repeated perturbations such as episodes of synaptic plasticity within an optimal biological range (Lee et al., 2014). Distinguishing features of HmSP include the multiple levels it operates in, from synapses to cells to networks, the slow time course of its induction and maintenance, and the likelihood of a wide range of tolerated operational set points (some have argued that “allostasis” is a more appropriate term and concept Sterling, 2012). There are multiple forms of HmSP, including synaptic downscaling and upscaling, in which chronic activation or inhibition lead to compensatory decrease or increase in postsynaptic responsiveness, respectively, often mediated by availability of postsynaptic AMPA receptors (Turrigiano, 1999, 2007, 2012; Siddoway et al., 2014). Notably, mHTT impacts AMPA receptor trafficking and impairs glutamatergic neurotransmission (Mandal et al., 2011). Presynaptic HmSP is mediated by compensatory changes in transmitter release due to chronic postsynaptic inhibition or activation, and involves retrograde signaling and presynaptic transcriptional alterations (Davis, 2013). Glia also regulate synaptic upscaling via TNF-α signaling by altering the balance of synaptic AMPA and GABA receptors (Pribiag and Stellwagen, 2013, 2014). HmSP is drawing increasing interest as a fundamental mechanism critical for neuronal functions, especially other forms of synaptic plasticity. HmSP is particularly attractive as a neurodegeneration mechanism because of its highly adaptable and context-dependent nature operating over a long time frame. Thus, while HmSP may compensate for mHTT synaptic deficits (as suggested in Rocher et al., 2016), dysfunctions in HmSP itself over time may also play pathogenic roles in HD.

A growing list of genes has been shown to play critical roles in various forms of HmSP, and some are known to also play key pathogenic roles in a number of CNS disorders. For example, polyglutamine expansion in the Cav2.1 subunit CACNA1A (OMIM ^*^601011) of voltage-gated calcium channels (VGCCs), the mammalian ortholog of the *Drosophila* gene *cacophony* that is required for presynaptic HmSP (reviewed in Frank, 2014), causes the autosomal dominant spinocerebellar ataxia type 6 (SCA6, OMIM 183086), one of nine polyQ diseases that include HD. Other mutations in this gene are associated with Episodic ataxia, type 2 (OMIM 108500). Retrograde signaling in HmSP engages transcription factors that include SMAD and PAX members, and SMN1 (OMIM 600354), mutation of which causes SMA, another monogenic neurodegenerative disease. These transcriptional regulators at least partly modulate expression of potassium channels of the Kv1 and Kv4 families that together with VGCCs regulate the readily releasable pool of transmitters (Lazarevic et al., 2013; Frank, 2014). Mutations in KCNA1 (Kv1.1) cause Episodic Ataxia, Type 1 (OMIM 160120), and those in KCND3 (Kv4.3) cause the very rare Spinocerebellar Ataxia 19 (SCA19; OMIM 607346). Presynaptic HmSP signaling also includes the BLOC-1 and SNARE complexes at the active zone. Dysbindin (DTNBP1, OMIM 607145), a member of the BLOC-1 complex, has been implicated in schizophrenia. Finally, two genes required for postsynaptic HmSP are implicated in CNS disorders, MECP2 (OMIM 300005) in Rett Syndrome and FMRI (OMIM 309550; Chen et al., 2014) in the Fragile X Syndrome and Fragile X-associated tremor/ataxia. MECP2 deficiency alters HTT/HAP1 dependent axonal transport of BDNF and of APP (Roux et al., 2012). Thus, mutations of genes all along the pathway for HmSP lead to multiple CNS and neurodegenerative disorders, supporting the importance of this mechanism for normal functioning of the nervous system, and points to its potential significance in HD.

#### Genes required for HmSP and their protein networks highly overlap both the HD and exosome datasets

**Figure 3:**
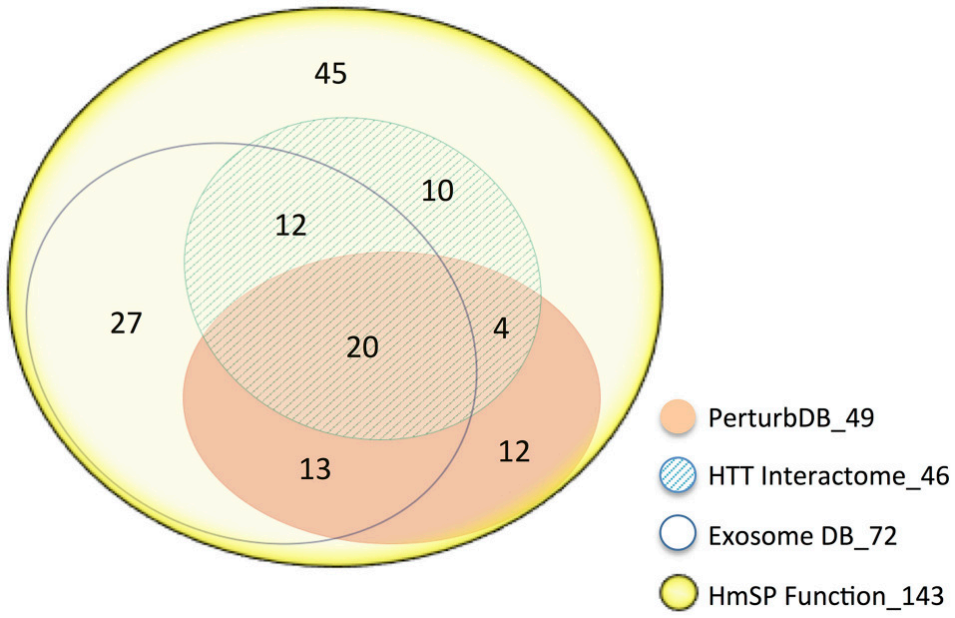
**Venn diagram of the 143 HmSP perturbation genes and their distribution amongst the HD and Exosome datasets**. The area of the sets is approximately proportional to the set size, with the number of genes indicated. The number following the underscore indicates the total size of each dataset.

We curated the HmSP literature to produce a comprehensive list of 143 genes that are critical for the proper functioning of different forms of HmSP to perform enrichment analysis (see Supplementary Datasheet 2). These 143 genes are highly interconnected between themselves with only a handful unconnected (queried in Ingenuity IPA, results not shown). This HmSP Functional gene set highly intersects the HTT Interactome and PerturbDB, with 71 genes or 50% found in one or both of the HD datasets, and the relative % overlap again higher in the HD Common set (Table 4). Many of the 49 PerturbDB genes overlapping the HmSP set are highly connected nodal signaling genes (e.g., BDNF, mTOR, CAMKII, AKT, CREB1) and are as well-known in the HD as they are in neuronal functions and cell survival, suggesting HmSP as a novel mechanism. Of the 143 HmSP genes, 72 or 50% are found in the Exosome DB (which combines the Exosome Synaptic PPI and Exosome ProteinDB), linking exosome biology to HmSP. Of these 72 genes, 45 are found in either or both HD datasets. Finally, 20 genes are common in HmSP, exosome, and HD datasets. Ninety-eight of 143 HmSP genes are also found in the HD and/or exosome datasets, with only 45 not present in these datasets (Figure 3). This further suggests that exosome biology and HmSP are each linked not only to HTT function and mHTT pathogenesis but also to each other, and where they intersect may be important for HD.

**Table 4:** **Overlap of HD datasets with HmSP Functional genes**.

	**HmSP Functional Set_143**		
	***n* common**	**FDR**	**% HD Set**
HTT Interactome_1619	46	8.2E-18	3
PerturbDB_1218	49	2.4E-25	4
HD Common_357	24	9.6E-19	7

Continuing with our strategy of enrichment analysis of protein networks built on key mechanistic genes, we curated PPI sets for each of 65 known for their roles in mediating eight specific forms of HmSP (Table S6 in Supplementary Datasheet 4), then combining the PPI sets for each form of HmSP: (1) presynaptic HmSP with Eph signaling to calcium channels (VGCCs) and regulation of the readily releasable pools of transmitters; (2) retrograde signaling to presynaptic neuron; (3) transcriptional regulation in presynaptic neurons; (4) postsynaptic synaptic up-scaling and (5) down-scaling; (6) glial TNFα-mediated up-scaling; (7) retinoic acid- and the Fragile X gene FMR1 mediated up-scaling; and finally (8) calcium signaling via CAMKII and debrin for HmSP. The eight PPI sets show high degree of intersection with each other, with 50% or more of genes in each set in common with at least one other set (Table S7 in Supplementary Datasheet 4), indicative of multiple overlapping protein networks that are tightly controlled to coordinate the critical neuronal modulatory functions of the specific forms of HmSP. We then combined these eight sets into an HmSP database (HmSP DB) of 3,782 unique genes, and the enrichment analysis for this set is summarized here. The detailed analysis is found in Supplementary Material Section 5 in Supplementary Datasheet 4.

**Figure 4:**
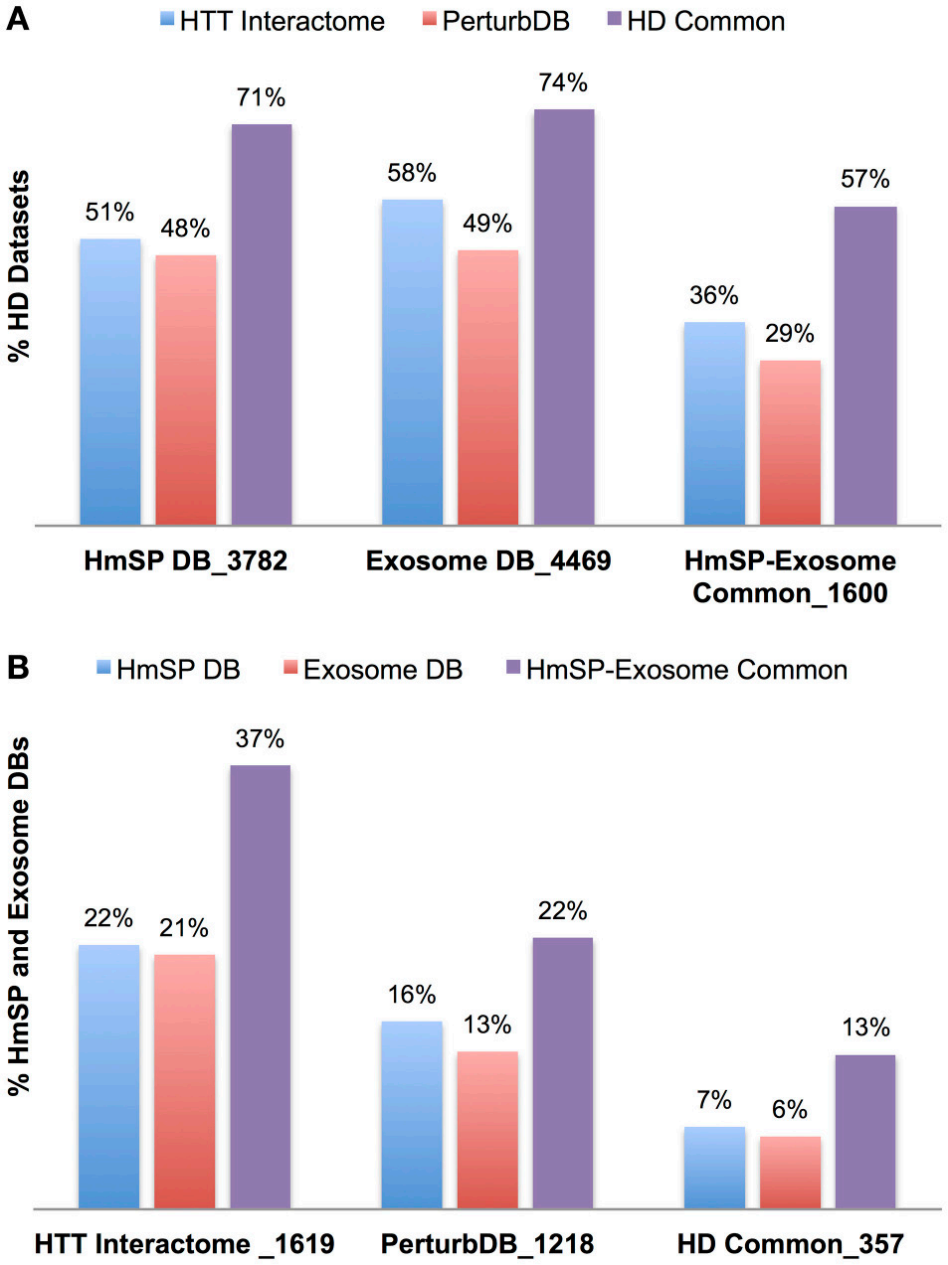
**Relative percent overlap of the HD datasets with the HmSP and Exosome DB, and the converse. (A)** Percentage of the HTT Interactome, PerturbDB and HD Common (found in both sets) that overlap with the HmSP DB, Exosome DB, and the HmSP-Exosome Common (found in both DBs). **(B)** Percentage of the HmSP DB, Exosome DB, and the HmSP-Exosome Common that overlap with each of the HD dataset is shown. The number following the underscore indicates the size of each dataset.

As expected, this larger representation of the HmSP functional set shows highly significant overlap with the HD datasets, at 51% of the HTT Interactome and 48% of PerturbDB, and again a higher 71% of HD Common (Table 5, Figure 4A). A similar pattern of overlap with HD datasets holds true for the combined Exosome DB and the 1,600 genes found in both HmSP and the Exosome DBs. Again, the top two PerturbDB platforms that overlap HmSP are the *Drosophila in vivo* phenotypes and mHTT aggregation platforms (Table S8 in Supplementary Datasheet 4). Conversely, for each of the HD datasets there is also an increase in the relative % overlap of the HmSP-Exosome Common set vs. the HmSP or Exosome DB alone (Figure 4B). A Venn diagram of the HD datasets and HmSP and Exosome DBs (Figure S5) further supports their close tripartite relationships. A similar pattern is observed for the PPI of each of the specific form of HmSP and their common genes with the Exosome DB (Supplementary Material Section 5, Tables S9A,B in Supplementary Datasheet 4). Notably, there are a multitude of known drug targets in these gene sets, many of which have been studied in the context of HD but not with HmSP or exosome biology as endpoints, and others that have not yet been implicated in HD. Notable are CDK5 and calcium channels at the presynaptic level, PLK2 and others at the postsynaptic dendrite, and β-catenin in both glial and synaptic compartments, all highly connected in networks with multiple druggable entry points that provide therapeutic opportunities once the link to the disease is elucidated.

**Table 5:** **Overlap of HD datasets with HmSP and exosome DBs**.

	**HmSP DB_3782**		**Exosome DB_4469**		**HmSP-Exosome Common_1600**	
	***n* common**	**FDR**	***n* common**	**FDR**	***n* common**	**FDR**
HTT Interactome_1619	825	5.6E-243	931	4.8E-270	586	1.3E-287
PerturbDB_1218	583	1.7E-151	593	9.2E-120	356	3.3E-132
HD Common_357	254	1.9E-117	265	1.8E-108	202	4.3E-139

#### Testing the hypothesis: exosome is involved in HmSP, and both are dysfunctional in HD

The hypothesis that exosomes and HmSP are functionally linked and collectively impacted by mHTT can be tested in the well-characterized *in vivo Drosophila* and mammalian cell culture platforms. *Drosophila* larval NMJ is best suited to test whether presynaptic HmSP and exosome synaptic functions are linked, then each mechanism and where they intersect can be tested for the impact of expressing mHTT in motor neurons, glia or perhaps even the postsynaptic muscle cells. When the mHTT effects are characterized, then known HD modifiers can be tested for their impact on these endpoints. A concordance of modifiers between the mHTT-driven exosome and HmSP endpoints in the NMJ vs. behavioral phenotypes in the CNS would further support the hypothesis. Postsynaptic HmSP such as upscaling and downscaling are best studied in neuronal cell culture, and experimental verification of the impact of mHTT on postsynaptic HmSP is straightforward. Moreover, the role of exosomes in postsynaptic HmSP will require further detailed study before the question of mHTT's impact can be addressed. Success will lead to the development of new HD assays to test the effects of genetic and pharmacological perturbations using these novel mHTT readouts.

An important feature of HD that we have yet to address is its inverse Q length dependency of the age-of-onset of motoric symptoms. The vast majority of PerturbDB readouts are driven by overexpressed mHTT generally of over 100 Qs. Lower Q lengths of most clinical cases (>40 Q) do not induce robust readouts. For example, HD patient fibroblasts with 49 and 70 Qs show a delay in receptor recycling to plasma membrane via clathrin-coated vesicles because of aberrant Rab11 activity (Li et al., 2009) and other downstream effects (Li et al., 2010, 2012), revealing a subtle deficiency in post-endocytic membrane recycling in HD. Similarly, we predict that the impact of lower Q length mHTT on exosome biology and HmSP are subtle and difficult to detect unless additional stressors push the system outside of its compensatory boundaries. We propose that HmSP not only compensates for the effects of mHTT throughout life, but that mHTT actually alters HmSP together with exosome functions, so that eventually the dysfunction in homeostatic mechanisms, perhaps in concert with aging, as the phenotypic consequences of the HTT mutation become progressively more apparent.

In conclusion, we hypothesize that exosomes are involved in certain aspects of HmSP, similar to its critical role in fly NMJ synaptic plasticity, and that WT HTT is involved in both. This tripartite functional link is then altered by mHTT, which eventually result in HD. Experimental verification of the mHTT effects on exosome and HmSP functions in the NMJ and neuronal cell culture platforms will enable further research of both these mechanisms in HD and aid the hunt for drug targets. This hypothesis does predict that WT HTT is involved in exosome biology and HmSP, conceivably linked to the many roles ascribed to HTT, such as transport and trafficking (Caviston and Holzbaur, 2009), ER stress (Vidal et al., 2011), and transcription (Moumné et al., 2013). Finally, our hypothesis may also be relevant for the HTT lowering strategies currently in clinical development that do not discriminate the WT from the mutant form of HTT, as it is plausible that lowering both forms of HTT too drastically may negatively impact the normal functioning of exosome and HmSP and possibly confound any positive effects due to the lowering of mHTT itself.

### Protein networks of other neurodegenerative disease genes overlap the HD, exosome, and HmSP datasets

#### Curation of protein networks of neurodegenerative disease genes

We examined whether protein networks of pathogenic genes for neurodegenerative diseases that share with HD the slow onset, progressive deterioration, and regional selectivity of pathologies are also linked to HmSP and exosome biology, particularly as many HmSP genes are already implicated in multiple CNS disorders. Several pathogenic PD genes are also part of the HD PerturbDB: SNCA (α-synuclein; Furlong et al., 2000; Corrochano et al., 2011), PARK2 (parkin; Rubio et al., 2009), and PARK7 (DJ-1; Sajjad et al., 2014), and more recently, but not yet curated into PerturbDB, PINK1 (Khalil et al., 2015). Thus, perturbation of these genes in their WT form can alter mHTT-driven outcomes, indicative of a close biological relationship between their functions and mHTT pathophysiology. To extend to other neurodegenerative diseases, we first curated well-known pathogenic genes for PD, AD, eight PolyQ diseases but excluding HD, ALS, and SMA into the Neurodegenerative Disease (NeuroD) PPI gene sets, as described in Methods Section Gene Sets and shown in Table 6. These range from the smallest set of 51 genes in the ATXN2 set (for SCA2) to the largest of 2,133 genes for APP (AD), with most others in the low hundreds of genes. We also combined each PPI into its cognate NeuroD to form larger disease specific gene sets for enrichment analyses. All the gene lists are in Supplementary Datasheet 2.

**Table 6:** **Overlap of NeuroD PPI sets with HTT interactome_1619**.

**Gene ID**	**Symbol|Disease**	***n* PPI**	**n common HTT PPI**	**FDR**	**% Set NeuroD**
**PD Set**		***763***	***370***	5.4E-224	***48***
6622	SNCA	398	235	9.7E-165	59
120892	LRRK2	198	108	3.2E-69	55
11315	PARK7	147	73	6.0E-43	50
5071	PARK2	176	77	2.7E-40	44
65018	PINK1	52	25	1.2E-14	48
**AD Set**		***2354***	***400***	1.1E-65	***17***
4137	MAPT	118	67	2.2E-44	57
5663|5664	PSEN1|2	253	97	1.2E-44	38
351	APP	2133	310	3.3E-35	15
**PolyQ Set**		***1139***	***303***	2.1E-96	***27***
773	CACNA1A|SCA6	215	87	3.1E-42	40
4287	ATXN3|SCA3	98	44	1.0E-23	45
6311	ATXN2|SCA2	51	21	7.6E-11	41
6310	ATXN1|SCA1	272	74	2.7E-23	27
367	AR|SBMA	325	86	4.3E-26	26
6908	TBP|SCA17	246	57	1.0E-14	23
6314	ATXN7|SCA7	87	23	*1.5E-07*	26
1822	ATN1|DRPLA	115	28	*3.7E-08*	24
**ALS Set**		***328***	***123***	1.7E-55	***38***
6647	SOD1	84	36	1.0E-18	43
23435	TARDBP	120	46	2.5E-21	38
2521	FUS	159	54	5.0E-22	34
**SMA** 6606|6607	**SMN1|2**	***241***	***67***	1.10E-21	***28***

#### The NeuroD protein networks highly overlap the HD datasets and each other

**Figure 5:**
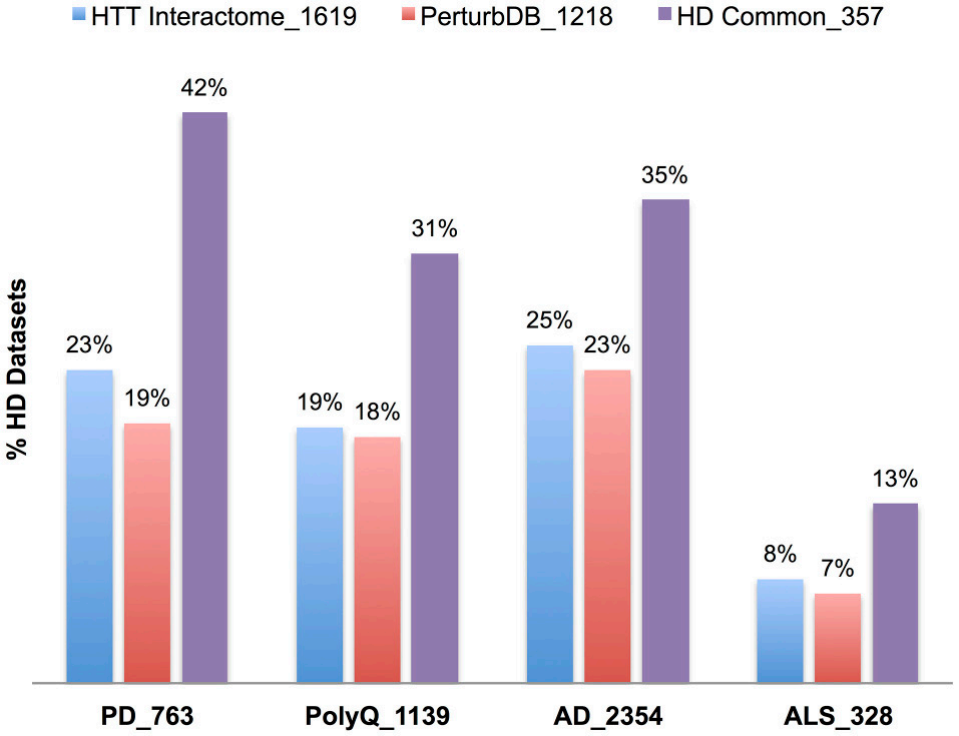
**Relative percent overlap of the HD datasets with the NeuroD PPI sets**. Percentage of the HTT Interactome, PerturbDB and HD Common (found in both sets) that overlap with PD, PolyQ, AD, and ALS PPI sets is shown. The number following the underscore indicates the size of each dataset.

We first examined the overlap of each NeuroD PPI with the HTT Interactome, as they are all protein networks centered on pathogenic disease genes. Interestingly, there is significant overlap, particularly for the PD PPIs, with 59% of SNCA (α-synuclein) at the highest (−log FDR ~165) and 44% of PARK2 at the lowest (−log FDR ~40). While these genes only account for portion of PD cases, their role in protein folding, misfolded protein processing and mitochondrial functions suggests these mechanisms are important for both diseases. The microtubule-associated tau (MAPT) PPI also highly overlaps the HTT Interactome, at 57% with −log FDR ~44. The role of tau, in its multiple isoforms and phosphorylation states for cytoskeletal functions, has just recently been implicated in HD (Fernández-Nogales et al., 2014; Blum et al., 2015; Gratuze et al., 2015; Vuono et al., 2015). Some of the PolyQ disease genes, especially the VGCC subunit in SCA6 at 40% (−log FDR ~40), points to the importance of calcium dysregulation in HD (Giacomello et al., 2013). We then combined the PPI sets into their respective five disease sets (Table 7, Figure 5), finding again the highest overlap of the HTT Interactome with the PD set (48%, −log FDR ~224). Moreover, the NeuroD PPIs overlap PerturbDB with the HD Common set again showing higher % overlap than each HD dataset (Table 7, Figure 5; SMA at 4% each for the HD single sets and 6% for HD Common). As is the case for other datasets, within PerturbDB the Drosophila *in vivo* and mammalian aggregation platforms again most highly overlap the NeuroD PPI sets (Tables S10A,B in Supplementary Datasheet 4). Thus, the overlapping NeuroD protein networks contain many modifiers of mHTT-driven outcome measures, and perhaps many are also modifiers for other neurodegenerative diseases. Finally, the NeuroD PPI sets also significantly overlap each other, with about 72% of the PD and ALS sets intersecting at least one other NeuroD set (Table S11 in Supplementary Datasheet 4; Figure S6). Indeed, the subsets of each NeuroD PPI found in common with the HTT Interactome are even more highly overlap with each other (Figure S7), suggesting an even tighter interlocking protein network for neurodegenerative pathogenic genes centered on HTT that when perturbed at different points can contribute to pathogenesis in distinct diseases. More detailed analysis of the NeuroD PPIs is presented in Supplementary Material Section 6.1 in Supplementary Datasheet 4.

**Table 7:** **Enrichment analysis of HD and NeuroD PPI datasets**.

	**HTT Interactome_1619**		**PerturbDB_1218**		**HD Common_357**	
	***n* common**	**FDR**	***n* common**	**FDR**	***n* common**	**FDR**
PD_PPI_763	370	5.4E-224	232	2.5E-111	150	5.8E-124
PolyQ_PPI_1139	303	2.1E-96	220	3.2E-64	113	3.0E-57
AD_PPI_2354	400	1.1E-65	280	4.2E-38	127	6.2E-36
ALS_PPI_328	123	1.7E-55	80	1.2E-29	47	1.5E-29
SMA_PPI_241	67	1.1E-21	48	2.4E-14	23	7.7E-11

#### The NeuroD protein networks highly intersect the exosome and HmSP datasets

**Figure 6:**
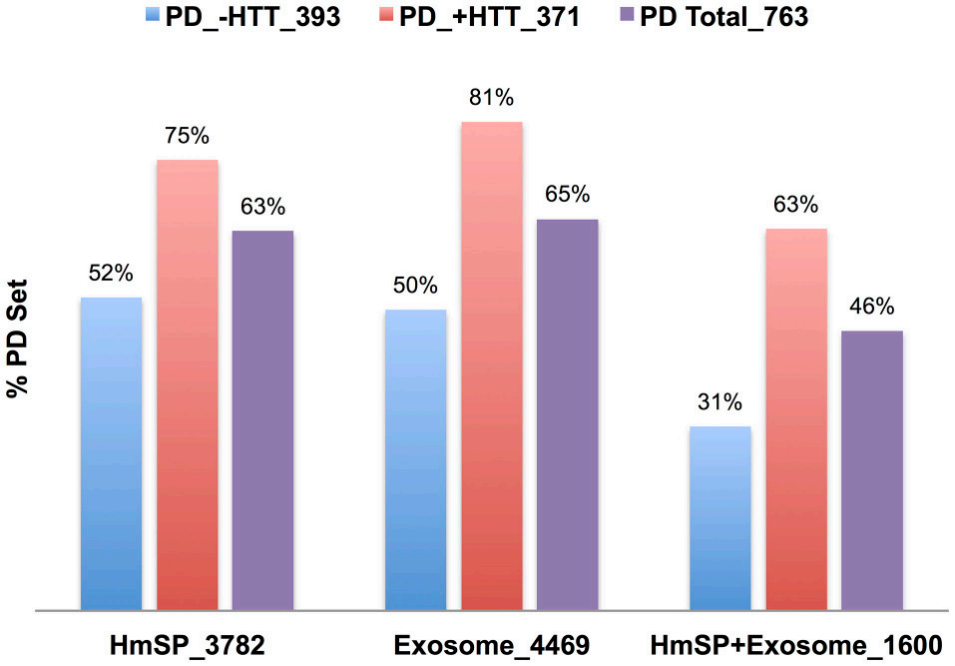
**Relative percent overlap of the PD PPI with the HmSP and Exosome DBs**. Percent of the PD PPI sets that intersect the HmSP DB, Exosome DB, and the genes found in both HmSP and Exosome DBs (HmSP-Exosome) are shown. The PD PPI sets are the entire set (PD Total), the subset found in common with the HTT Interactome (PD +HTT), or the subset with no overlap with HTT Interactome (PD no HTT). The number following the underscore indicates the size of each dataset.

Not surprisingly, the NeuroD PPIs also highly overlap the Exosome and HmSP DBs (Table 8), with % overlap ranging from mid 30s for AD to 60s for PD and ALS, and −log FDR values generally in the hundreds. The intersection with the HmSP and Exosome DBs is higher for NeuroD PPIs that are in common with the HTT Interactome (+HTT) than those that do not (−HTT), as exemplified for PD (75% and 52% respectively, Figure 6). The same pattern holds true for the Exosome DB and the HmSP-Exosome Common set (Figure 6), most with −log FDR values over 100 (Table S12 in Supplementary Datasheet 4). The same pattern of increased relative % overlap is true and perhaps even more striking for the remaining NeuroD sets, as shown in Figure S8 (the SMA set increases from 48 to 73%; data not shown). These results suggest that there are more biological links between HmSP and exosomes and the NeuroD protein networks that overlap with the HTT Interactome compared to the parts of the network that do not.

**Table 8:** **Overlap of NeuroD PPIs with HmSP and exosome DBs**.

	**HmSP DB_3782**			**Exosomes DB_4469**			**HmSP Exosome Common_1600**		
	***n* common**	**FDR**	**% Set**	***n* common**	**FDR**	**% Set**	***n* common**	**FDR**	**% Set**
PD PPI_763	480	4.9E-187	63	494	2.7E-167	65	354	1.2E-206	46
PolyQ PPI_1139	736	1.4E-306	65	440	6.3E-51	39	349	2.5E-134	31
AD PPI_2354	861	4.2E-135	37	824	6.4E-75	35	473	1.8E-108	20
ALS PPI_328	229	1.1E-100	70	203	1.3E-61	62	162	8.5E-96	49

Finally, the overlap of the NeuroD sets is higher for genes in the HmSP-Exosome Common set vs. those in each DB alone, with the exception of the PolyQ (Figure S9). This again supports a linkage between HmSP and exosome biology, and furthermore that where the two mechanisms intersect is particularly important for the pathophysiology of these neurodegenerative diseases. Again, when the NeuroD PPIs are segregated into the subsets that overlap the HTT Interactome, the relative % overlap is even higher (Figures S9A,B in Supplementary Datasheet 4). This is particularly prominent for the PolyQ PPIs, where the increased relative % overlap is obvious only for the +HTT subset. The same trend is found for the constituent subsets of HmSP (e.g., presynaptic, up-scaling and down-scaling etc.) as presented in Tables S13A–F in Supplementary Datasheet 4. Thus, as in the case for HD, there appears to be strong linkages between exosome biology and HmSP, and particularly where the two mechanisms intersect, with the other neurodegenerative diseases. As discussed for HD, experimental verification of the hypothesis is needed, using experimental platforms driven by the appropriate mutant NeuroD pathogenic genes, to determine if exosome and HmSP functions are indeed altered.

### Transcripts localized to synapses highly overlap the HD and NeuroD datasets

mHTT has long been known to affect transcription of a variety of genes, and this is postulated as a primary pathology drivers (Moumné et al., 2013). We found that a set of differentially expressed genes in postmortem HD patient caudate and motor cortex (Kuhn et al., 2007) highly overlaps the HTT Interactome and PerturbDB (unpublished results), and we plan to integrate the results presented here with new analyses of a comprehensive set of HD transcriptomic studies (Langfelder et al., 2016). Synaptic plasticity, including HmSP, ultimately involves complex transcriptional alterations over the longer term, and exosomes can carry miRNA to alter transcription in the receiving cells. HTT itself has also been postulated to be involved in trafficking of RNA granules, interacts with Ago2, and contributes to localized transcription changes at the synapses (Culver et al., 2012). Thus, synaptic control of local translation in the synapses to support synaptic function and their potential to contribute to pathogenesis deserves further investigation (Thomas et al., 2014). In the meantime, we have incorporated a large set of transcripts identified by deep sequencing of microdissected synaptic neuropil regions from hippocampal CA1 that are putatively localized to synapses (Cajigas et al., 2012) and may therefore mediate local transcription in response to rapid synaptic alterations. This set of 2,305 transcripts contains a large fraction of synaptic proteins that include signaling molecules and scaffolds. This local synaptic transcriptome significantly overlaps the HTT Interactome, PerturbDB and NeuroD PPI sets (Table 9). A detailed table of the overlap for the HD subsets is shown in Table S13 and discussed in Supplementary Material Section 7 in Supplementary Datasheet 4. An increase in the relative % overlap with each NeuroD subset that is found in common with the HTT Interactome is again observed (Table 9). Moreover, this Synaptic Localized Transcript set also overlaps the HmSP and Exosome DBs at 32 and 33%, respectively, suggesting the involvement of locally regulated transport and transcription of mRNA. Indeed, the Synaptic Localized Transcript genes that overlap with either HmSP or Exosome DBs show higher relative % overlap with the HD datasets and with the NeuroD PPI sets compared to the whole Synaptic Localized Transcript set (Figure S10). Finally, the subset found in common in the Synaptic Localized Transcripts, HmSP, and Exosome DBs shows the highest overlap for HD and all NeuroD datasets. These results suggest that these local transcripts may play a role in all both mechanisms, and likewise may be involved in HD and other neurodegenerative disease pathophysiology.

**Table 9:** **Overlap of synaptic localized transcript with HD and NeuroD datasets**.

	**Synaptic Localized Transcripts_2305**		
**HD Sets**	***n* common**	**FDR**	**% Set NeuroD**
HTT Interactomes_1619	436	1.31E-87	27
PerturbDB Total_1218	282	1.98E-40	23
HD Common_357	113	1.55E-27	31
**NeuroD SETS**			
PD All PPI_763	177	2.13E-25	23
PD +HTT PPI_371	111	3.49E-25	30
PolyQ All PPI_1139	223	1.99E-21	20
PolyQ +HTT PPI_303	86	7.79E-18	28
AD All PPI_2354	404	7.92E-27	17
AD +HTT PPI_400	119	1.39E-26	30
ALS All PPI_328	88	2.13E-16	27
ALS +HTT PPI_123	44	6.85E-13	36

### What are the therapeutic opportunities?

Querying for terms such as “kinase” under GO in Supplementary Datasheet 2 shows that Exosome and HmSP DBs are full of known drug targets. While evaluation of these drug targets as therapeutic opportunities for neurodegenerative diseases requires first the validation of exosome and HmSP functions in HD and development of therapeutic rationale, some of the drug targets are nevertheless interesting to consider because of their established links to HD, albeit under different mechanistic contexts such as cell toxicity. Prominent examples are calcium dysregulation with targets such as VGCCs that are important for presynaptic HmSP, PLK2 that opposes the action of NFκB signaling pathway at the dendritic spines to regulate homeostatic end point for excitatory synaptic adaptation (Mihalas et al., 2013), and the interaction between PLK2 and CDK5, mTOR, and EphA4 to regulate HmSP. Sharp pharmacological tool compounds are available for many of these targets and can be used to elucidate their roles in the disease context. Indeed, if particular functional parts of HmSP and exosomes are altered in different disease contexts to give rise to the corresponding clinical features, then an exciting possibility is that such imbalance in HmSP could be restored by pharmacological perturbation of the critical signaling molecules. Given that HmSP is by definition context-dependent, it may well be that drugs that impact HmSP function could be useful in multiple indications. The first order of business, however, is to simply determine if any of the multiple form of HmSP and exosome functions are altered by the NeuroD pathogenic genes, as has already been shown for SMN for SMA and the calcium channel subunit CACNA1A for SCA6. If the answer is yes, and depending on what the alterations are for each pathogenic genes, a promising avenue of new drug discovery programs will open up for the neurodegenerative diseases. A preliminary, generalized flow scheme for such a drug discovery program is shown in Figure S11. A key recommendation is the cross-fertilization between mechanistic experimental platforms, leveraging the advantages of the *in vivo* context of the *Drosophila* platform and the utility of mechanistically specified cell culture platform into new drug discovery assays. Moreover, exosomes may provide disease biomarkers from patients. Most interesting is that given the extensive overlap between the two mechanisms and the protein networks of multiple neurodegenerative diseases, findings for each of the diseases may be relevant for the others.

## Conclusions

We have developed a database-enabled systems biological methodology to systematically organize, curate, and analyze large sets of HD data and link them with their gene IDs to the published literature based on the co-cited gene IDs and MeSH terms in each PubMed article. This allows for efficient manual scanning of the literature for mechanisms of interest based on co-citation of genes of interest from datasets and MeSH terms of interest. The identification of mechanisms in the datasets then leads to building of mechanistic gene sets for enrichment analyses and rank ordering. We deployed this methodology to uncover novel biological mechanisms of exosomes and homeostatic synaptic plasticity that are both implicated in the normal function of HTT and the pathology of mHTT. These two mechanisms are also linked to protein-protein networks centered on pathogenic genes for PD, AD, ALS, SMA, and other polyQ diseases. Moreover, all these protein networks, including that of HTT, also highly intersect each other, indicating that they may all engage exosome biology and HmSP at distinct points that may ultimately contribute to the different disease pathologies. These hypotheses can be verified by straightforward experiments in the appropriate platforms. If confirmed, there will be multiple potential drug targets that can be evaluated as therapeutic opportunities, and novel screening assays and biomarkers will also be developed and tested as tools for therapeutic development for multiple neurodegenerative diseases.

## Author contributions

JW, MP curated and built the databases; JW performed the biological analyses; PL and SH performed the statistical analyses; JW wrote the manuscript; all authors edited the final version of the manuscript.

## Funding

This work was supported by CHDI Foundation Inc.

### Conflict of interest statement

The authors declare that the research was conducted in the absence of any commercial or financial relationships that could be construed as a potential conflict of interest.
