# Supplementary material for: Exosomes and Homeostatic Synaptic Plasticity Are Linked to Each other and to Huntington's, Parkinson's, and Other Neurodegenerative Diseases by Database-Enabled Analyses of Comprehensively Curated Datasets
Source: Front Neurosci. 2017 Mar 31;11:149. doi: 10.3389/fnins.2017.00149 (PMC5374209; doi:10.3389/fnins.2017.00149)
Supplement: Supplementary file 4 [file DataSheet4.docx]

***Supplementary Material Datasheet S4***

Exosomes and homeostatic synaptic plasticity are linked to each other and to Huntington’s, Parkinson’s and other neurodegenerative diseases by database-enabled enrichment analyses of comprehensively curated datasets

**James K.T. Wang*, Peter Langfelder, Steve Horvath, Michael J. Palazzolo**

*** Correspondence:** Corresponding Author: [jktwang2@gmail.com](mailto:jktwang2@gmail.com)

1. **Detailed analysis of PerturbDB and the HTT Interactome**

**1.1 PerturbDB can be segregated into major experimental platforms**

The HD database allows us to easily segregate and query PerturbDB and the HTT Interactome. We segregated the PerturbDB into the *in vitro* and model organism platforms that constitute the bulk of the data, and examined the degree of overlap between these experimental platforms and to other datasets (see Supplementary Figure S1 for an example of the Access Form designed to allow easy queries of this type). The validity and relative merits of the many mHTT-driven experimental platforms have long been debated, for example the potentially inappropriate and misleading biological context of fly HD models because of their wide evolutionary distance from mammals; or the incompatibility of acute *in vitro* cell culture toxicity of overexpressed mHTT fragments with the progressive and late onset nature of HD. The debates, however, have not been informed by an agnostic examination of all available data because of the lack of a comprehensively curated dataset. We now provide this capability, and the first question we addressed is whether gene sets from different PerturbDB experimental platforms show significant overlap, which if true would suggest common underlying biological mechanisms. Arguably the most valuable dataset in PerturbDB for drug discovery is the genetic and/or small molecule perturbations that impact HD *in vivo* rodent models, as this is generally considered to be the highest hurdle for drug target nomination. This set numbered a rather substantial 100 genes, but it should be noted that we included all publications showing efficacy in even a single outcome measure, as we made no judgment on data relevance or quality for inclusion, aside from the criteria used for the PerturbDB curation. However, other investigators can filter these data as they see fit by using the curation tags in PerturbDB. Perhaps not surprisingly, these 100 genes have high degree of overlap with the other major HD experimental platforms discussed below, with 64 found in common (see Supplementary Datasheet S1), as the rodent *in vivo* studies are most often predicated on rationale from *in vitro* or non-rodent experimental platforms. As these genes are part of the current analysis, and as they are already subject to more intensive scrutiny in the field, we did not segregate them for detailed study on their own. Indeed, the complexity of the *in vivo* rodent biological context leads to endpoints that generally can be mediated by multiple distinct mechanisms. Rather, these *in vivo* data can best be evaluated when mechanistic hypotheses are proposed as a late-stage validation step. Thus, we focus on the bulk of the perturbation data from the simpler experimental platforms.

The vast majority of the experimental platforms in PerturbDB, from yeast to flies to rodents, expressed short fragments of mHTT (exon1 or up to ~580 amino acids in the N terminal region), though in some cases full-length mHTT or in the other extreme only pure polyQ was used (primarily in a whole genome screen for aggregation of pure polyQ in intact *C. elegans*). As might be expected, the bulk of the HD literature focused either on specific hypothesis or on selected datasets, and there are only a few cases of agnostic large scale experiments, such as unbiased whole genome screens in *C. elegans* and *Drosophila* cell cultures, and targeted screens of the “druggable genome” in mammalian cell culture platforms. In the “druggable genome”, one would expect to find many signaling genes that are widely connected hub genes in the database. This is confirmed by querying the Master Data Table (Supplementary Datasheet S2) under Gene Summary or GO for likely druggable activity terms such as “kinase” (~200 genes) or “receptor” (~300 genes). Many of these genes also serve pleiotropic biological functions and could function in very different experimental platforms to mediate distinct outcome measures. It is likely that there are significant gaps in this HD perturbation gene set in biological areas that have not yet been widely studied. Nevertheless, a very large number of 1,218 unique genes still qualified as putatively causally linked to HD (excluding those that were tested but did not impact mHtt-dependent outcomes, which are also curated in PerturbDB), presenting a substantial obstacle to a comprehensive analysis for target nomination and prosecution.

When segregated by the experimental platforms, one of the smallest sets in the PerturbDB consists of 21 genes that are tagged to human data as they are from association studies of modifiers of age of onset in HD patients, or from studies of candidate drugs for symptomatic treatments. The other small sets are the least often utilized models of zebra fish (11 genes) and yeast (58 genes). These three sets of genes are not subject to detailed study on their own in the current analysis. After excluding genes that are tagged *exclusively* to human, zebra fish, yeast and rodent *in vivo* HD models from the 1,218 genes in the PerturbDB, 1,123 genes remain in the *in vitro* and model organism platforms. These genes can be segregated into six experimental platforms and outcomes. The *Drosophila in vivo* phenotypes set contains 243 genes from multiple studies (see Supplementary Datasheet S1 for references) that are modifiers of phenotypes resulting from retinal or pan-neuronal expression of mHTT that include eye degeneration, anti-geotaxic climbing behavior, and larval survival or adult lifespan. The *C. elegans in vivo* set contains 344 modifiers of behavioral phenotypes due to expression of mHTT in subsets of worm neurons, with the largest set from an RNAi screen using the touch sensitivity assay (Lejeune et al., 2012). The *Drosophila* cell culture aggregation set contains 138 genes that resulted from whole or partial genome screen of modifiers of mHTT aggregation in fly cell culture systems. The *C. elegans in vivo* polyQ aggregation set came from an early whole genome screen that produced 173 modifiers, primarily enhancers, of macro-aggregation of pure polyQ of 46 or 54 glutamines (Nollen et al., 2004), and is unique in the database due to its lack of any HTT context. However, some of the genes have recently been validated in a mammalian mHTT aggregation platform and are therefore relevant for HD (Teuling et al., 2011), and this gene set is included for analysis. The mammalian aggregation set contains 190 genes that impacted aggregation of mHTT in a variety of mammalian contexts, primarily in the cell culture platform. Finally, the mammalian cell culture toxicity set contains 336 genes that impacted the mHTT-driven cell toxicity in various neuronal and non-neuronal cell culture platforms. In Table S2A the number of genes unique to each platform and the number that overlap with each of the other platforms are shown as a matrix table, together with the FDR values from testing each of these gene sets as part of an enrichment analysis against the reference database of gene sets (see Methods). Overlap of each of the platform with the HTT Interactome is also shown in this Table.

**1.2 The HD experimental platforms in PerturbDB highly intersect each other**

Because a number of pathways and constituent genes that are of high interest for HD and the “druggable genome” have been studied in multiple experiments, one might expect a degree of overlap between the platforms, particularly if the genes found in common are functionally pleotropic and capable of playing a role in mHTT pathophysiology in a variety of cellular and biological contexts. This would be counter-balanced by the fact that these data were collected independently from a myriad of different platforms and experimental conditions. Indeed, there is a substantial range of overlap between each platform with one or more of the others, the lowest at 19% for the *C. elegans in vivo* phenotypes platform (64/344 genes found in one or more other platform and the remaining 280 genes are unique to this platform), followed by mHTT aggregation in *Drosophila* cell culture at 34% (47/138), 38% (65/173) for the *C. elegans in vivo* pure polyQ aggregation, 39% (130/336) for the mammalian cell culture toxicity, 40% (97/243) for *Drosophila in vivo* phenotypes, and a high of 65% (124/190) for the mammalian mHTT aggregation platform (Table S2A). This degree of overlap between multiple platforms suggests that these perturbation data could be informative for elucidating mHTT pathophysiology and deserve further analysis. The largest overlap is the 83 genes between the mammalian mHTT aggregation and the cell culture toxicity platforms, with –log FDR ~94, likely because these two outcome measures are often studied together in cell culture platforms. Interestingly, the next two highest overlap is between the *Drosophila in vivo* phenotypes with the mammalian cell culture toxicity and mammalian mHTT aggregation platforms (–log FDR ~42), even higher than its overlap with mHTT aggregation in *Drosophila* cell culture (24 genes, –log FDR ~19). Closer examination of PerturbDB shows that 30 genes are found in all 3 major experimental platforms (see Supplementary Datasheet S2 for the gene IDs, and Supplementary Figure S3A showing the Venn diagram of the overlap between the platforms, and the HTT Interactome). Moreover, these 3 major platforms account for all 64 *in vivo* rodent genes that overlap at least one other experimental platform, with 52 genes (or 16%) from the cell culture toxicity platform, 29 (15%) from mammalian mHTT aggregation, and 23 (9%) from *Drosophila in vivo* phenotypes, while the remaining platforms contribute only 6 (worm *in vivo* phenotypes), 5 (worm *in vivo* pure polyQ aggregation) and 2 (fly cell culture mHTT aggregation) genes (see Supplementary Datasheet S2). The intersections of the 3 major platforms for these 64 rodent *in vivo* genes are even more striking (compare Figure S3A to Figure S3B). Moreover, the high percentage in these 3 gene sets of signaling pathway genes such as kinases and ion channels, partly due to efforts to screen the “druggable genome”, also make it more enriched for genes with pleotropic functions that could impact distinct mHTT-driven endpoints in different biological contexts. We therefore postulate that the value of the *Drosophila* *in vivo* phenotype platform lies in its intact CNS context that underlies the neuropathology and behavioral abnormalities, as the pathology is likely mediated by important pathways and genes that are conserved across the evolution distance. In the cell culture context, on the other hand, mHTT may be engaging pleotropic signaling pathways that are linked to cytotoxicity in culture, but are then linked to other relevant biological functions *in vivo* that pertains to disease pathology. Finally, mHTT aggregation, perhaps the most proximal outcome measure to HD pathophysiology, shows a highly significant overlap with the mammalian cell toxicity and fly *in vivo* phenotype. Interestingly, it also significantly overlaps the worm *in vivo* pure polyQ aggregation platforms (32 genes, –log FDR ~29), but not as much with the fly cell culture mHTT aggregation (11 genes, –log FDR ~10). This suggests that despite the lack of HTT biological context in the pure polyQ aggregation assay *in vivo*, it is at least impinging on some shared mechanisms with mHTT aggregation in mammalian cell culture, and indeed some of the genes in the pure polyQ platform have been validated in a mammalian mHTT aggregation assay (Teuling et al., 2011). The aggregates and inclusions containing mHTT are likely complex mixtures of distinct forms of insoluble proteins, and the overlap with *in vivo* pure polyQ aggregation suggests that there is commonality in handling misfolding proteins *in vivo* in model organisms and in mammalian cells and tissues. The worm *in vivo* behavior platform shows relatively low intersection with other datasets, but being from a whole-genome screen, this could reflect a lack of testing of these genes in the other platforms with studies that had been hypothesis-driven. The number of intersecting genes is nevertheless in the hundreds and may still be useful in evaluation of the enrichment analysis results.

**1.3 The HTT Interactome subsets highly intersect each other and PerturbDB**

The HTT Interactome is curated from several studies using multiple technologies on different tissue sources including brain regions of HD mouse models, cells and yeast-two-hybrid assays (see Methods for details). The total HTT Interactome (wild type and mutant) contains 1,619 proteins, and as such represent a diverse set of proteins that possess protein-protein interactions with HTT or are part of a large protein complex that includes HTT. Such a large interactome is consistent with the role of HTT as a scaffolding protein playing multiple roles in many different cells and subcellular compartments. The list of genes and detailed sub-categories and curation for each HTT interactome dataset are shown in the Supplementary Datasheet S2. By combining and curating all the HTT interactome studies, we expect to find significant overlap between the constituent subsets, which would strengthen the case for using the entire dataset for our analysis. Indeed, we do find significant intersections between the subsets from the published studies, with approximately 40-50% of each interactome overlapping one or more of the other sets (Table S2B). The most significant overlap is between the two HTT pull-down interactomes from HD mouse model brains, the cytoplasmic/membrane bound brain HTT interactome from the CAG140 knock-in HD mouse (518 proteins total) and the brain regions at different ages of the BACHD mouse (741 proteins total). The 167 proteins found in common between these two datasets has an –log FDR ~119. Thus, we conclude that the HTT Interactome is a robust dataset amenable for further analysis. We then examined its intersection with PerturbDB, as a significant overlap would indicate a causal relationship between the biological function of HTT in an interactome and the pathology driven by mHTT. We find that there is a highly significant overlap of 357 genes (~30% of each dataset) between the PerturbDB and HTT Interactome, with an –log FDR ~131 (Table S2A). As expected there is also significant overlap between PerturbDB subsets and the HTT Interactome (see Table S2A), the top 3 being the platforms from Figure S3 with the *Drosophila* *in vivo* phenotype at the highest (123 genes, 51% of the fly set, –log FDR ~66). This confirms and extends the previous finding that a high percentage of the BACHD brain HTT Interactome subset is validated in the fly *in vivo* phenotype platform (Shirasaki et al., 2012). Indeed, the fly platform has been deployed as a secondary assay for hits from mammalian cell culture (Miller et al., 2010; Miller et al., 2012), as this platform is often used to leverage its *in vivo* CNS nature, experimentally tractable short lifespan, and the abundance of fly genetic tool kits.

The *Drosophila* *in vivo* platform is followed closely by the mammalian mHTT aggregation (90 genes, 47% of the set, –log FDR ~47), and then the cell culture toxicity platforms (110 genes, 33% of the set, –log FDR ~38), and indeed 19 of the 30 genes common to all 3 platforms are also part of the HTT Interactome (Figure S3A). Strikingly, the Venn diagram of the overlapping PerturbDB platforms overlaid with the HTT Interactome shows that only a minority of genes in each platform has no intersection with any other sets. Indeed, the mammalian mHTT aggregation platform has only 4 genes (out of 190 total, or 2%) that are not found in the other two platforms or in the HTT Interactome, while the fly *in vivo* platform has somewhat more, 29 genes (of 243 total, or 12%) and the mammalian cell culture toxicity platform has the most, 117 genes (of 336 total, or 35%). These results are consistent with the hypothesis that mHTT aggregation (in all its forms with distinct pathological potential, which is not distinguished in the database) is most proximal to mHTT pathophysiology, while the *Drosophila in vivo* platform provides a relevant intact CNS context to manifest the pathology, and the cell culture toxicity may still offer value in the smaller subsets that overlap the other HD platforms. Thus, the large HTT interactome contains biologically and pathologically relevant protein networks that contribute not only to the normal function of HTT but also to aspects of mHTT pathophysiology. We therefore conclude that both of these HD datasets are sufficiently large and significantly intersected to be useful as the basis for further mechanistic analyses.

There is also significant overlap of the HTT Interactome with PerturbDB, as might be expected because some of the interactome studies successfully carried out validation of subsets of the HTT interactors as perturbation genes in HD platforms (Kaltenbach et al., 2007; Shirasaki et al., 2012). However, the integration of these two datasets into a database allows one to ask whether this overlap is true across all published datasets. Taken as a whole, the degree of overlap of HTT interactors and HD perturbation genes is striking. Table S1A shows the overlap and FDR values between each of the PerturbDB platforms and the entire HTT Interactome, while Table S1B shows the converse of intersection of each of the HTT interactomes with the entire PerturbDB. The most significant overlap with the HTT Interactome is the *Drosophila in vivo* platform, 51% of which is part of the HTT Interactome (–log FDR ~66), followed by the mammalian mHTT aggregation platform (47%, –log FDR ~47) and cell culture toxicity (33%, –log FDR ~38), and the *C. elegans* pure polyQ aggregation set (46%, –log FDR ~39). As noted previously, the nematode pure polyQ aggregation set has a significant overlap with the mammalian mHTT aggregation set, despite its lack of HTT context. It is therefore interesting that half of the overlap between the nematode and the mammalian cell culture mHTT aggregation sets (32 in common) are also found in the HTT Interactome. Overall, the entire PerturbDB shows an overlap of 30% with the HTT Interactome with a –log FDR ~131.

1. **SynapseDB and its subsets are highly enriched in the HD datasets**
   1. **Curating the SynapseDB and its overlap with the HD datasets**

The relative paucity of gene sets for specific neuronal functions in GO, KEGG pathways and other public databases led us to curate a large and broad set of 3,549 genes involved in some aspects of neuronal and synaptic functions as SynapseDB (see Methods for details). It should be noted that many of these genes perform pleotropic functions or are hub genes in signaling networks underlying both generalized cellular and specialized neuronal functions. Thus, as is true for the ubiquitously expressed HTT, their links to the neurodegenerative aspects of HD would be manifest most strongly in specific neuronal contexts. To summarize, SynapseDB highly intersects the HTT Interactome (52% of the latter, –log FDR ~280) and PerturbDB (40%, –log FDR ~101), and even more so the genes found in both HTT Interactome and PerturbDB (65%, –log FDR ~100) (Supplementary Table S3). This supports the prevalent view that HTT plays important roles in neuronal and synaptic biology, and that mHTT pathology is linked to disruption of, and/or gain of defects in, at least certain aspects of HTT neurobiology. We postulate that defining more focused mechanistic subsets within this large SynapseDB may point to specific neuronal defects in HD.

The HTT Interactome is known to contain a large number of synaptic proteins, and indeed 52% of the entire interactome are found in common with the Synapse DB, with an –log FDR ~280. The two constituent subsets from the HD mouse brains (BACHD and CAG140) show the highest intersection with the SynapseDB, at 68% and –log FDR ~238, and 62% and –log FDR ~130, respectively (Table S3). While enrichment for synaptic genes was noted in both original publications, perhaps not surprising as the HTT interactors are derived from brain extracts, such a remarkable degree of overlap with the larger SynapseDB confirms the neuronal and synaptic relevance of the HTT Interactome. The other HTT Interactome subsets also show significant intersection, with the Striatal Cell Line at the lowest (34% of the set) but still with a significant –log FDR ~15. Importantly, overlap of PerturbDB with the SynapseDB is also high, at 40% with an –log FDR ~101 (Table S3). The set of 357 genes found in both the HTT Interactome and PerturbDB (HD Common) intersects the SynapseDB at an even higher relative percentage (65% or 233 genes, and –log FDR ~100) compared to each HD dataset. As the two HD datasets also highly overlap each other, such an increase in the relative % of overlap of the HD Common set with SynapseDB is indicative of a functional link between synaptic functions and HTT biology that is critical for mHTT pathophysiology. Thus, not only is the HTT Interactome highly enriched for synaptically relevant genes, many of these genes may also be causally linked to mHTT pathophysiology. Within PerturbDB, the *Drosophila in vivo* phenotypes platform shows the highest overlap, at 54% and a –log FDR ~41, with SynapseDB. The mammalian cell culture toxicity and mHTT aggregation platforms also significantly intersected SynapseDB, at 43% with –log FDR ~32, and 48% with –log FDR ~25, respectively. It is noteworthy that of the three protein aggregation platforms, the mammalian mHTT and the *C. elegans* *in vivo* pure polyQ aggregation platforms overlapped SynapseDB (48% or 50% of each set, with –log FDR ~25) more significantly than the *Drosophila* cell culture platform (32%, –log FDR <5). Thus, fly cells in culture with mHTT aggregation as the readout appears not to uncover genes that functionally translate directly into other species and platforms, in stark contrast to the *Drosophila in vivo* phenotypes platform with eye degeneration and behavioral deficits as readouts.

- 1. **Analyzing subsets of the SynapseDB for overlap with the HD datasets**

Because SynapseDB is a very large gene set, we predict that curating its component subsets linked to specific synaptic mechanisms relevant in HD will show enhanced enrichment. For example, we segregated from the SynapseDB a “Postsynaptic Proteome” consisting of 1,097 genes (see Methods) found in the postsynaptic region and likely more specifically involved in local synaptic functions. 49% of this subset is intersected with the HTT Interactome, compared to 24% of SynapseDB (–log FDR >300 vs. ~280 respectively) (Table S3 and Figure S4). The Postsynaptic set also shows a higher relative % overlap for PerturbDB at 21% vs. 14% for the SynapseDB, and similarly for the combined HD Common set (14% vs. 7% for the SynapseDB). Within the HTT Interactome, all the subsets show the same pattern of increase in the relative % overlap with the Postsynaptic Set. However, within PerturbDB, only the *Drosophila in vivo* platform and the worm *in vivo* polyQ aggregation platforms show the relative increase in % overlap (4% and 2% of SynapseDB vs. 7% and 5% of the Postsynaptic subset, respectively), while the other PerturbDB platforms do not (Table S3). The Venn diagram in Figure S4 provides another view of the intersections between the HD datasets and SynapseDB and its Postsynaptic subset, and the relative enrichment of the HD Common in SynapseDB and Postsynaptic sets. Thus, narrowing down SynapseDB to subsets based on specific function or mechanism should lead to more specific mechanistic insights, as revealed by a relative increase in intersection of these subsets in with the HD datasets.

- 1. **A small set of genes for synaptic vesicle functions very highly overlap with the HD datasets**

Further analysis of the SynapseDB and HD data revealed a coherent set of genes from the model organism and the mHTT aggregation platforms that are important for the biology of synaptic vesicles, which we termed “vesicle dynamics” to include functions such as endocytosis, vesicle acidification, vesicle fusion, and ESCRT sorting. These 27 genes are found in both PerturbDB and the HTT Interactome, and in the perturbation studies there is a consistent pattern of primarily loss of function or knockdown in the *Drosophila* or the *C. elegans* *in vivo* phenotypes platforms improving the outcome measures (Table S4). Such a relationship has also been shown by Kaltenbach et al. (2007) in their study of an HTT interactome and validation of some of the genes in the *Drosophila* HD perturbation platform. These data are consistent with a synaptic over-activation in the HD model organisms that when countered by genetic perturbation lead to amelioration of the mHtt phenotypes. However, this hypothesis is contrary to the generally accepted thesis that reduced functionality or even dis-connectivity of the striatal medium spiny neurons from cortical input is a key synaptic dysfunction that leads to motoric symptoms in HD. We therefore speculated that that these genes are more likely to impact HD biology via another synaptic mechanism rather than the basic neurotransmission machinery. Indeed, 4 of these genes are required for exosome functions: syntaxin 1A and RAB11, for exosome release; dynamin and clathrin, for exosome uptake. These genes therefore play dual roles in the basic machinery of neurotransmission and for exosome release and uptake. This suggests that their role as HD modifiers involve basic neurotransmission but rather exosome-mediated synaptic plasticity that is likely specific and context dependent. Disruptions in such higher order modulatory mechanisms are more amenable than in basic neurotransmission to account for the slow progressive onset of disease phenotypes.

1. **Exosome synaptic PPI and ProteinDB are highly enriched in the HD Datasets and subsets**

The 892 genes in the Exosome Synaptic PPI show highly significant intersection with PerturbDB (17% of PerturbDB, –log FDR ~62) and HTT Interactome (18%, –log FDR ~112), and the HD Common set (33%, –log FDR ~70), as shown in Table S4. For the HTT Interactome, all the subsets except for the Striatal Cell Line show mid to high 20% range of overlap with the exosome set, and –log FDR ranging from ~23 (Cell-Tissue Pull Down_Y2H) to ~75 (BACHD brain). For PerturbDB, the *Drosophila* *in vivo* phenotypes and the mammalian mHTT aggregation platforms show the highest overlap (25% with –log FDR ~26; and 27% with –log FDR ~24, respectively), reminiscent of the preponderance of genes from these two sets in the exosome functional gene set shown in Table 1 in the main text. These are then followed by the nematode *in vivo* pure polyQ aggregation and mammalian cell culture toxicity sets (21% with –log FDR ~13; and 18% with –log FDR ~18, respectively), while the remaining *C. elegans in vivo* phenotype and *Drosophila* cell culture mHTT aggregation platforms show little significant overlap. This is similar to the intersection of the HD datasets with SynapseDB, and is consistent with a great deal of overlap of genes important for synaptic functions and for exosome release from neurons, and the potential role the latter plays in HD pathophysiology. Indeed, as expected, the Exosome Synaptic PPI set also highly overlaps SynapseDB, with 496 common genes or 56% of the exosome set. Of these 496 genes, 45% and 28% are also found in the HTT Interactome and PerturbDB, respectively (results not shown; query can be carried out using Supplementary Datasheet S2).

For the set of proteins detected in exosomes (Exosome ProteinDB), all the subsets of the HTT Interactome intersect it at remarkably high 41% to 64% each of the latter found in common with the former. The BACHD and CAG140 brain sets are at the highest overlap of over 60% of each with Exosome ProteinDB, at –log FDR at ~175 and ~108, respectively (Table S4). Thus, regardless of the source of the HTT Interactome subsets, there is highly significant overlap with the Exosome ProteinDB, suggesting that a large fraction of proteins in complex with HTT (and/or mHTT) are either cargo or components of exosomes from a multitude of cell types, further supporting the hypothesis that there may be a functional link between HTT function and exosome biology. Each of the PerturbDB platform is also at 35% or higher overlap with the Exosome ProteinDB, with the most significant overlap in the *Drosophila in vivo* phenotype platform (52%, –log FDR ~52). Rather striking is that 72% of the *C. elegans in vivo* modifiers of pure polyQ aggregation are found in common with the exosome proteins (–log FDR ~17), suggesting that modifiers of general protein metabolism and processing of misfolded proteins may be linked to exosome biology. For the HD Common genes found in both PerturbDB and the HTT Interactome, 63% overlap the exosome set, with a –log FDR ~79. When we segregated out the set of 739 CSF exosome proteins from the Exosome ProteinDB, there is still highly significant overlap with the HTT Interactome (12% of the latter, –log FDR ~52), PerturbDB (8%, –log FDR ~18) and the HD Common (19%, –log FDR ~32). Interestingly, the *Drosophila in vivo* phenotypes platform now shows the highest and most significant overlap with the CSF exosome protein set (16% of the *Drosophila* set, –log FDR ~14), while the other platforms fall below our arbitrary threshold of –log FDR 10 for in-depth analysis. These data suggest that a significant portion of HTT Interactome proteins may well be carried by and/or are components of exosomes, and that perturbation of some of these could impact HD phenotypes. As with the Exosome Synaptic PPI set, there is a large overlap of the Exosome ProteinDB with SynapseDB (1,260 genes found in common or 31% of the former). Not surprisingly then, a large majority of the genes found in common in both Exosome ProteinDB and the HD datasets are also in the SynapseDB. Of the HD dataset genes that intersect the Exosome ProteinDB, 64% of the HTT Interactome, 52% of the PerturbDB, and 74% of HD Common set are also found in SynapseDB. When the Exosome synaptic PPI and the Exosome ProteinDB are combined, a total of 4,469 genes then form the Exosome DB which we also used for enrichment analysis (see Table 5 in the main text) on its own or together with the HmSP datasets.

1. **Exosomes, β-catenin, and glial-neuronal communication may play a role in HD**

While exosome function in the CNS is not well understood, and its link to HD not yet experimentally tested, the finding that neuronally released exosomes regulate the expression of GLT1 in astrocytes (Morel et al., 2013) is particularly intriguing in the context of HD. GLT1 expression is downregulated in HD and this is thought to contribute to excess glutamate in the synapses and HD pathology (Huang et al., 2010; Miller et al., 2008). The Drosophila version of the glial glutamate transport is also downregulated in the presence of mHTT (Liévens et al., 2005). Indeed, up-regulation of GLT-1 by a variety of means appears to ameliorate HD phenotypes (Estrada-Sánchez and Rebec, 2012; Miller et al., 2008; 2012; Sari et al., 2010). If the GLT1 downregulation in HD involves exosomes released by neurons, it will add to the emerging role of glial dysfunction in the context of HD (reviewed in Hsiao and Chern, 2010) and provide yet another avenue in which exosome-mediated neuronal-glial communication is dysfunctional in HD. Notably, the vast majority of fly HD modifiers were identified with pan-neuronal expression of mHTT, and are therefore specific for this artificial in vivo context lacking mHTT expression in non-neuronal cells. Moreover, specific glial expression of mHTT in Drosophila also leads to pathological behavioral phenotypes, and the small set of modifiers identified in this glial HD model has very limited overlap with the neuronal modifiers (Besson et al., 2010; Dupont et al., 2012; Khalil et al., 2015; Liévens et al., 2008), being limited to the chaperone protein HSP70 and more interestingly the pleotropic signaling molecule β-catenin. That β-catenin modifies the mHTT-dependent phenotypes whether mHTT expression is pan-neuronal or in glia only is particularly interesting, because it is also a key player in the wnt signaling pathway that transduces the exosome function at the NMJ, in which glia also plays an important role (Kerr et al., 2014). Thus, as discussed above, the correlation of exosome dysfunction at the NMJ with behavioral phenotypes can now be extended to both neuronal and glial expression of mHTT in CNS. Whether β-catenin and other genes are modifiers of the cell type-selective exosome dysfunction can now be tested and correlated with their ability to modify mHTT-driven behavioral phenotypes. The Drosophila in vivo platform therefore provides the opportunity to test whether the effects of mHTT on exosome biology manifest in distinct cell types at different developmental stages: motor neuron terminals and glia in the NMJ of larval flies vs. CNS neurons and glia of adult flies. More importantly, translation of such results into the mammalian context, for example to test neuronally released exosomes carrying miRNA that regulates expression of the glutamate transporter GLT1 in astrocytes is affected by mHTT (Morel et al., 2013), could open up new area of investigations of HD pathophysiology.

1. **Overlap of HmSP DB with the HD and exosome datasets**
   1. **Curating PPIs of genes for specific forms of HmSP**

We curated the 143 HmSP functional genes to identify the 65 required for presynaptic, postsynaptic, or glial forms of HmSP (see Table S6). In *Drosophila* larval NMJ, presynaptic HmSP is induced by genetic or pharmacological blockade of postsynaptic receptors, which results in retrograde signals that cause precise counter-balancing compensatory increase in presynaptic transmitter release. Genetic perturbation studies have revealed a number of key genes and signaling mechanisms required for this homeostatic compensation (reviewed in Frank 2014). These signaling and transcriptional pathways converge on the regulation of calcium influx at the active zone via VGCCs of the P/Q type (Cav2.1) to alter availability of the readily releasable pool (RRP) of transmitters. The HmSP signaling pathways include Eph receptors-ephexin and Cdc42; the BLOC-1 and SNARE complexes at the active zone; and Rab3-GAP and RIM regulating the RRP (Frank, 2014; Lazarevic et al., 2013). We constructed PPI sets of each of these genes, and binned them into two categories: Eph signaling to VGCCs (707 genes), regulation of RRP of transmitters (516 genes), plus all of them combined into a single set as the Presynaptic HmSP genes with 875 genes total (348 genes are found in common in the two sets, indicative of close biological relationships between these aspects of presynaptic HmSP). We also constructed a set of PPI based on the genes required for retrograde signaling, which involves mTOR, S6 kinases, EIF4E/B and EIF4EBP1/2 (752 genes total). At the transcriptional level, transcription factors including SMAD and PAX members and SMN at least partly modulate the inversely correlated expression of the *Drosophila* *shaker* and *shal* potassium channel genes (orthologous to human Kv1 and Kv4 families, respectively), forming a set of 684 genes. We then curated PPIs for genes required for synaptic upscaling (881 genes) or downscaling (1,282 genes), and HmSP mediated by glial release of TNFα that mediates synaptic scaling on GABAergic synapses (686 genes). Finally, we also curated a set of PPI based on upscaling mediated by retinoic acid via its receptor RARA, and the Fragile X gene FMR1 (365 genes) (Chen et al., 2014) and a set of PPI for CAMKII and Debrin (319 genes), which are important for the role of calcium in HmSP in general. When combined together, these 8 PPI sets result in an overall HmSP set of 3,782 genes, including those remaining from the 143 HmSP Functional set.

- 1. **Overlap of the PPIs of specific forms of HmSP with the HD and exosome datasets**

We first examined the overlap between these HmSP gene sets representing protein networks involved in the specific forms of HmSP, and find that there is indeed significant overlap between the 8 PPI sets, as all of them sharing 50% or more of genes with at least one other set (Table S7). The “Unique genes % Set” shows the percentage of each protein network gene set that isn’t shared with any other set. The transcription set has the lowest overlap, with 50% of the set being unique, while most of the rest are in the 40% range with the calcium set (CAMKII-DBN) showing the highest overlap, at only 31% being unique to the set. Thus, these results show that there are multiple overlapping protein-protein interaction networks linking the critical gene products that mediate specific forms of HmSP, suggesting that there are tightly controlled but widely distributed signaling and other functional networks to coordinate these critical neuronal modulatory functions. We then examined their intersection with the HD and exosome datasets.

There is highly significant enrichment of the HD datasets with the combined HmSP set of 3,782 genes (see Table 5 in main text), and with each of the individual HD subsets (Table S8). The highest overlaps amongst the HTT Interactome are the BACHD (58%, –log FDR ~147), CAG140 brain (57%, –log FDR ~98), and the cell-tissue set (63%, –log FDR ~73). Amongst the PerturbDB the highest are the *Drosophila in vivo* platform (59%, –log FDR ~48) and mammalian mHTT aggregation (55%, –log FDR ~55). For the HD Common set of genes found in both PerturbDB and HTT Interactome, the overlap increases to 71% with an –log FDR ~117. Strikingly, the 1,600 genes found in both the HmSP DB (42%) and the Exosome DB (4,469 genes, or 36%) also highly intersect all the HD datasets (Table S8). The intersections between the HD datasets (HTT Interactome and PerturbDB) and the HmSP DB, and where they overlap with the Exosome DB, are shown in the Venn diagram in Figure S5. Of the 1,600 genes common to HmSP and the Exosome DBs, 740 or 46% are also found in the two HD datasets, with 202 found in both. An additional 520 genes from the Exosome DB intersect the HD datasets but not the HmSP DB. Thus, a total of 2,120 genes from the Exosome DB, or 47% of the entire 4,469 set are found in common with the HmSP and/or HD datasets. Moreover, all the HD subsets show a relative increase in % overlap comparing the HmSP DB with the HmSP-Exosome Common set. For example, 11% of the HmSP DB overlaps the BACHD PPI, as compared to 21% of the HmSP-Exosome Common set (Table S8), and this holds true for all of the HD subsets. Thus, the convergence of the HD and exosome datasets on the HmSP functional gene set is extended to the interactomes of the member genes of the latter. We therefore extended the analysis to the subsets of the HmSP DB.

We segregated the HmSP DB set into its component subsets: presynaptic, retrograde signaling, downscaling, upscaling, transcription regulation (presynaptic), TNF upscaling, RA upscaling, and the CAMKII/DBN sets, and also each in common with the Exosome DB, for enrichment analysis against the HD datasets (Table S9A). These results are consistent with that of the HmSP DB. In the HTT Interactome, the overlap ranges from a low of 23% of the presynaptic transcription set to a high of 40% for the CAMKII/DBN set (see % Set HmSP column), with the rest in the mid 20s to 30% range, and –log FDR values ranging from ~39 to ~109. Again, the genes found in both the HmSP subset with the Exosome DB show relatively higher % overlap in each case (compare each HmSP subset with the corresponding +Exosome subset). For example, the downscaling set overlap of 27% and –log FDR ~109 is increased to 45% and –log FDR ~140 when only those in common with the Exosome DB (+Exosome set). This pattern of increased overlap for the HmSP-Exosome Common set holds true also for PerturbDB and the HD Common set (Table S9A). Finally, the HD Common set also shows the increased relative % overlap compared to each HD dataset alone for each of the HmSP and HmSP+Exosome sets (Table S9B).

1. **Overlap of NeuroD PPIs with the HD datasets**

We examined the intersection of each of the NeuroD PPI sets with subsets of the HTT Interactome (Table S10). Generally the BACHD and CAG140 and the literature curated HIPPIE-IPA PPI sets are the most highly intersecting subsets for each of the NeuroD PPI sets (SMA not shown), with FDR –log values well over 10, except that of the Striatal cell line set aside from PD. Thus, the overlap of the HTT Interactome with the NeuroD PPIs appears to be concentrated in the proteins identified from brain and neuronal sources, supporting the relevance of these overlaps of protein networks in different CNS disorders. For PerturbDB, the mammalian mHTT aggregation platform consistently shows the highest and most significant overlap with each of the NeuroD sets, followed by the *Drosophila in vivo* phenotypes and the mammalian cell culture toxicity platforms. This suggests that protein aggregation and processing as part of pathophysiology are more likely to underlie multiple neurodegenerative diseases. Interestingly, the *C. elegans in vivo* polyQ aggregation platform also shows high overlap with the PD and ALS sets, consistent with importance of protein folding as a general mechanism important for these pathologies. The other model organisms, however, do not show FDR –log values over 10 against any of the NeuroD PPI, except for the *Drosophila* cell culture mHTT aggregation overlapping with the PD and PolyQ sets (Table S10A). Thus, the same 3 platforms we highlighted earlier as being highly intersected with each other and with the Exosome and HmSP DB also highly overlaps the NeuroD PPI sets, suggesting that these HD modifiers may be functioning in similar signaling and regulatory pathways in these other neurodegenerative disease contexts, a prediction that can be tested in experimental platforms driven by each of the neurodegenerative disease pathogenic genes to determine if these modifiers also impact the other NeuroD phenotypes.

- 1. **The NeuroD PPIs highly intersect each other**

The PPI sets for each NeuroD: PD, AD, PolyQ, ALS and SMN, and that of the HTT Interactome, show significant intersections with each other (Table S11). The PD and ALS sets show the highest degree of overlap with at least one other NeuroD PPI, with only 28% of each set showing no overlap with any other sets (211/763 genes for PD and 89/328 genes for ALS). The other PPI sets show between 43% to 50% (SMN, HTT and PolyQ) and 70% (AD) with no overlap with any other set. The FDR –log values for the overlap between the PPI sets are all well over 10 (except for SMA-AD, at ~9), generally in the 20s-50s, with a high of FDR –log ~224 for the HTT to PD intersection. A Venn diagram representation of the intersections between the NeuroD PPI sets (PD, PolyQ, ALS and AD) is shown in Figure S6, giving another perspective on how highly intersected are the PD and ALS PPI sets. The AD PPI set is the largest set and therefore only those genes that intersect one or more of the other NeuroD sets are shown in Figure S6. When we divide the 4 NeuroD sets into those genes that are in common with the HTT Interactome vs. those that are not, and then examine the Venn diagrams of intersections of those two subsets, we see that the former has a higher degree of intersections (Figure S7A and B). Thus, there is a surprising degree of overlap between protein complexes of pathogenic neurodegenerative genes in all four NeuroD diseases, particularly for those genes that are also found in the HTT Interactome. These results suggest that there is a large protein-protein network centered on HTT that also connect to the PPIs of each of these NeuroD pathogenic genes, and that they may be involved in an area of biological functions that when disrupted can lead to a variety of distinct disease phenotypes that characterize each of these neurodegenerative diseases.

- 1. **NeuroD PPIs highly intersect HmSP and Exosome DBs**

As shown in Table 8 in the main text, each NeuroD PPI highly intersects the HmSP and Exosome DBs, and the HmSP-Exosome Common set. Note, however, that the AD and PolyQ PPIs contains genes critical for HmSP (PSEN1/2 for AD, CACNA1A for SCA6 in the PolyQ set) and the degree of overlap is therefore overestimated. When each PPI is segregated into those that overlap the HTT Interactome, the resulting NeuroD + HTT subset again intersect the HmSP and Exosome DBs with highly significant FDR values (Table S11), and with increased relative % overlap of each set when compared to the subsets containing genes that do not overlap the HTT Interactome (Figure S8A-C). Conversely, the HmSP and Exosome DB and those genes in common (HmSP-Exosome_1600) show an increased relative % overlap with the NeuroD subsets that overlap or not overlap the HTT Interactome (Figure S9). When the HmSP DB is segregated into its constituent functional subsets for enrichment analysis against the NeuroD sets each in total or its subset in common with the HTT interactome, the same trend of increased relative % overlap of NeuroD total vs. its HTT Interactome overlapping subset, and of the increased relative % overlap of the HmSP functional sets and its Exosome DB intersected subset (Table S12).

1. **Overlap between the Synaptic Localized Transcripts and HD, NeuroD, exosome, and HmSP datasets**

We examined the intersection of each of the Synaptic Localized Transcripts with subsets of the HTT Interactome and PerturbDB (Table S13). As with the NeuroD PPI sets, the BACHD and CAG140 brain sets are the most highly intersecting subsets, with –log FDR values in the high 40s. Thus, not surprisingly, the overlap of the HTT Interactome with the Synaptic Localized Transcripts is concentrated in the proteins identified from brain and neuronal sources. Within PerturbDB, the two platforms of protein aggregation show the highest and most significant overlap with the local transcript set, followed by the *Drosophila in vivo* phenotypes. Thus, the same 3 platforms we highlighted as being highly intersected with each other and with the NeuroD PPI sets show the highest intersection.

The intersection between the HD and NeuroD sets for each the Synaptic Localized Transcript set and its subsets that are found in common with HmSP DB, Exosome DB and with both are shown in Supplementary Figure S10A and B. There is a relative increase in % overlap as the transcript set is subdivided into those genes that intersect the HmSP and Exosome sets, supporting the possible functional relationship between the two mechanisms and the transcripts localized to synapses, and their potential role in HD and other neurodegenerative diseases.

**References**

Besson, M.-T., Dupont, P., Fridell, Y.-W. C., and Liévens, J.-C. (2010). Increased energy metabolism rescues glia-induced pathology in a Drosophila model of Huntington's disease. Hum Mol Genet 19, 3372–3382. doi:10.1093/hmg/ddq249.

Dupont, P., Besson, M.-T., Devaux, J., and Liévens, J.-C. (2012). Reducing canonical Wingless/Wnt signaling pathway confers protection against mutant Huntingtin toxicity in Drosophila. Neurobiol Dis 47, 237–247. doi:10.1016/j.nbd.2012.04.007.

Estrada-Sánchez, A. M., and Rebec, G. V. (2012). Corticostriatal dysfunction and glutamate transporter 1 (GLT1) in Huntington's disease: interactions between neurons and astrocytes. Basal Ganglia 2, 57–66. doi:10.1016/j.baga.2012.04.029.

Hsiao, H.-Y., and Chern, Y. (2010). Targeting glial cells to elucidate the pathogenesis of Huntington's disease. Mol Neurobiol 41, 248–255. doi:10.1007/s12035-009-8097-5.

Khalil, B., Fissi, El, N., Aouane, A., Cabirol-Pol, M.-J., Rival, T., and Liévens, J.-C. (2015). PINK1-induced mitophagy promotes neuroprotection in Huntington's disease. Cell Death Dis 6, e1617. doi:10.1038/cddis.2014.581.

Lejeune F-X, Mesrob L, Parmentier F, Bicep C, Vazquez-Manrique RP, Parker JA, et al. (2012). Large-scale functional RNAi screen in C. elegans identifies genes that regulate the dysfunction of mutant polyglutamine neurons. BMC Genomics 13:91. doi:10.1186/1471-2164-13-91.

Liévens, J.-C., Rival, T., Iché, M., Chneiweiss, H., and Birman, S. (2005). Expanded polyglutamine peptides disrupt EGF receptor signaling and glutamate transporter expression in Drosophila. Hum Mol Genet 14, 713–724. doi:10.1093/hmg/ddi067.

Liévens, J.-C., Iché, M., Laval, M., Faivre-Sarrailh, C. and Birman, S. (2008). “AKT-Sensitive or Insensitive Pathways of Toxicity in Glial Cells and Neurons in Drosophila Models of Huntington's Disease.” Human Molecular Genetics 17 (6): 882–94. doi:10.1093/hmg/ddm360.

Miller JP, Holcomb J, Al-Ramahi I, de Haro M, Gafni J, Zhang N, et al. Matrix metalloproteinases are modifiers of huntingtin proteolysis and toxicity in Huntington's disease. Neuron. 2010 Jul 29;67(2):199–212. doi:10.1016/j.neuron.2010.06.021.

Miller JP, Yates BE, Al-Ramahi I, Berman AE, Sanhueza M, Kim E, et al. A genome-scale RNA-interference screen identifies RRAS signaling as a pathologic feature of Huntington's disease. PLoS Genet. 2012 Nov;8(11):e1003042. doi:10.1371/journal.pgen.1003042.

Nollen EAA, Garcia SM, van Haaften G, Kim S, Chavez A, Morimoto RI, et al. Genome-wide RNAi screen and in vivo protein aggregation reporters identify degradation of damaged proteins as an essential hypertonic stress response. Proc Natl Acad Sci USA. 2004 Apr 27;101(17):6403–8. doi:10.1152/ajpcell.00450.2008.

Sari, Y., Prieto, A. L., Barton, S. J., Miller, B. R., and Rebec, G. V. (2010). Ceftriaxone-induced up-regulation of cortical and striatal GLT1 in the R6/2 model of Huntington's disease. J. Biomed. Sci. 17, 62. doi:10.1186/1423-0127-17-62.

Teuling E, Bourgonje A, Veenje S, Thijssen K, de Boer J, van der Velde J, et al. Modifiers of mutant huntingtin aggregation: functional conservation of C. elegans-modifiers of polyglutamine aggregation. PLoS Curr. 2011;3:RRN1255. doi:10.1371/currents.RRN1255.

**Table S1A. PerturbDB Experimental Platforms, Perturbation, mHTT Length**

| **Experimental Platform** | **Gene Perturbation** | **mHTT** |
| --- | --- | --- |
| Brain Slices | Knockdown; Loss of function | Pure polyQ |
| *C. elegans* | Overexpress; Gain of function | Fragment: exon1 |
| Cell Culture | Knockout | Fragment: >exon1 |
| *Drosophila* | Dominant Negative | Full-length |
| Human | Constitutively Active |  |
| Mouse *In Vivo* | Protein Delivery |  |
| Rat *In Vivo* | Small molecule |  |
| Zebra Fish |  |  |
| Yeast |  |  |

**Table S1B. PerturbDB Outcome Measures for each Experimental Platform**

|  | **Brain Slices** | ***C. ele-gans*** | **Cell Culture** | ***Droso-phila*** | **Human** | **Mouse *In Vivo*** | **Rat *In Vivo*** | **Zebra Fish** | **Yeast** |
| --- | --- | --- | --- | --- | --- | --- | --- | --- | --- |
| Aggregation | ✓ | ✓ | ✓ | ✓ | ✓ | ✓ | ✓ | ✓ | ✓ |
| Degeneration/Toxicity | ✓ | ✓ | ✓ | ✓ | ✓ | ✓ | ✓ | ✓ | ✓ |
| Other | ✓ | ✓ | ✓ | ✓ | ✓ | ✓ | ✓ | ✓ |  |
| Correlative | ✓ | ✓ | ✓ | ✓ | ✓ | ✓ | ✓ | ✓ |  |
| Autophagy/Proteasome |  |  | ✓ |  |  | ✓ |  |  |  |
| Lifespan |  | ✓ |  | ✓ |  | ✓ |  |  |  |
| Motor Behavior |  | ✓ |  |  | ✓ | ✓✓✓ |  |  |  |
| Neuronal Functions |  | ✓* | ✓ |  |  |  |  |  |  |
| Synaptic Readouts | ✓ |  | ✓ |  |  | ✓ |  |  |  |
| Htt Molecular Modification |  |  | ✓✓ |  |  |  |  |  |  |
| Htt Level (mHtt clearance) |  |  | ✓ |  |  | ✓ |  |  |  |
| Transport Trafficking |  |  | ✓ |  |  |  |  |  |  |
| Body Weight |  |  |  |  |  | ✓ |  |  |  |
| Cognitive |  |  |  |  |  | ✓✓* |  |  |  |
| MRI |  |  |  |  |  | ✓ |  |  |  |
| Neuro Score |  |  |  |  |  | ✓ |  |  |  |
| ✓* Touch Sensitivity, neuronal degeneration | | | |  |  |  |  |  |  |
| ✓✓ Post-Translational\|Subcellular Localization | | | | |  |  |  |  |  |
| ✓✓* Fear Conditioning\|Two Choice Swim | | | |  |  |  |  |  |  |
| ✓✓✓ Grip Strength\|Rearing/Climbing\|Rotarod\|Open Field (Ambulatory Distance\|Center Rearing) | | | | | | | | | |
| Other: outcome measures not tagged in database but entered in Comments text field for queries | | | | | | | | | |
|  |  |  |  |  |  |  |  |  |  |

| **Table S2A. Gene Counts and Overlap of Experimental Platforms in PerturbDB** | | | | | | | | | | |
| --- | --- | --- | --- | --- | --- | --- | --- | --- | --- | --- |
|  |  | ***Dme in vivo* pheno** | ***Dme* cell culture aggreg** | ***Cel in vivo* pheno** | ***Cel in vivo* aggreg** | **Mamm mHTT aggreg** | **Mamm cell toxicity** | **HTT Inter-actome** | **% Set** | **Over-lap >2** |
| ***Dme in vivo* pheno_243** | **n gene** | ***147*** | **24** | **17** | **9** | **46** | **57** | **123** | 51% | 42 |
|  | **FDR** |  | *5.4E-19* | *1.5E-05* | *>E-03* | *3.8E-43* | *1.3E-42* | *7.6E-66* |  |  |
| ***Dme* cell culture aggreg_138** | **n gene** |  | ***91*** | **7** | **15** | **11** | **13** | **37** | 27% | 16 |
|  | **FDR** |  |  | *>E-03* | *1.6E-10* | *4.5E-06* | *2.2E-05* | *3.6E-10* |  |  |
| ***Cel in vivo* pheno_344** | **n gene** |  |  | ***280*** | **23** | **15** | **26** | **55** | 16% | 17 |
|  | **FDR** |  |  |  | *1.1E-12* | *2.1E-05* | *2.6E-09* | *3.2E-06* |  |  |
| ***Cel in vivo* polyQ aggreg_173** | **n gene** |  |  |  | ***108*** | **32** | **15** | **79** | 46% | 19 |
|  | **FDR** |  |  |  |  | *1.2E-29* | *3.5E-06* | *1.4E-39* |  |  |
| **Mamm mHTT aggreg_190** | **n gene** |  |  |  |  | ***66*** | **83** | **90** | 47% | 46 |
|  | **FDR** |  |  |  |  |  | *1.4E-94* | *4.5E-47* |  |  |
| **Mamm cell toxicity_336** | **n gene** |  |  |  |  |  | ***206*** | **110** | 33% | 46 |
|  | **FDR** |  |  |  |  |  |  | *1.1E-38* |  |  |
| **PerturbDB_1218** | **n gene** |  |  |  |  |  |  | **357** | 29% |  |
|  | **FDR** |  |  |  |  |  |  | *2.5E-131* |  |  |

pheno: phenotypes; aggreg: aggregation; Mamm: mammalian

Number in Bold italics where column and row headings are the same: # genes in each platform with no overlap with any other platform

Overlap between any two platforms: n gene is # of overlapping genes, in italics the FDR of the overlap

% Set: % of each platform that overlaps the HTT Interactome

Overlap >2: number of genes in indicated platform in common with 2 or more other platforms

Number of genes in each platform indicated after underscore following the platform name

| **Table S2B. Intersection between Datasets in the HTT Interactome** | | | | | | | | | |
| --- | --- | --- | --- | --- | --- | --- | --- | --- | --- |
|  |  | **CAG140** | **BACHD** | **Striatal Cell Line** | **Cell- Tissue** | **HIPPIE_IPA** | **PerturbDB** | **% Set** | **Over-lap >2** |
| **CAG140 brain _518** | **n gene** | ***298*** | 167 | 49 | 39 | 39 | 103 | 20% | 70 |
|  | **FDR** |  | *2.3E-119* | *5.9E-25* | *9.3E-22* | *1.2E-16* | *2.4E-31* |  |  |
| **BACHD brain _741** | **n gene** |  | ***417*** | 53 | 79 | 103 | 189 | 26% | 74 |
|  | **FDR** |  |  | *1.6E-21* | *4.8E-58* | *4.6E-73* | *2.3E-75* |  |  |
| **Striatal Cell Line _318** | **n gene** |  |  | ***223*** | 15 | 3 | 48 | 15% | 29 |
|  | **FDR** |  |  |  | *2.1E-05* | *2.7E-05* | *1.5E-09* |  |  |
| **Cell-Tissue Pull Down_Y2H_225** | **n gene** |  |  |  | ***107*** | 25 | 70 | 31% | 35 |
|  | **FDR** |  |  |  |  | *2.0E-14* | *1.7E-33* |  |  |
| **HIPPIE_IPA_309** | **n gene** |  |  |  |  | ***165*** | 122 | 40% | 32 |
|  | **FDR** |  |  |  |  |  | *2.8E-71* |  |  |

CAG140 brain: cytoplasmic, membrane bound fractions, HTT affinity purification (Culver et al., 2012)

BACHD brain: regions and age, HTT affinity purification (Shirasaki et al., 2012)

Striatal cells: TAP-iTRAQ of mHTT and wt HTT in striatal cell line (Ratovitski et al., 2012)

Cells-Tissue_Y2H: pull-down of HTT fragment; Y2H screens (Kaltenbach et al., 2007)

HIPPIE_IPA (Literature): Y2H screen, other interactors reported in literature

Number in Bold italics where column and row headings are the same: # genes in each platform with no overlap with any other interactome

% Set: % of each HTT Interactome Set that overlaps with the PerturbDB

Overlap >2: number of genes in indicated HTT PPI Set in common with 2 or more other Sets

Number of genes in each set indicated after underscore

| **Table S3. Overlap of HD Datasets with SynapseDB and Postsynaptic Subset** | | | | | | | | |
| --- | --- | --- | --- | --- | --- | --- | --- | --- |
|  | **SynapseDB_3549** | | |  | **Postsynaptic_1097** | | |  |
|  | n common | FDR | % Set HD | % Set SynDB. | n common | FDR | % Set HD | % Set Postsyn. |
| **HTT Interactomes_1619** | **842** | ***3.8E-280*** | **52%** | **24%** | **539** | ***< E-300*** | **33%** | **49%** |
| BACHD brain_741 | 506 | *4.2E-238* | 68% | 14% | 350 | *3.1E-272* | 47% | 32% |
| CAG140 brain_518 | 322 | *1.9E-130* | 62% | 9% | 253 | *4.3E-196* | 49% | 23% |
| Cell-Tissue Pull Down_Y2H_225 | 130 | *2.9E-46* | 58% | 4% | 89 | *5.0E-56* | 40% | 8% |
| HIPPIE_IPA_309 | 147 | *8.6E-39* | 48% | 4% | 76 | *1.7E-31* | 25% | 7% |
| Striatal Cell Line_318 | 109 | *5.6E-15* | 34% | 3% | 62 | *1.5E-19* | 19% | 6% |
| **PerturbDB Total_1218** | **493** | ***2.4E-101*** | **40%** | **14%** | **228** | ***5.9E-75*** | **19%** | **21%** |
| *Dme-in vivo* pheno_243 | 131 | *6.9E-41* | 54% | 4% | 79 | *2.1E-42* | 33% | 7% |
| Mamm. cell culture-toxicity_336 | 145 | *1.8E-32* | 43% | 4% | 51 | *4.3E-12* | 15% | 5% |
| Mamm. mHTT aggreg_190 | 92 | *8.0E-25* | 48% | 3% | 48 | *3.2E-20* | 25% | 4% |
| *Cel*-*in vivo* polyQ aggreg_173 | 86 | *2.3E-24* | 50% | 2% | 57 | *9.7E-31* | 33% | 5% |
| *Cel*-*in vivo* pheno_344 | 106 | *5.6E-11* | 31% | 3% | 47 | *7.3E-09* | 14% | 4% |
| *Dme-*cell culture-aggreg_138 | 44 | *>E-05* | 32% | 1% | 14 | *>E-05* | 10% | 1% |
| **HD Common_357** | **233** | ***4.2E-100*** | **65%** | **7%** | **155** | ***3.5E-109*** | **44%** | **14%** |

| **Table S4. Subset of PerturbDB + HTT Interactome linked to "Vesicle Dynamics"** | | | | | | | | | |
| --- | --- | --- | --- | --- | --- | --- | --- | --- | --- |
|  | **Gene ID** | **Symbol** | **Curation Tag** | ***Dme in vivo pheno*** | ***Cel* *in vivo* pheno** | ***Cel* pure polyQ Aggreg** | **Mamm mHTT Aggreg** | **Mamm Cell Toxicity** | **HTT Inter-actome** |
| 1 | 160 | AP2A1 | endocytosis | **KD** |  |  |  |  | Yes |
| 2 | 161 | AP2A2 | endocytosis | **KD** |  |  |  |  | Yes |
| 3 | 1012 | CDH13 | endocytosis | **KD** |  |  |  |  | Yes |
| 4 | 1213 | CLTC | endocytosis | **KD** |  |  | ***KD**** | ***KD**** | Yes |
| 5 | 1759 | DNM1 | endocytosis | ***KD**** |  |  |  |  | Yes |
| 6 | 3092 | HIP1 | endocytosis |  | ***KD**** |  |  |  | Yes |
| 7 | 6453 | ITSN1 | endocytosis | ***OE**** |  |  | ***OE**** |  | Yes |
| 8 | 29993 | PACSIN1 | endocytosis | **OE** |  |  |  |  | Yes |
| 9 | 11252 | PACSIN2 | endocytosis | **OE** |  |  |  |  | Yes |
| 10 | 8301 | PICALM | endocytosis | ***KD**** |  |  |  |  | Yes |
| 11 | 8766 | RAB11A | endocytosis | **OE** |  |  |  | **OE** | Yes |
| 12 | 5868 | RAB5A | endocytosis | **OE** |  | ***KD**** | **OE** | **OE** | Yes |
| 13 | 6455 | SH3GL1 | endocytosis |  | **KD** |  |  |  | Yes |
| 14 | 6457 | SH3GL3 | endocytosis |  | **KD** |  |  |  | Yes |
| 15 | 9892 | SNAP91 | endocytosis |  | ***KD**** |  |  |  | Yes |
| 16 | 8867 | SYNJ1 | endocytosis |  | ***KD**** |  |  |  | Yes |
| 17 | 128866 | CHMP4B | ESCRT-III/sorting | **KD** |  | ***KD**** |  |  | Yes |
| 18 | 8943 | AP3D1 | Vesicle Dynamics | **KD** |  |  |  |  | Yes |
| 19 | 535 | ATP6V0A1 | Vesicle Acidification |  | **KD** |  |  |  | Yes |
| 20 | 523 | ATP6V1A | Vesicle Acidification |  |  | ***KD**** |  |  | Yes |
| 21 | 529 | ATP6V1E1 | Vesicle Acidification |  |  | ***KD**** |  |  | Yes |
| 22 | 7415 | VCP | Vesicle Dynamics | **KD** |  | ***KD**** | ***KD**** |  | Yes |
| 23 | 55737 | VPS35 | Vesicle Dynamics | **KD** |  |  |  |  | Yes |
| 24 | 8775 | NAPA | Vesicular fusion | **KD** |  | ***KD**** |  |  | Yes |
| 25 | 6616 | SNAP25 | Vesicular fusion | **KD** | **KD** |  |  |  | Yes |
| 26 | 6804 | STX1A | Vesicular fusion | **KD** | **KD** |  |  |  | Yes |
| 27 | 6812 | STXBP1 | Vesicular fusion | **KD** |  |  |  |  | Yes |

pheno: phenotypes; Aggreg: aggregation; Mamm: mammalian

KD: Knockdown or loss of function ameliorated HD phenotypes

KD*: Knockdown or loss of function worsened HD phenotypes

OE: Overexpression or gain of function ameliorated HD phenotypes

OE*: Overexpression or gain of function worsened HD phenotypes

| **Table S5. Overlap of HD Data Subsets with Exosome Datasets** | | | | | | | | |  | |  |
| --- | --- | --- | --- | --- | --- | --- | --- | --- | --- | --- | --- |
|  | **Exosome Synaptic PPI_892** | | | **Exosome ProteinDB_4019** | | | **Exosome CSF Protein_739** | | | | |
|  | n common | FDR | % Set HD | n common | FDR | % Set HD | n common | FDR | | % Set HD | |
| **HTT Interactomes_1619** | ***295*** | ***2.1E-112*** | ***18%*** | ***832*** | ***2.4E-228*** | ***51%*** | ***186*** | ***1.8E-53*** | | ***12%*** | |
| BACHD brain_741 | 170 | ***3.7E-75*** | 23% | 472 | ***1.7E-175*** | 64% | 118 | ***1.0E-46*** | | 16% | |
| CAG140 brain_518 | 103 | ***1.5E-38*** | 20% | 316 | ***5.4E-108*** | 61% | 61 | ***6.2E-17*** | | 12% | |
| Cell-Tissue Pull Down_Y2H_225 | 52 | ***6.5E-23*** | 23% | 124 | ***4.9E-35*** | 55% | 45 | ***3.4E-21*** | | 20% | |
| HIPPIE_IPA_309 | 86 | ***2.7E-43*** | 28% | 125 | ***1.0E-19*** | 41% | 27 | *>E-05* | | 9% | |
| Striatal Cell Line_318 | 46 | ***7.5E-11*** | 14% | 141 | ***2.0E-26*** | 44% | 35 | *2.8E-09* | | 11% | |
| **PerturbDB_1218** | ***203*** | ***1.5E-62*** | ***17%*** | ***512*** | ***2.9E-92*** | ***42%*** | ***103*** | ***5.5E-18*** | | ***8%*** | |
| *Dme-in vivo* phenotypes_243 | 61 | ***4.3E-26*** | 25% | 127 | ***1.2E-52*** | 52% | 38 | ***2.3E-14*** | | 16% | |
| Mamm cell culture-toxicity_335 | 60 | ***2.9E-18*** | 18% | 128 | ***7.6E-32*** | 38% | 35 | *2.0E-08* | | 10% | |
| Mamm mHTT aggreg_190 | 52 | ***2.9E-24*** | 27% | 100 | ***5.3E-26*** | 53% | 25 | *6.7E-08* | | 13% | |
| *Cel*-*in vivo* polyQ aggreg_173 | 37 | ***7.0E-13*** | 21% | 128 | ***1.0E-17*** | 72% | 25 | *7.0E-09* | | 15% | |
| *Cel*-*in vivo* phenotypes_344 | 37 | *3.1E-05* | 11% | 126 | ***5.3E-15*** | 37% | 18 | *>E-05* | | 5% | |
| *Dme-*cell culture-aggreg_138 | 22 | *2.2E-05* | 16% | 55 | *2.7E-08* | 40% | 15 | *>E-05* | | 11% | |
| **HD Common_357** | ***117*** | ***5.9E-70*** | ***33%*** | ***222*** | ***2.7E-79*** | ***62%*** | ***68*** | ***6.6E-32*** | | ***19%*** | |

| **Table S6. Genes for Specific Forms of HmSP** | | | |
| --- | --- | --- | --- |
|  | **Gene ID** | **Symbol** | **HmSP Set Category** |
| 1 | 1969 | EPHA2 | Presynaptic: Eph Signaling to VGCCs |
| 2 | 2043 | EPHA4 | Presynaptic: Eph Signaling to VGCCs |
| 3 | 3392 | EPHB1 | Presynaptic: Eph Signaling to VGCCs |
| 4 | 3393 | EPHB2 | Presynaptic: Eph Signaling to VGCCs |
| 5 | 998 | CDC42 | Presynaptic: Eph Signaling to VGCCs |
| 6 | 773 | CACNA1A | Presynaptic: Eph Signaling to VGCCs |
| 7 | 23085 | ERC1 | Presynaptic: Eph Signaling to VGCCs |
| 8 | 26059 | ERC2 | Presynaptic: Eph Signaling to VGCCs |
| 9 | 84062 | DTNBP1 | Presynaptic: Regulation of RRP |
| 10 | 6616 | SNAP25 | Presynaptic: Regulation of RRP |
| 11 | 23557 | SNAPIN | Presynaptic: Regulation of RRP |
| 12 | 25782 | RAB3GAP2 | Presynaptic: Regulation of RRP |
| 13 | 22999 | RIMS1 | Presynaptic: Regulation of RRP |
| 14 | 6606 | SMN1 | Transcriptional regulation in presynaptic HmSP |
| 15 | 6607 | SMN2 | Transcriptional regulation in presynaptic HmSP |
| 16 | 5077 | PAX3 | Transcriptional regulation in presynaptic HmSP |
| 17 | 5081 | PAX7 | Transcriptional regulation in presynaptic HmSP |
| 18 | 6767 | SMAD1 | Transcriptional regulation in presynaptic HmSP |
| 19 | 6774 | SMAD9 | Transcriptional regulation in presynaptic HmSP |
| 20 | 6218 | KCNA1 | Transcriptional regulation in presynaptic HmSP |
| 21 | 6220 | KCNA2 | Transcriptional regulation in presynaptic HmSP |
| 22 | 6221 | KCNA3 | Transcriptional regulation in presynaptic HmSP |
| 23 | 3750 | KCND1 | Transcriptional regulation in presynaptic HmSP |
| 24 | 3751 | KCND2 | Transcriptional regulation in presynaptic HmSP |
| 25 | 3752 | KCND3 | Transcriptional regulation in presynaptic HmSP |
| 26 | 1978 | EIF4EBP1 | Retrograde Signaling in presynaptic HmSP |
| 27 | 1979 | EIF4EBP2 | Retrograde Signaling in presynaptic HmSP |
| 28 | 1977 | EIF4E | Retrograde Signaling in presynaptic HmSP |
| 29 | 1975 | EIF4B | Retrograde Signaling in presynaptic HmSP |
| 30 | 9670 | IPO13 | Retrograde Signaling in presynaptic HmSP |
| 31 | 2475 | MTOR | Retrograde Signaling in presynaptic HmSP |
| 32 | 6195 | RPS6KA1 | Retrograde Signaling in presynaptic HmSP |
| 33 | 6196 | RPS6KA2 | Retrograde Signaling in presynaptic HmSP |
| 34 | 6197 | RPS6KA3 | Retrograde Signaling in presynaptic HmSP |
| 35 | 90627 | STARD13 | Retrograde Signaling in presynaptic HmSP |
| 36 | 6009 | RHEB | Retrograde Signaling in presynaptic HmSP |
| 37 | 7248 | TSC1 | Retrograde Signaling in presynaptic HmSP |
| 38 | 7249 | TSC2 | Retrograde Signaling in presynaptic HmSP |
| 39 | 9229 | DLGAP1 | Postsynaptic Down Scaling |
| 40 | 1742 | DLG4 | Postsynaptic Down Scaling |
| 41 | 1020 | CDK5 | Postsynaptic Down Scaling |
| 42 | 2043 | EPHA4 | Postsynaptic Down Scaling |
| 43 | 23369 | PUM2 | Postsynaptic Down Scaling |
| 44 | 4638 | MYLK | Postsynaptic Down Scaling |
| 45 | 10769 | PLK2 | Postsynaptic Down Scaling |
| 46 | 5970 | RELA | Postsynaptic Down Scaling |
| 47 | 775 | CACNA1C | Postsynaptic Down, Up Scaling |
| 48 | 776 | CACNA1D | Postsynaptic Down, Up Scaling |
| 49 | 5504 | PPP1R2 | Postsynaptic Down, Up Scaling |
| 50 | 4204 | MECP2 | Postsynaptic Up Scaling |
| 51 | 5663 | PSEN1 | Postsynaptic Up Scaling |
| 52 | 9463 | PICK1 | Postsynaptic Up Scaling |
| 53 | 3690 | ITGB3 | Postsynaptic Up Scaling |
| 54 | 5908 | RAP1B | Postsynaptic Up Scaling |
| 55 | 1385 | CREB1 | Postsynaptic Up Scaling |
| 56 | 5530 | PPP3CA | Postsynaptic Up Scaling |
| 57 | 1432 | MAPK14 | TNF Postsynaptic Up Scaling |
| 58 | 7124 | TNF | TNF Postsynaptic Up Scaling |
| 59 | 7132 | TNFRSF1A | TNF Postsynaptic Up Scaling |
| 60 | 1605 | DAG1 | TNF Postsynaptic Up Scaling |
| 61 | 5914 | RARA | Retinoic acid postsynaptic Up Scaling |
| 62 | 2332 | FMR1 | Retinoic acid postsynaptic Up Scaling |
| 63 | 815 | CAMK2A | Calcium signaling in HmSP |
| 64 | 816 | CAMK2B | Calcium signaling in HmSP |
| 65 | 1627 | DBN1 | Calcium signaling in HmSP |

| **Table S7. Overlap between PPI Sets of specific forms of HmSP** | | | | | | | | | | | | |  |
| --- | --- | --- | --- | --- | --- | --- | --- | --- | --- | --- | --- | --- | --- |
|  | **# genes** | **Presynaptic** | **Retro-grade Sig.** | **Down scaling** | **Up scaling** | **Trans-cription** | **TNF Up-scaling** | **RA Up-scaling** | **CAMKII-DBN** | | **Over-lap >2** | | **Unique % Set** |
| **Presynaptic** | 875 | ***388*** | 151 | 245 | 184 | 93 | 123 | 50 | 106 | | 168 | | 44% |
| **Retrograde Sig.** | 752 |  | ***267*** | 234 | 175 | 110 | 178 | 98 | 77 | | 281 | | 36% |
| **Down Scaling** | 1282 |  |  | ***611*** | 243 | 140 | 206 | 136 | 116 | | 351 | | 48% |
| **Up Scaling** | 881 |  |  |  | ***383*** | 111 | 133 | 78 | 104 | | 270 | | 43% |
| **Transcription** | 684 |  |  |  |  | ***345*** | 90 | 70 | 46 | | 168 | | 50% |
| **TNF Up Scaling** | 686 |  |  |  |  |  | ***287*** | 69 | 60 | | 235 | | 42% |
| **RA Up Scaling** | 365 |  |  |  |  |  |  | ***129*** | 31 | | 136 | | 35% |
| **CAMKII-DBN** | 319 |  |  |  |  |  |  |  | ***98*** | | 142 | | 31% |
| **Unique % Set** |  | **44%** | **36%** | **48%** | **43%** | **50%** | **42%** | **35%** | **31%** | |  | |  |
| Bold: # genes in each set with no overlap with any other set | | | | | | | |  |  | |  | |  |
| Overlap >2: number of genes in indicated set in common with 2 or more other sets | | | | | | | | | | | | | |
| Unique % Set: % of genes of each set not in common with any other set | | | | | | | | | |  | |  | |

| **Table S8. Overlap of HD Datasets with HmSP and Exosome DBs** | | | | | | |  |  |
| --- | --- | --- | --- | --- | --- | --- | --- | --- |
|  | **HmSP DB_3782** | | |  | **HmSP-Exosome Common_1600** | | | |
|  | n common | FDR | % Set HD | % HmSP | n common | FDR | % Set HD | % HmSP-Exo |
| **HTT Interactomes_1619** | 825 | ***5.6E-243*** | **51%** | **22%** | 586 | ***1.3E-287*** | **36%** | **37%** |
| BACHD brain_741 | 429 | *6.4E-147* | 58% | 11% | 343 | *2.1E-199* | 46% | 21% |
| CAG140 brain_518 | 296 | *8.8E-98* | 57% | 8% | 237 | *4.0E-133* | 46% | 15% |
| Cell-Tissue Pull Down_Y2H_225 | 194 | *9.0E-73* | 63% | 5% | 129 | *1.0E-64* | 42% | 8% |
| HIPPIE_IPA_309 | 108 | *4.1E-26* | 48% | 3% | 82 | *1.7E-35* | 36% | 5% |
| Striatal Cell Line_318 | 137 | *8.3E-27* | 43% | 4% | 95 | *1.5E-32* | 30% | 6% |
| **PerturbDB Total_1218** | 583 | ***1.7E-151*** | **48%** | **16%** | 356 | ***3.3E-132*** | **29%** | **22%** |
| *Dme-in vivo* phenotypes_243 | 143 | *6.1E-48* | 59% | 4% | 96 | *1.5E-44* | 39% | 6% |
| Mamm cell culture-toxicity_336 | 185 | *3.2E-55* | 55% | 5% | 104 | *4.8E-37* | 31% | 6% |
| Mamm mHTT aggreg_190 | 125 | *1.6E-49* | 66% | 3% | 91 | *4.1E-51* | 48% | 6% |
| *Cel*-*in vivo* polyQ aggreg_173 | 96 | *1.2E-29* | 56% | 3% | 79 | *1.6E-42* | 46% | 5% |
| *Cel*-*in vivo* phenotypes_344 | 124 | *3.3E-16* | 36% | 3% | 70 | *1.0E-13* | 21% | 4% |
| *Dme-*cell culture-aggreg_138 | 63 | *2.5E-13* | 46% | 2% | 43 | *6.6E-15* | 31% | 3% |
| **HD Common_357** | 254 | ***1.9E-117*** | **71%** | **7%** | 202 | ***4.3E-139*** | **57%** | **13%** |

| **Table S9A. Overlap of HmSP and HmSP-Exosome Common with HD Datasets** | | | | | | | | | |
| --- | --- | --- | --- | --- | --- | --- | --- | --- | --- |
|  | **HTT Interactome_1619** | | | **PerturbDB_1218** | | | **HD Common_357** | | |
|  | n common | FDR | % Set HmSP | n common | FDR | % Set HmSP | n common | FDR | % Set HmSP |
| **Presynaptic_875** | 275 | *6.9E-106* | 31% | 159 | *1.5E-42* | 18% | 91 | *9.2E-48* | 11% |
| **Presynaptic+Exosome_480** | 204 | *1.3E-105* | **43%** | 114 | *6.1E-42* | **24%** | 78 | *2.3E-55* | **17%** |
| **Retrograde Sig_752** | 244 | *1.3E-96* | 32% | 194 | *3.2E-78* | 26% | 116 | *1.3E-80* | 15% |
| **Retrograde Sig+Exosome_418** | 199 | *1.8E-114* | **48%** | 145 | *1.5E-76* | **35%** | 102 | *2.9E-91* | **24%** |
| **Up Scaling_881** | 247 | *1.2E-82* | 28% | 183 | *1.8E-58* | 21% | 96 | *6.6E-32* | 11% |
| **Up Scaling+Exosome_451** | 183 | *3.3E-90* | **41%** | 121 | *1.2E-50* | **27%** | 76 | *4.0E-55* | **17%** |
| **Down Scaling_1282** | 340 | *7.0E-109* | 27% | 247 | *5.5E-74* | 19% | 133 | *1.7E-73* | 11% |
| **Down Scaling+Exosome_559** | 253 | *2.3E-140* | **45%** | 167 | *3.8E-79* | **30%** | 113 | *1.0E-94* | **21%** |
| **Transcription_684** | 157 | *1.5E-39* | 23% | 125 | *6.1E-33* | 18% | 57 | *1.1E-23* | 8% |
| **Transcription+Exosome_279** | 103 | *1.2E-45* | **37%** | 73 | *4.1E-29* | **26%** | 43 | *2.7E-28* | **15%** |
| **TNF Up Scaling_686** | 173 | *9.6E-50* | 25% | 134 | *6.3E-40* | 20% | 73 | *1.4E-38* | 11% |
| **TNF Up Scaling+Exosome_368** | 148 | *6.9E-72* | **40%** | 94 | *2.1E-37* | **26%** | 62 | *9.3E-45* | **17%** |
| **CAMKII-DBN_319** | 126 | *6.2E-60* | 40% | 83 | *6.5E-33* | 26% | 51 | *1.8E-34* | 16% |
| **RA Up Scaling_365** | 116 | *8.14E-44* | 32% | 94 | *1.6E-38* | 26% | 52 | *8.8E-35* | 15% |

| **Table S9B. Relative % overlap of HD Datasets** | | | |
| --- | --- | --- | --- |
|  | **% HTT Interactome** | **% PerturbDB** | **% HD Common** |
| **Presynaptic_875** | 17% | 13% | **25%** |
| **Presynaptic+Exosome_480** | 13% | 9% | **22%** |
| **Retrograde Sig_752** | 15% | 16% | **32%** |
| **Retrograde Sig+Exosome_418** | 12% | 12% | **29%** |
| **Up Scaling_881** | 15% | 15% | **27%** |
| **Up Scaling+Exosome_451** | 11% | 10% | **21%** |
| **Down Scaling_1282** | 21% | 20% | **37%** |
| **Down Scaling+Exosome_559** | 16% | 14% | **32%** |
| **Transcription_684** | 10% | 10% | **16%** |
| **Transcription+Exosome_279** | 6% | 6% | **12%** |
| **TNF Up Scaling_686** | 11% | 11% | **20%** |
| **TNF Up Scaling+Exosome_368** | 9% | 8% | **17%** |
| **CAMKII-DBN_319** | 8% | 7% | **14%** |
| **RA Up Scaling_365** | 7% | 8% | **15%** |

| **Table S10A. Overlap of HD and NeuroD Datasets** | | | | | |  |
| --- | --- | --- | --- | --- | --- | --- |
|  | **PD_PPI_763** | |  | **PolyQ_PPI_1139** | | |
|  | n common | FDR | % Set HD | n common | FDR | % Set HD |
| **HTT Interactomes_1619** | 370 | ***5.4E-224*** | **23%** | 303 | ***2.1E-96*** | **19%** |
| BACHD brain_741 | 223 | ***8.3E-153*** | 30% | 145 | ***1.1E-45*** | 20% |
| CAG140 brain_518 | 89 | ***1.5E-55*** | 31% | 113 | ***7.0E-65*** | 19% |
| HIPPIE_IPA_309 | 158 | ***3.1E-106*** | 29% | 100 | ***1.7E-30*** | 37% |
| Cell-Tissue Pull Down_Y2H_225 | 69 | ***8.8E-45*** | 31% | 46 | ***1.1E-14*** | 20% |
| Striatal Cell Line_318 | 47 | ***1.2E-15*** | 15% | 37 | *4.6E-05* | 12% |
| **PerturbDBl_1218** | 232 | ***2.5E-111*** | **19%** | 220 | ***3.2E-64*** | **18%** |
| *Dme-in vivo* phenotypes_243 | 75 | ***5.7E-49*** | 31% | 65 | ***5.6E-28*** | 27% |
| Mamm cell culture-toxicity_336 | 67 | ***1.0E-30*** | 20% | 74 | ***6.3E-26*** | 22% |
| Mamm cell culture-aggreg_190 | 68 | ***7.1E-49*** | 36% | 56 | ***4.4E-26*** | 30% |
| *Cel*-*in vivo* polyQ aggreg_173 | 48 | ***7.6E-29*** | 28% | 28 | *5.7E-07* | 16% |
| *Cel*-*in vivo* phenotypes_344 | 41 | *2.8E-10* | 12% | 49 | *3.9E-09* | 14% |
| *Dme-*cell culture-aggreg_138 | 31 | ***6.6E-15*** | 23% | 34 | ***9.0E-13*** | 25% |
| **HD Common_357** | 150 | ***5.8E-124*** | **42%** | 113 | ***3.0E-57*** | **31%** |

| **Table S10B. Overlap of HD and NeuroD Datasets** | | | | | | |
| --- | --- | --- | --- | --- | --- | --- |
|  | **AD_PPI_2354** | |  | **ALS_PPI_328** | |  |
|  | n common | FDR | % Set HD | n common | FDR | % Set HD |
| **HTT Interactomes_1619** | 400 | ***1.1E-65*** | **25%** | 123 | ***1.7E-55*** | **8%** |
| BACHD brain_741 | 206 | ***1.9E-40*** | 28% | 61 | ***2.1E-27*** | 8% |
| CAG140 brain_518 | 105 | ***7.2E-28*** | 21% | 36 | ***1.5E-20*** | 11% |
| HIPPIE_IPA_309 | 110 | ***2.3E-12*** | 34% | 56 | ***6.7E-31*** | 12% |
| Cell-Tissue Pull Down_Y2H_225 | 64 | ***1.0E-12*** | 28% | 14 | *8.5E-05* | 6% |
| Striatal Cell Line_318 | 72 | *6.1E-09* | 23% | 21 | *3.6E-07* | 7% |
| **PerturbDBl_1218** | 280 | ***4.2E-38*** | **23%** | 80 | ***1.2E-29*** | **7%** |
| *Dme-in vivo* phenotypes_243 | 75 | ***2.4E-17*** | 31% | 22 | *1.7E-10* | 9% |
| Mamm cell culture-toxicity_336 | 99 | ***4.9E-21*** | 30% | 28 | ***2.4E-12*** | 8% |
| Mamm cell culture-aggreg_190 | 74 | ***1.4E-23*** | 39% | 30 | ***9.7E-21*** | 16% |
| *Cel*-*in vivo* polyQ aggreg_173 | 41 | *4.0E-06* | 24% | 20 | ***1.9E-11*** | 12% |
| *Cel*-*in vivo* phenotypes_344 | 65 | *4.2E-05* | 19% | 18 | *>E-05* | 5% |
| *Dme-*cell culture-aggreg_138 | 24 | *>E-05* | 17% | 10 | *>E-05* | 7% |
| **HD Common_357** | 127 | ***6.2E-36*** | **35%** | 47 | ***1.5E-29*** | **13%** |

| **Table S11. Intersections Between NeuroD PPIs** | | | | | | |  |  |
| --- | --- | --- | --- | --- | --- | --- | --- | --- |
|  |  | **HTT PPI_1619** | **PD PPI_763** | **AD PPI_2354** | **ALS PPI_328** | **PolyQ PPI_1139** | **SMA PPI_241** | **Overlap >2** |
| **HTT PPI_1619** | n common | ***789*** | 370 | 400 | 123 | 303 | 67 | 304 |
|  | FDR |  | *5.4E-224* | *1.1E-65* | *1.7E-55* | *2.1E-96* | *1.1E-21* |  |
| **PD PPI_763** | n gene |  | ***211*** | 247 | 87 | 196 | 43 | 268 |
|  | FDR |  |  | *1.0E-62* | *1.2E-51* | *1.9E-84* | *2.7E-18* |  |
| **AD PPI_2354** | n gene |  |  | ***1651*** | 99 | 276 | 59 | 258 |
|  | FDR |  |  |  | *6.8E-22* | *7.2E-42* | *2.4E-09* |  |
| **ALS PPI_328** | n gene |  |  |  | ***89*** | 103 | 43 | 125 |
|  | FDR |  |  |  |  | *1.0E-51* | *2.2E-32* |  |
| **PolyQ PPI_1139** | n gene |  |  |  |  | ***567*** | 48 | 241 |
|  | FDR |  |  |  |  |  | *1.9E-15* |  |
| **SMA PPI_241** | n gene |  |  |  |  |  | ***103*** | 70 |
| **Unique genes**  **% Set** | | **49%** | **28%** | **70%** | **27%** | **50%** | **43%** |  |
| # in bold italics: # genes in each PPI set with no overlap with any other PPI set | | | | | | | |  |
| Overlap: in bold the # of genes in common, in italics the FDR of the overlap | | | | | | | |  |
| Overlap >2: number of genes in indicated PPI set in common with 2 or more other PPI sets | | | | | | | | |
| Unique genes % Set: % of genes of each set not in common with any other set | | | | | | | | |
| Number of genes in each set indicated after underscore | | | | | |  |  |  |

| **Table S12. Overlap of NeuroD +HTT PPIs with HmSP and Exosome DBs** | | | | | | |
| --- | --- | --- | --- | --- | --- | --- |
|  | **HmSP DB_3782** | | **Exosomes DB_4469** | | **HmSP Exosome Common_1600** | |
|  | n common | FDR | n common | FDR | n common | FDR |
| **PD +HTT PPI_371** | 277 | ***2.1E-135*** | 299 | ***5.0E-142*** | 235 | ***7.9E-176*** |
| **PolyQ +HTT PPI_303** | 252 | ***8.5E-143*** | 194 | ***9.3E-63*** | 175 | ***2.4E-119*** |
| **AD +HTT PPI_400** | 266 | ***1.0E-109*** | 273 | ***9.0E-99*** | 207 | ***4.7E-129*** |
| **ALS +HTT PPI_123** | 106 | ***2.8E-62*** | 104 | ***3.1E-52*** | 91 | ***1.7E-75*** |

| **Table S13A. Enrichment Analysis of NeuroD Sets with HmSP Subsets** | | | | | | | | |  |  |  |  |
| --- | --- | --- | --- | --- | --- | --- | --- | --- | --- | --- | --- | --- |
|  | **A: Presynaptic_875** | | | | | **B: Presynaptic+EV_480** | | | | | **% Set HmSP** | |
|  | n common | FDR | | % Set NeuroD | | n common | | FDR | | % Set NeuroD | A | B |
| PD Total PPI_763 | 173 | ***1.4E-83*** | | 23% | | 141 | | ***8.1E-92*** | | 18% | 20% | 29% |
| PD +HTT PPI_371 | 120 | ***7.4E-76*** | | 32% | | 104 | | ***7.0E-86*** | | 28% | 14% | 22% |
| PolyQ Total PPI_1139 | 327 | ***1.7E-204*** | | 29% | | 190 | | ***1.9E-119*** | | 17% | 37% | 40% |
| PolyQ +HTT PPI_303 | 131 | ***6.2E-102*** | | 43% | | 102 | | ***4.3E-93*** | | 34% | 15% | 21% |
| ALS Total PPI_328 | 48 | ***4.5E-14*** | | 15% | | 38 | | ***4.7E-16*** | | 12% | 5% | 8% |
| ALS +HTT PPI_123 | 26 | ***3.0E-11*** | | 21% | | 23 | | ***4.3E-14*** | | 19% | 3% | 5% |
| AD Total PPI_2354 | 238 | ***8.8E-45*** | | 10% | | 168 | | ***7.2E-47*** | | 7% | 27% | 35% |
| AD +HTT PPI_400 | 101 | ***5.3E-52*** | | 25% | | 87 | | ***7.9E-61*** | | 22% | 12% | 18% |
|  |  |  | |  | |  | |  | |  |  |  |
| **Table S13B. Enrichment Analysis of NeuroD Sets with HmSP Subsets** | | | | | | | | | | |  |  |
|  | **A: Up Scaling_881** | | | | | **B: Up Scaling+EV_451** | | | | | **% Set HmSP** | |
|  | n common | FDR | | % Set NeuroD | | n common | | FDR | | % Set NeuroD | A | B |
| PD Total PPI_763 | 168 | ***2.1E-78*** | | 22% | | 132 | | ***1.9E-85*** | | 17% | 19% | 29% |
| PD +HTT PPI_371 | 98 | ***3.2E-52*** | | 26% | | 85 | | ***7.1E-64*** | | 23% | 11% | 19% |
| PolyQ Total PPI_1139 | 218 | ***5.6E-91*** | | 19% | | 118 | | ***1.7E-50*** | | 10% | 25% | 26% |
| PolyQ +HTT PPI_303 | 105 | ***3.9E-69*** | | 35% | | 78 | | ***1.7E-62*** | | 26% | 12% | 17% |
| ALS Total PPI_328 | 71 | ***3.1E-31*** | | 22% | | 49 | | ***7.2E-27*** | | 15% | 8% | 11% |
| ALS +HTT PPI_123 | 33 | ***2.1E-17*** | | 27% | | 27 | | ***4.5E-19*** | | 22% | 4% | 6% |
| AD Total PPI_2354 | 358 | ***1.2E-126*** | | 15% | | 234 | | ***1.2E-108*** | | 10% | 41% | 52% |
| AD +HTT PPI_400 | 139 | ***1.0E-92*** | | 35% | | 114 | | ***9.0E-99*** | | 29% | 16% | 25% |
|  |  |  | |  | |  | |  | |  |  |  |
| **Table S13C. Enrichment Analysis of NeuroD Sets with HmSP Subsets** | | | | | | | | | | |  |  |
|  | **A: Down Scaling_1282** | | | | | **B: Down Scaling+EV_559** | | | | | **% Set HmSP** | |
|  | n common | FDR | | % Set NeuroD | | n common | | FDR | | % Set NeuroD | A | B |
| PD Total PPI_763 | 219 | ***7.1E-95*** | | 29% | | 180 | | ***6.2E-127*** | | 24% | 17% | 32% |
| PD +HTT PPI_371 | 147 | ***3.2E-84*** | | 40% | | 134 | | ***1.1E-119*** | | 36% | 12% | 24% |
| PolyQ Total PPI_1139 | 283 | ***2.0E-107*** | | 25% | | 146 | | ***8.1E-63*** | | 13% | 22% | 26% |
| PolyQ +HTT PPI_303 | 116 | ***6.0E-64*** | | 38% | | 81 | | ***9.7E-59*** | | 27% | 9% | 15% |
| ALS Total PPI_328 | 98 | ***1.9E-42*** | | 30% | | 77 | | ***7.7E-51*** | | 23% | 8% | 14% |
| ALS +HTT PPI_123 | 55 | ***5.6E-34*** | | 45% | | 50 | | ***8.0E-46*** | | 41% | 4% | 9% |
| AD Total PPI_2354 | 316 | ***4.7E-50*** | | 13% | | 185 | | ***1.2E-47*** | | 8% | 25% | 33% |
| AD +HTT PPI_400 | 124 | ***2.3E-56*** | | 31% | | 102 | | ***1.8E-72*** | | 26% | 10% | 18% |
| **Table S13D. Enrichment Analysis of NeuroD Sets with HmSP Subsets** | | | | | | | | | | |  |  |
|  | **A: Transcription_684** | | | | | **B: Transcription+EV_279** | | | | | **% Set HmSP** | |
|  | n common | FDR | | % Set NeuroD | | n common | | FDR | | % Set NeuroD | A | B |
| PD Total PPI_763 | 104 | ***5.5E-38*** | | 14% | | 79 | | ***3.8E-49*** | | 10% | 15% | 28% |
| PD +HTT PPI_371 | 62 | ***3.7E-27*** | | 17% | | 51 | | ***7.1E-37*** | | 14% | 9% | 18% |
| PolyQ Total PPI_1139 | 160 | ***8.3E-62*** | | 14% | | 81 | | ***4.6E-38*** | | 7% | 23% | 29% |
| PolyQ +HTT PPI_303 | 61 | ***3.5E-31*** | | 20% | | 42 | | ***3.3E-30*** | | 14% | 9% | 15% |
| ALS Total PPI_328 | 73 | ***3.0E-40*** | | 22% | | 48 | | ***1.7E-35*** | | 15% | 11% | 17% |
| ALS +HTT PPI_123 | 40 | ***1.2E-28*** | | 33% | | 32 | | ***1.2E-31*** | | 26% | 6% | 12% |
| AD Total PPI_2354 | 151 | ***2.1E-18*** | | 6% | | 88 | | ***6.4E-21*** | | 4% | 22% | 32% |
| AD +HTT PPI_400 | 58 | ***4.7E-22*** | | 15% | | 45 | | ***1.9E-28*** | | 11% | 8% | 16% |
|  |  |  | |  | |  | |  | |  |  |  |
| **Table S13E. Enrichment Analysis of NeuroD Sets with HmSP Subsets** | | | | | | | | | | |  |  |
|  | **A: TNF Up Scaling_686** | | | | | **B: TNF Up Scaling+EV_368** | | | | | **% Set HmSP** | |
|  | n common | FDR | | % Set NeuroD | | n common | | FDR | | % Set NeuroD | A | B |
| PD Total PPI_763 | 150 | ***4.2E-78*** | | 20% | | 121 | | ***1.0E-84*** | | 16% | 22% | 33% |
| PD +HTT PPI_371 | 89 | ***1.1E-52*** | | 24% | | 83 | | ***4.6E-69*** | | 22% | 13% | 23% |
| PolyQ Total PPI_1139 | 145 | ***6.1E-50*** | | 13% | | 100 | | ***3.1E-44*** | | 9% | 21% | 27% |
| PolyQ +HTT PPI_303 | 67 | ***7.1E-37*** | | 22% | | 59 | | ***1.6E-44*** | | 19% | 10% | 16% |
| ALS Total PPI_328 | 70 | ***3.3E-37*** | | 21% | | 60 | | ***2.4E-43*** | | 18% | 10% | 16% |
| ALS +HTT PPI_123 | 32 | ***1.4E-19*** | | 26% | | 30 | | ***3.8E-25*** | | 24% | 5% | 8% |
| AD Total PPI_2354 | 184 | ***2.1E-33*** | | 8% | | 119 | | ***2.7E-29*** | | 5% | 27% | 32% |
| AD +HTT PPI_400 | 72 | ***1.8E-33*** | | 18% | | 65 | | ***4.7E-44*** | | 16% | 11% | 18% |
|  |  |  | |  | |  | |  | |  |  |  |
| **Table S13F. Enrichment Analysis of NeuroD Sets with HmSP Subsets** | | | | | | | | | | |  |  |
|  | **A: Retrograde Signaling_752** | | | | | **B: Retrograde Signaling+EV_418** | | | | | **% Set HmSP** | |
|  | n common | FDR | | % Set NeuroD | | n common | | FDR | | % Set NeuroD | A | B |
| PD Total PPI_763 | 185 | ***1.7E-107*** | | 24% | | 157 | | ***9.2E-122*** | | 21% | 25% | 38% |
| PD +HTT PPI_371 | 118 | ***2.4E-81*** | | 32% | | 107 | | ***6.3E-97*** | | 29% | 16% | 26% |
| PolyQ Total PPI_1139 | 185 | ***7.0E-76*** | | 16% | | 116 | | ***1.6E-52*** | | 10% | 25% | 28% |
| PolyQ +HTT PPI_303 | 88 | ***3.6E-56*** | | 29% | | 70 | | ***1.1E-54*** | | 23% | 12% | 17% |
| ALS Total PPI_328 | 95 | ***2.6E-60*** | | 29% | | 77 | | ***1.4E-60*** | | 23% | 13% | 18% |
| ALS +HTT PPI_123 | 46 | ***2.6E-34*** | | 37% | | 44 | | ***6.9E-43*** | | 36% | 6% | 11% |
| AD Total PPI_2354 | 228 | ***1.6E-51*** | | 10% | | 143 | | ***2.0E-38*** | | 6% | 30% | 34% |
| AD +HTT PPI_400 | 98 | ***3.3E-55*** | | 25% | | 77 | | ***4.9E-54*** | | 19% | 13% | 18% |
| **Table S14. Overlap of Synaptic Localized Transcripts with HD Datasets** | | | | | | | | |  |  |  |  |
|  | | | **Synaptic Localized Transcripts_2305** | | | | | |  |  |  |  |
|  | | | n common | | FDR | | % Set  HD | |  |  |  |  |
| **HTT Interactomes_1619** | | | 436 | | ***1.31E-87*** | | 27% | |  |  |  |  |
| BACHD brain_741 | | | 220 | | ***1.09E-49*** | | 30% | |  |  |  |  |
| CAG140 brain_518 | | | 174 | | ***6.28E-47*** | | 34% | |  |  |  |  |
| HIPPIE_IPA_309 | | | 78 | | ***4.68E-13*** | | 25% | |  |  |  |  |
| Cell-Tissue Pull Down_Y2H_225 | | | 58 | | *5.68E-10* | | 26% | |  |  |  |  |
| Striatal Cell Line_318 | | | 78 | | ***7.91E-12*** | | 25% | |  |  |  |  |
| **PerturbDB_1218** | | | 282 | | ***1.98E-40*** | | 23% | |  |  |  |  |
| *Dme-in vivo* phenotypes_243 | | | 62 | | ***8.68E-11*** | | 26% | |  |  |  |  |
| Mamm cell culture-toxicity_335 | | | 67 | | *5.44E-07* | | 20% | |  |  |  |  |
| Mamm mHTT aggreg_190 | | | 58 | | ***2.47E-13*** | | 31% | |  |  |  |  |
| *Cel*-*in vivo* polyQ aggreg_173 | | | 69 | | ***2.91E-23*** | | 40% | |  |  |  |  |
| *Cel*-*in vivo* phenotypes_344 | | | 60 | | *>E-05* | | 17% | |  |  |  |  |
| *Dme-*cell culture-aggreg_138 | | | 43 | | *6.37E-10* | | 31% | |  |  |  |  |
| **HD Common_357** | | | 113 | | ***1.55E-27*** | | 31% | |  |  |  |  |
