# Supplementary material for: Exosomes and Homeostatic Synaptic Plasticity Are Linked to Each other and to Huntington's, Parkinson's, and Other Neurodegenerative Diseases by Database-Enabled Analyses of Comprehensively Curated Datasets
Source: Front Neurosci. 2017 Mar 31;11:149. doi: 10.3389/fnins.2017.00149 (PMC5374209; doi:10.3389/fnins.2017.00149)
Supplement: Supplementary file 5 [file DataSheet5.docx]

**Supplementary Figure Legends**

Figure S1. Example of a gene page form from PerturbDB showing the various attributes curated from the article qualifying the gene for PerturbDB. The Entrez gene ID (Human) and PMID in which it is curated are shown. A short summary of this particular gene/PMID data is written by the curator and incorporated into the Master Data Table (Supplementary Datasheet 2). Under each Experimental Platform are the attributes such as type of perturbation and endpoints allowing for efficient querying of multiple attributes at that same time. All of the attributes and annotation shown on this form are available in Supplementary Datasheet 1.

Figure S2. Example of a gene page form from the iterative Access database integrating PubMed articles in which there are co-citations of gene IDs and MeSH terms of interest. For each gene the form displays the Entrez gene data, membership of the gene in HD datasets (PerturbDB, HTT Interactome, expression data), MeSH terms selected for this round of curation, links to other gene sets and sources that evaluate druggability. Link-outs are hyperlinks to documents showing the metadata and abstracts of articles that co-cite the gene ID and the selected MeSH headings to which the MeSH terms belong.

Figure S3. Venn Diagram of the intersections of 3 Major PerturbDB Platforms, and HTT Interactome. A: Venn diagram of the 3 major experimental platforms in PerturbDB and their intersection with each other, and the subsets that overlap with the HTT Interactome. B: As in A. but only the 64 genes found in common with the *in vivo* rodent set is plotted. The area is approximately proportional to the set size within A and separately in B, with the number of genes indicated in each area, and the number also found in the HTT Interactome (1,619 total) indicated in red. The total number of genes in each set depicted in the figure is indicated after the underscore.

Figure S4. Venn diagram of the intersections of HD datasets with the SynapseDB and its Postsynaptic subset. A: Intersections of the HTT Interactome and PerturbDB with SynapseDB. The area of the sets is approximately proportional to the set size, with the number of genes indicated. A portion of SynapseDB containing the Postsynaptic Set and the intersecting HD datasets is extracted and shown in B. B: Intersection of the HD datasets with the Postsynaptic subset extracted from the SynapseDB. The number following the underscore indicates the total size of each dataset.

Figure S5. Venn diagram of the intersections of the HmSP DB with the two HD datasets, and their overlap with the Exosome DB. The area of the sets is approximately proportional to the set size, with the number of genes indicated. The number following the underscore indicates the total size of each dataset. The 2,349 Exosome DB genes that do not overlap the other datasets are not represented.

Figure S6. A Venn diagram of the intersections of the NeuroD PPI sets for PD, PolyQ, AD, and ALS. The area of the sets is approximately proportional to the set size, with the number of genes indicated. For the AD PPI set, only those genes that intersect one or more of the other sets are shown. The number following the underscore indicates the total size of each dataset, except for the AD set where it is the number of genes that intersect, and the number in parenthesis is the total number of genes for the AD set.

Figure S7. A Venn diagram of the intersections of the NeuroD PPI sets for PD, PolyQ, AD, and ALS. The area of the sets is approximately proportional to the set size, with the number of genes indicated. For the AD PPI set, only those genes that intersect one or more of the other sets are shown. The number following the underscore indicates the total size of each dataset, except for the AD set where it is the number of genes that intersect, and the number in parenthesis is the total number of genes for the AD set.

Figure S8. Percent overlap of the NeuroD PPI sets (A. PolyQ, B. AD, and C. ALS) with the HmSP and Exosome DBs. Percent of the NeuroD PPI sets that intersect the HmSP DB, Exosome DB, and the genes found in both HmSP and Exosome DBs (HmSP-Exosome) are shown. The NeuroD PPI sets are the entire set (NeuroD Total), the subset found in common with the HTT Interactome (NeuroD +HTT), or the subset with no overlap with HTT Interactome (NeuroD no HTT). The number following the underscore indicates the size of each dataset.

Figure S9. Percent overlap of the HmSP and Exosome DBs with the NeuroD PPI subsets with (+HTT) or without (no HTT) overlap with the HTT Interactome. Percent of the HmSP and Exosome DB sets that intersect the NeuroD subsets are shown. The number following the underscore indicates the size of each dataset.

Figure S10. Percent overlap of (A) the HTT Interactome, PerturbDB, and (B) NeuroD PPI sets with the Synaptic Localized Transcripts set and its subsets found in common with HmSP and Exosome DBs and in both. Percent of the HD and NeuroD sets that intersect the Synaptic Localized Transcripts, HmSP DB, Exosome DB, and the genes found in common are shown. The number following the underscore indicates the size of each dataset.

Figure S11. Flow scheme of possible hypotheses validation experiments for exosome biology, pre- and postsynaptic HmSP mechanisms in any neurodegenerative diseases where mutant pathogenic genes can be tested for their effects vs. WT genes. If the validation experiments are affirmative, elucidating a larger set of modifiers will reveal the underlying pathophysiology, leading to development of novel mechanistic assays, target nomination and prosecution in drug discovery campaigns.
