# Supplementary material for: Exosomes and Homeostatic Synaptic Plasticity Are Linked to Each other and to Huntington's, Parkinson's, and Other Neurodegenerative Diseases by Database-Enabled Analyses of Comprehensively Curated Datasets
Source: Front Neurosci. 2017 Mar 31;11:149. doi: 10.3389/fnins.2017.00149 (PMC5374209; doi:10.3389/fnins.2017.00149)

Figure S3. Overlap of PerturbDB platforms and HTT Interactome
A: All genes; B: In common with rodent *In Vivo* platform

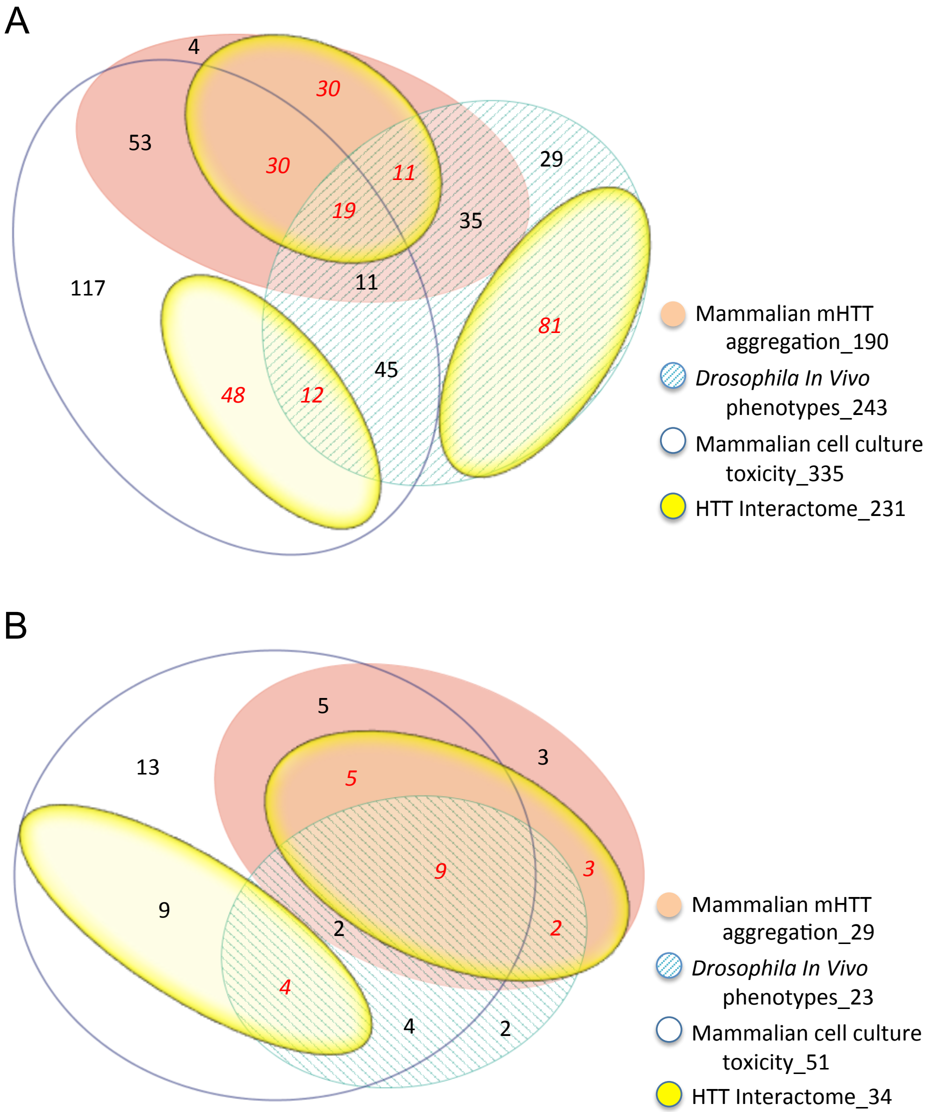

Supplement: Supplementary file 8 [file Image3.pdf]
