# Supplementary material for: Exosomes and Homeostatic Synaptic Plasticity Are Linked to Each other and to Huntington's, Parkinson's, and Other Neurodegenerative Diseases by Database-Enabled Analyses of Comprehensively Curated Datasets
Source: Front Neurosci. 2017 Mar 31;11:149. doi: 10.3389/fnins.2017.00149 (PMC5374209; doi:10.3389/fnins.2017.00149)

Figure S4. Overlap of PerturbDB and HTT Interactome with SynapseDB
A: All of SynapseDB; B: Postsynaptic subset

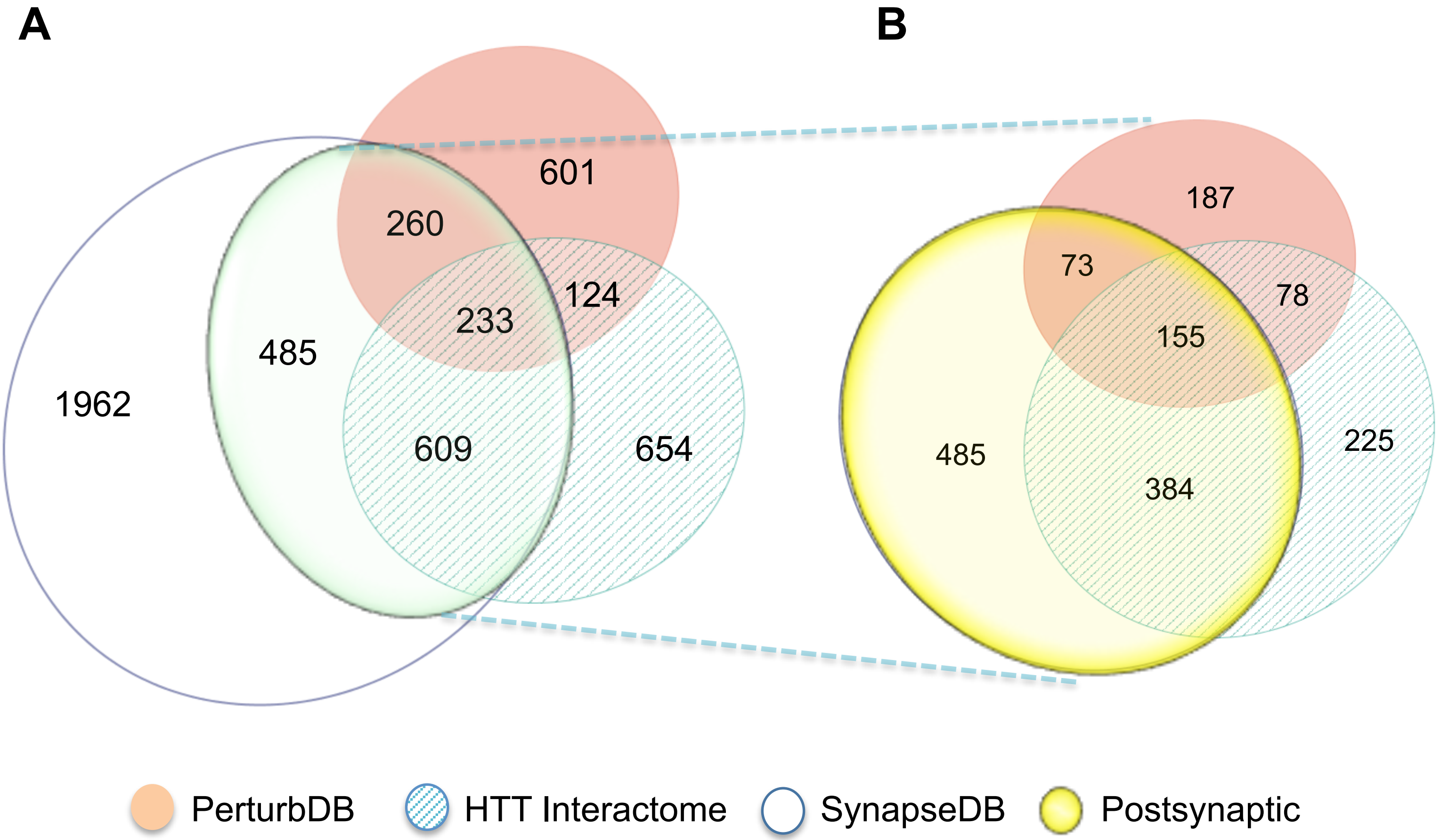

Supplement: Supplementary file 9 [file Image4.pdf]
