# Supplementary figures and images for: Exosomes and Homeostatic Synaptic Plasticity Are Linked to Each other and to Huntington's, Parkinson's, and Other Neurodegenerative Diseases by Database-Enabled Analyses of Comprehensively Curated Datasets
Source: Front Neurosci. 2017 Mar 31;11:149. doi: 10.3389/fnins.2017.00149 (PMC5374209; doi:10.3389/fnins.2017.00149)

Figure S5. Overlap of PerturbDB and HTT Interactome with HmSP and Exosome DBs

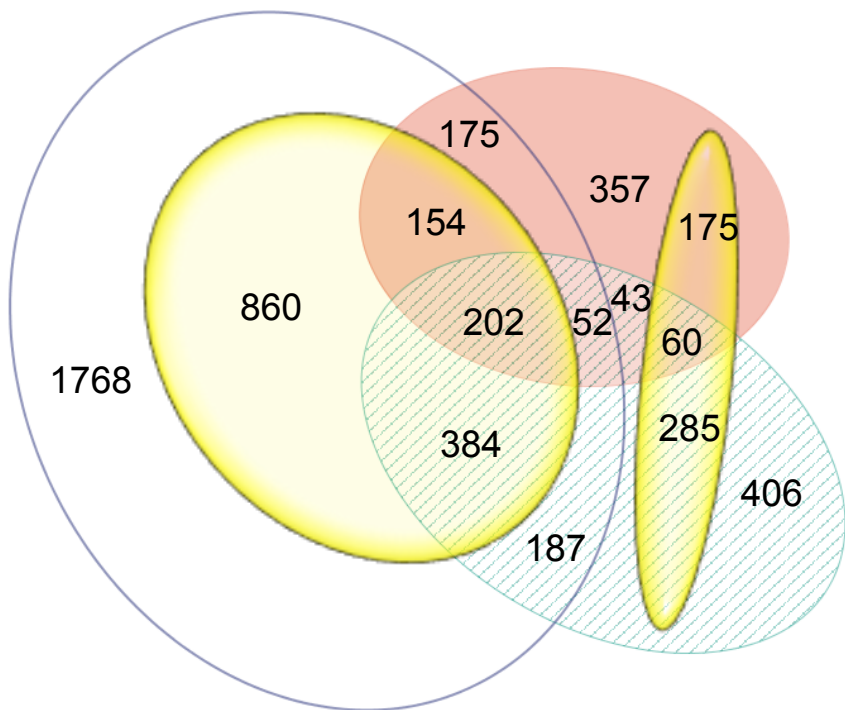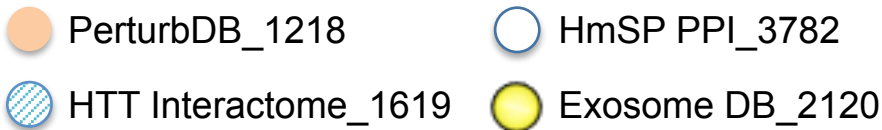

Supplement: Supplementary file 10 [file Image5.pdf]

Figure S6. Overlap of NeuroD Sets with each other

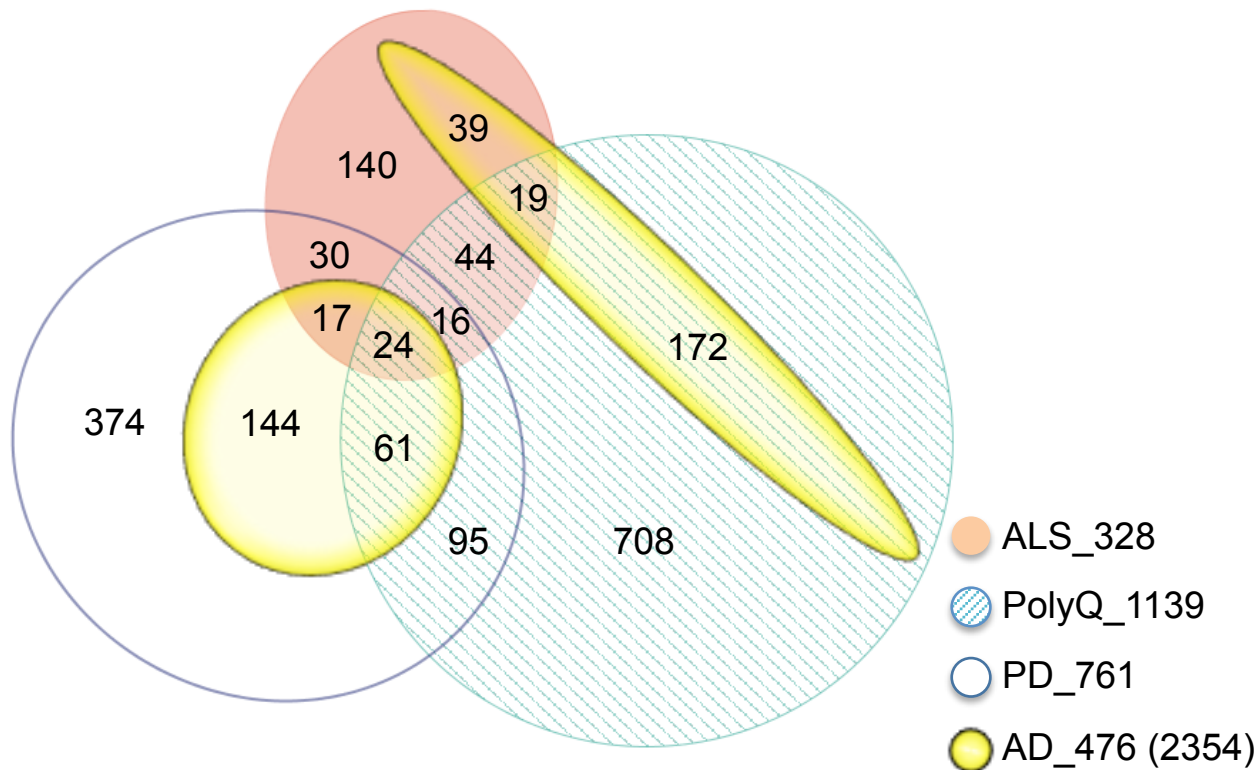

Supplement: Supplementary file 11 [file Image6.pdf]

Figure S8. % Overlap of NeuroD Sets (+ or – HTT Interactome) with HmSP and Exosome Datasets

A.

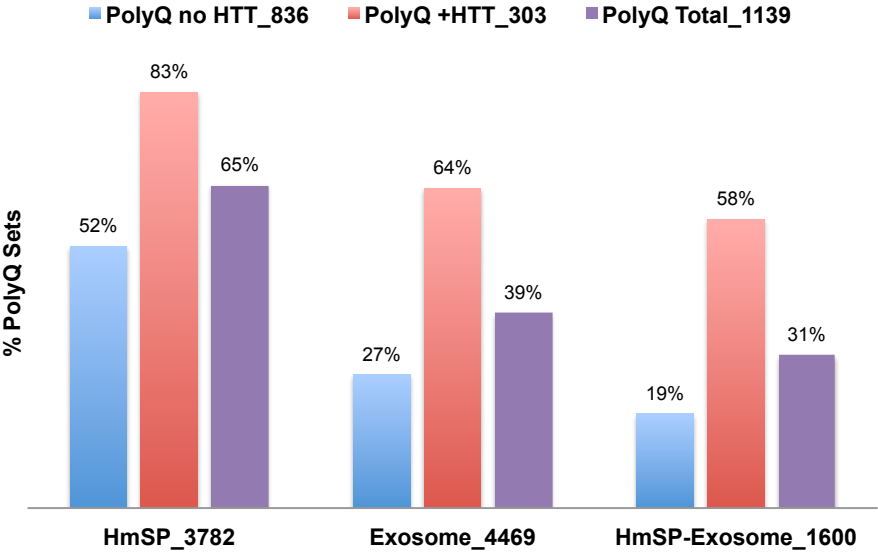

B.

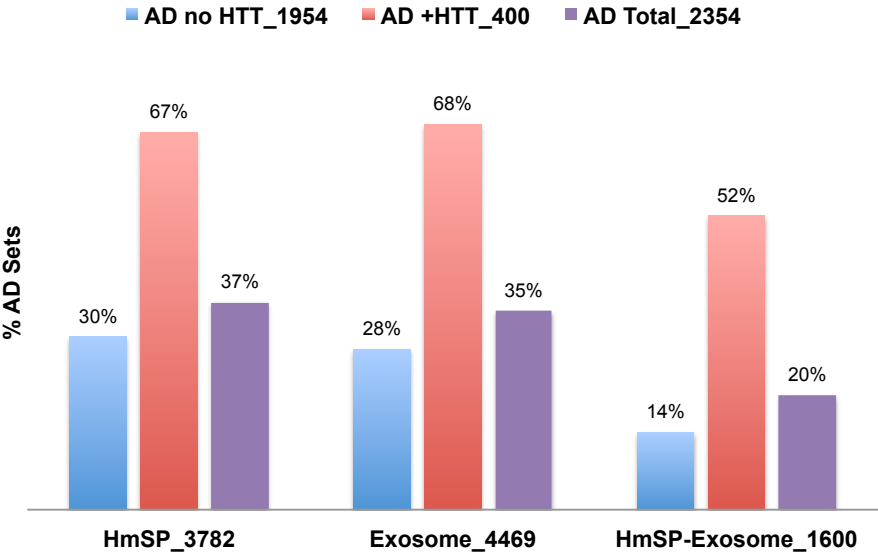

C.

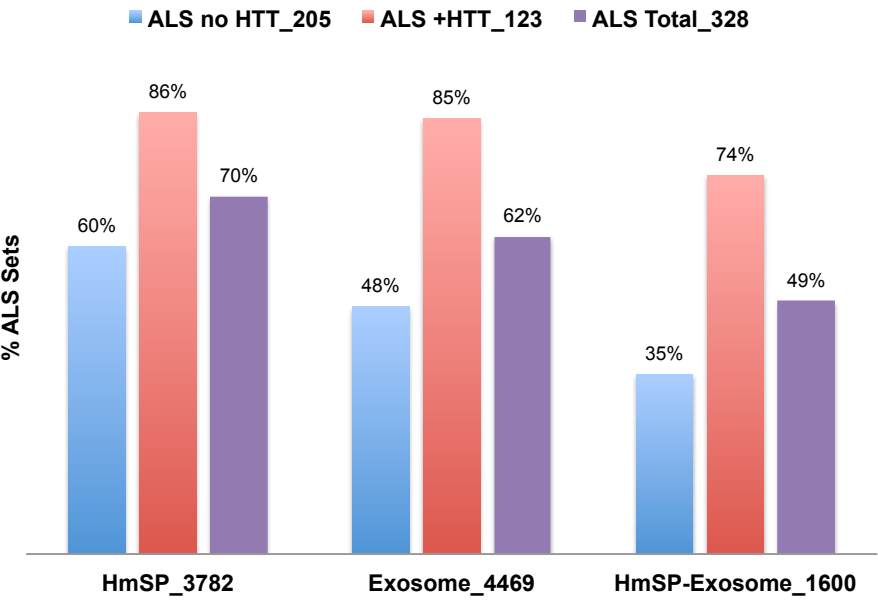

Supplement: Supplementary file 13 [file Image8.pdf]

Figure S10. % Overlap of HD, NeuroD with Synaptic Transcript (+ or – the HmSP and Exosome)

A.

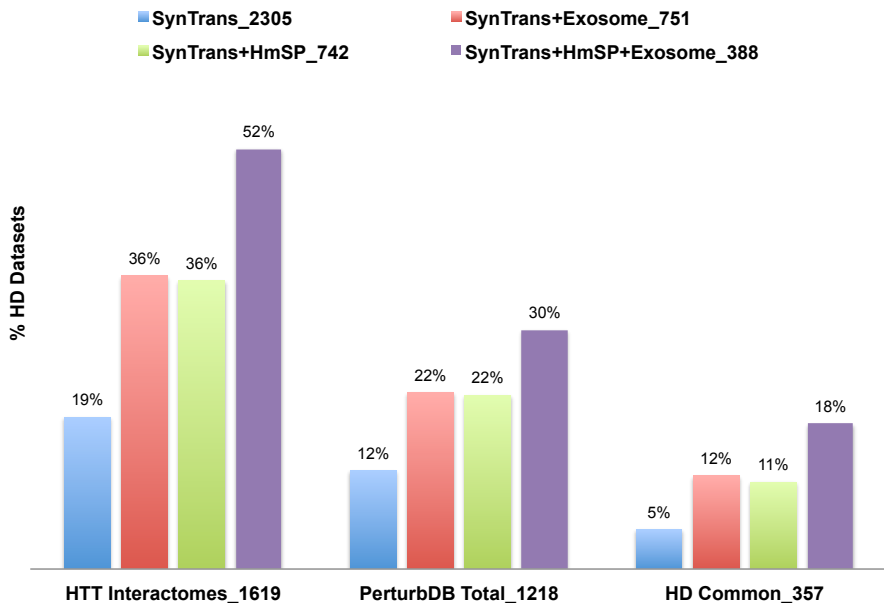

B.

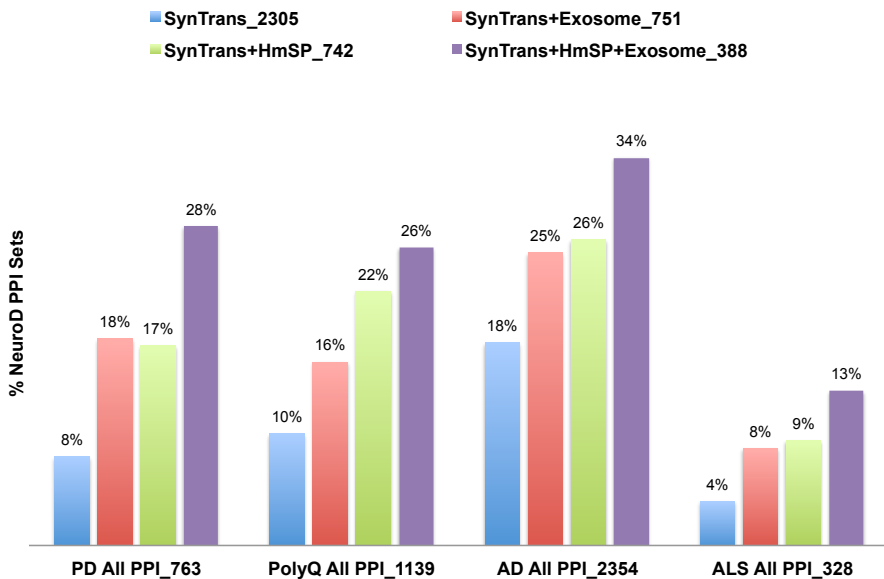

Supplement: Supplementary file 15 [file Image10.pdf]

Figure S11

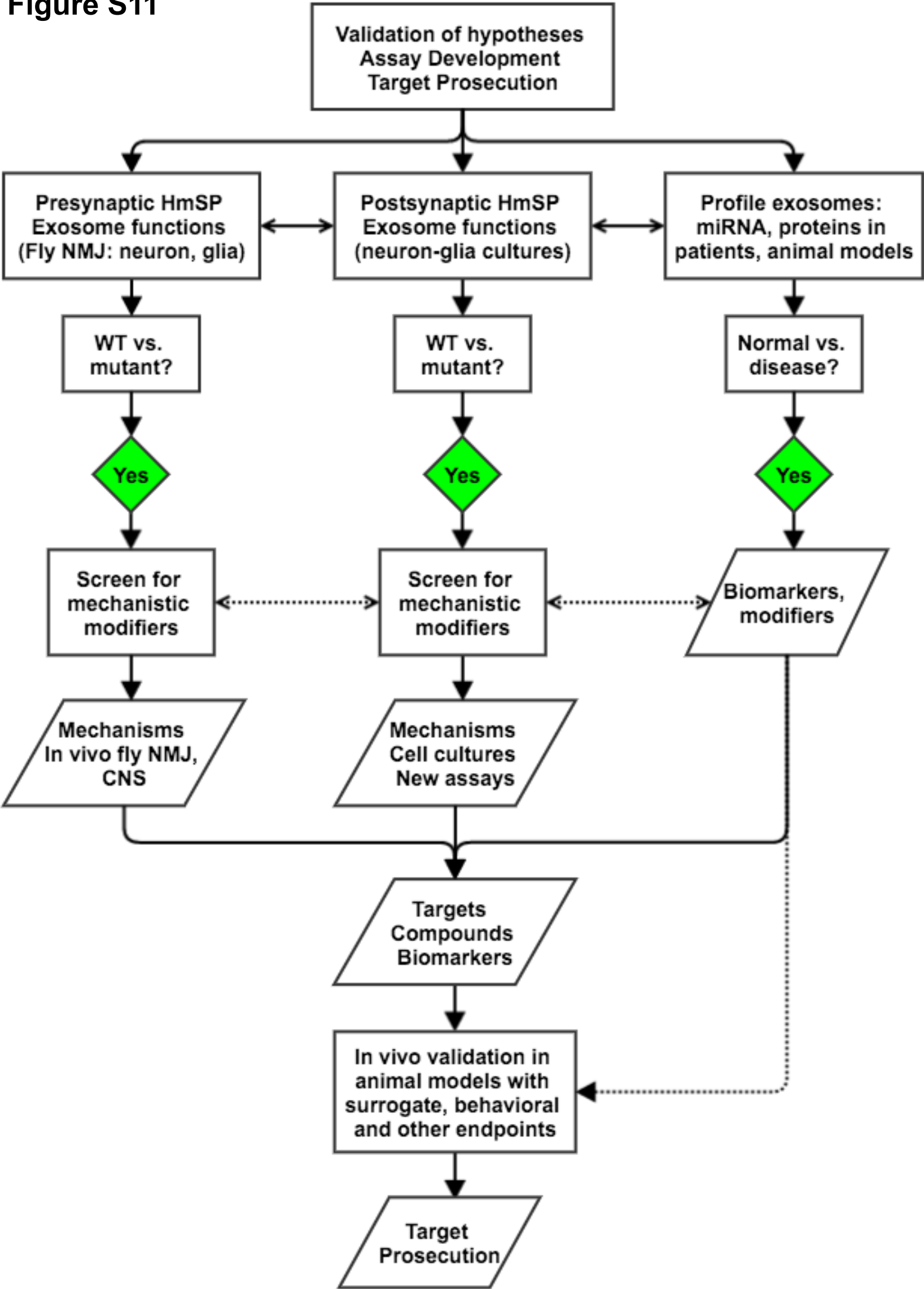

Supplement: Supplementary file 16 [file Image11.pdf]
