# Supplementary material for: Exosomes and Homeostatic Synaptic Plasticity Are Linked to Each other and to Huntington's, Parkinson's, and Other Neurodegenerative Diseases by Database-Enabled Analyses of Comprehensively Curated Datasets
Source: Front Neurosci. 2017 Mar 31;11:149. doi: 10.3389/fnins.2017.00149 (PMC5374209; doi:10.3389/fnins.2017.00149)

Figure S7. Overlap of NeuroD Sets with each other
In common (A) or not in common (B) with HTT Interactome

A + HTT Interactome

B No HTT Interactome

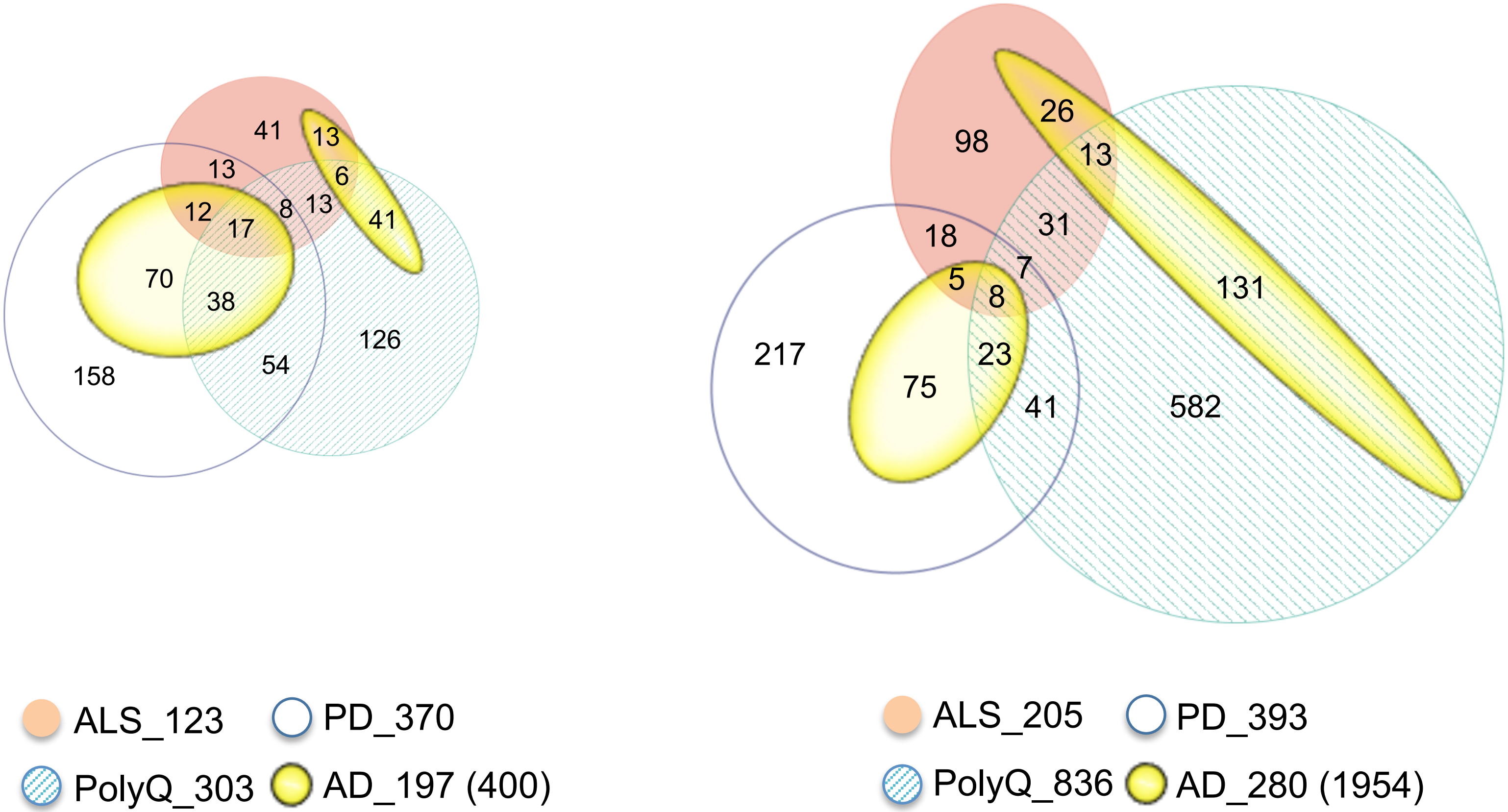

Supplement: Supplementary file 12 [file Image7.pdf]
