# Supplementary material for: Exosomes and Homeostatic Synaptic Plasticity Are Linked to Each other and to Huntington's, Parkinson's, and Other Neurodegenerative Diseases by Database-Enabled Analyses of Comprehensively Curated Datasets
Source: Front Neurosci. 2017 Mar 31;11:149. doi: 10.3389/fnins.2017.00149 (PMC5374209; doi:10.3389/fnins.2017.00149)

Figure S9. % Overlap of HmSP and Exosome Datasets with NeuroD Sets (+ or – HTT Interactome)

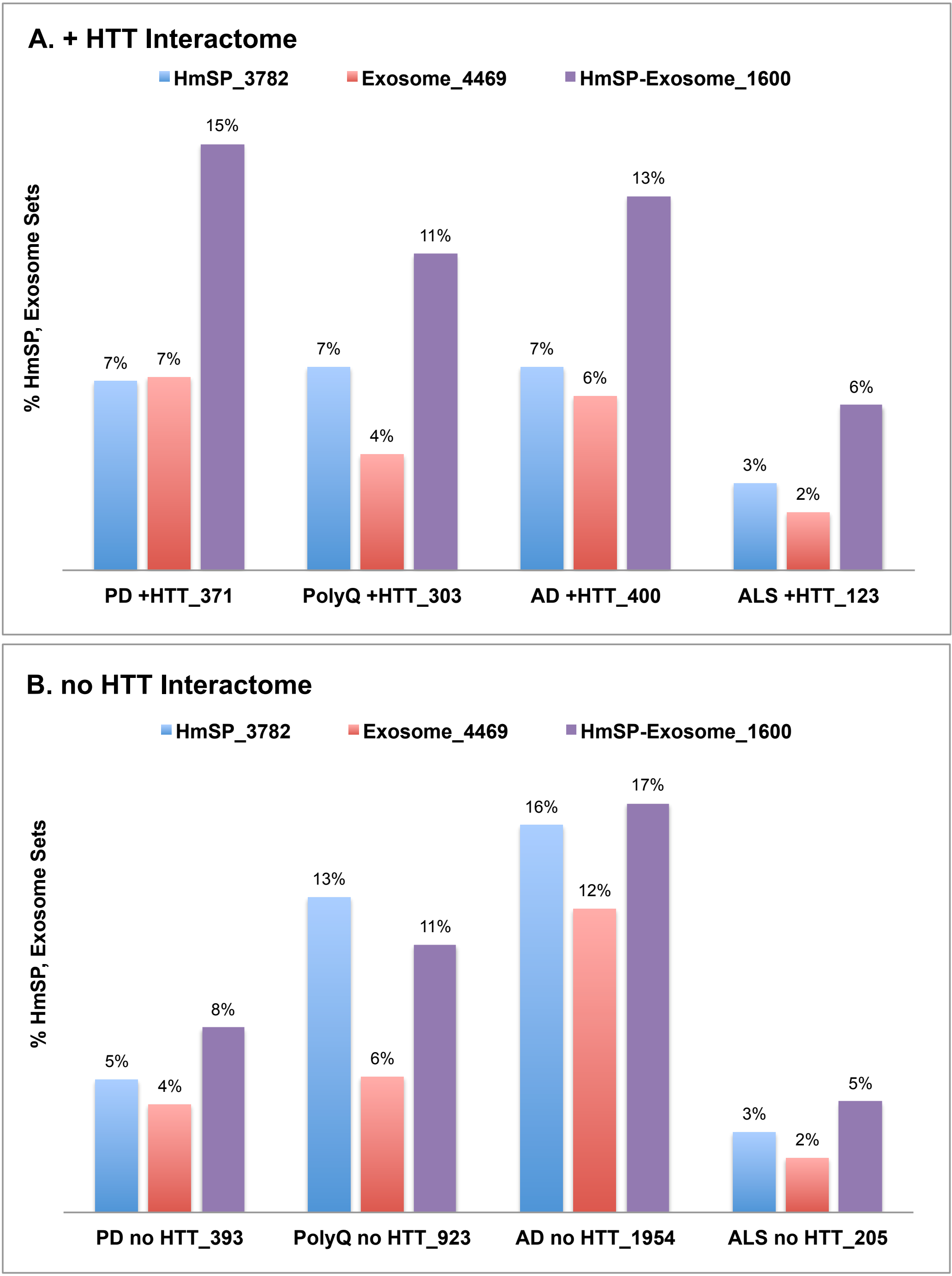

Supplement: Supplementary file 14 [file Image9.pdf]
